# MicroRNAs associated to single drug components of R-CHOP identifies diffuse large B-cell lymphoma patients with poor outcome and adds prognostic value to the international prognostic index

**DOI:** 10.1186/s12885-020-6643-8

**Published:** 2020-03-20

**Authors:** Hanne Due, Rasmus Froberg Brøndum, Ken H. Young, Martin Bøgsted, Karen Dybkær

**Affiliations:** 1 Department of Hematology, Aalborg University Hospital, Sdr. Skovvej 15, DK-9000 Aalborg, Denmark; 2 Department of Clinical Medicine, Aalborg University, Aalborg, Denmark; 3 Clinical Cancer Research Center, Aalborg University Hospital, Aalborg, Denmark; 4 Duke University Medical Center, Division of Hematopathology and Department of Pathology, Durham, NC USA

**Keywords:** Diffuse large B-cell lymphoma (DLBCL), Drug response, miRNA, Prognosis, Classification

## Abstract

**Background:**

Treatment resistance is a major clinical challenge of diffuse large B-cell lymphoma (DLBCL) where approximately 40% of the patients have refractory disease or relapse. Since DLBCL is characterized by great clinical and molecular heterogeneity, the purpose of the present study was to investigate whether miRNAs associated to single drug components of R-CHOP can improve robustness of individual markers and serve as a prognostic classifier.

**Methods:**

Fifteen DLBCL cell lines were tested for sensitivity towards single drug compounds of the standard treatment R-CHOP: rituximab (R), cyclophosphamide (C), doxorubicin (H), and vincristine (O). For each drug, cell lines were ranked using the area under the dose-response curve and grouped as either sensitive, intermediate or resistant. Baseline miRNA expression data were obtained for each cell line in untreated condition, and differential miRNA expression analysis between sensitive and resistant cell lines identified 43 miRNAs associated to growth response after exposure towards single drugs of R-CHOP. Using the Affymetrix HG-U133 platform, expression levels of miRNA precursors were assessed in 701 diagnostic DLBCL biopsies, and miRNA-panel classifiers predicting disease progression were build using multiple Cox regression or random survival forest. Classifiers were validated and ranked by repeated cross-validation.

**Results:**

Prognostic accuracies were assessed by Brier Scores and time-varying area under the ROC curves, which revealed better performance of multivariate Cox models compared to random survival forest models. The Cox model including miR-146a, miR-155, miR-21, miR-34a, and miR-23a~miR-27a~miR-24-2 cluster performed the best and successfully stratified GCB-DLBCL patients into high- and low-risk of disease progression. In addition, combination of the Cox miRNA-panel and IPI substantially increased prognostic performance in GCB classified patients.

**Conclusion:**

As a proof of concept, we found that expression data of drug associated miRNAs display prognostic utility and adding these to IPI improves prognostic stratification of GCB-DLBCL patients treated with R-CHOP.

## Background

Diffuse large B-cell lymphoma (DLBCL) is the most common type of malignant lymphoma, accounting for 30–40% of all newly diagnosed non-Hodgkin lymphomas. It is a highly aggressive and heterogeneous disease with respect to clinical presentation, tumor biology, and prognosis [1]. Gene expression profiling (GEP) enables cell-of-origin classification of DLBCL into two histologically indistinguishable molecular subclasses: the activated B-cell-like (ABC) and the germinal center B-cell-like (GCB), which reflect a subset of the normal B-cell differentiation stages. These subclasses differ in pathogenesis, genetic aberrations, and survival outcome [2, 3] and have entered clinical prognostic evaluation, complementing the international prognostic index (IPI), which has been the gold standard for decades [3, 4].

First-line treatment for newly diagnosed DLBCL patients is a multi-agent regimen combining the anti-CD20 monoclonal antibody rituximab with the three chemotherapeutics cyclophosphamide, doxorubicin, and vincristine, and the corticosteroid prednisone (R-CHOP). Although addition of rituximab to the regimen has improved treatment outcome of DLBCL substantially, up to 40% of patients have refractory disease or relapse after initial response to therapy due to drug-specific molecular resistance [5–7]. Study of the pharmacological principles underlying the R-CHOP regimen revealed no synergistic interaction but very low cross-resistance, showing a strong combination of independently effective drugs without overlapping mechanisms of resistance [8]. Thus, identification of biomarkers predictive for single drug components of R-CHOP is of great importance when attempting to improve clinical outcome.

microRNAs (miRNAs) are endogenous, small non-coding RNA molecules regulating gene expression at the post-transcriptional level [9]. Compelling evidence has demonstrated that miRNA expression is dysregulated in human cancers, with several miRNAs functioning as oncogenes or tumor suppressors [10]. Deregulation of miRNAs occurs early and consistently in tumor development and progression, and thus constitutes a promising source for discovery of novel biomarkers. Indeed, specific miRNAs and global miRNA expression profiles have shown significant potential as diagnostic as well as prognostic biomarkers for DLBCL [11–13], and several studies support their role in chemotherapy resistance [14–16]. Since DLBCL is a highly heterogeneous disease at the molecular level, we hypothesized that a panel of miRNAs associated to individual components of R-CHOP can improve robustness of individual markers and serve as a prognostic classifier predicting disease progression in DLBCL patients.

To test this hypothesis, we determined the global miRNA transcriptome and performed systematic dose-response drug screens in a DLBCL-specific cell line panel to identify single drug associated miRNAs. Applying multivariate Cox regression and random survival forest techniques, prognostic miRNA-panel classifiers were developed and the predictive accuracies were subsequently evaluated by Brier scores and time varying area under the ROC curves.

## Methods

### Cell lines

Fifteen human DLBCL-derived cell lines DB, NU-DHL-1, NU-DUL-1, MC-116, SU-DHL-4, SU-DHL-5 (DSMZ, German Collection of Microorganisms and Cell Cultures) and FARAGE, HBL-1, OCI-Ly3, OCI-Ly7, OCI-Ly8, OCI-Ly19, RIVA, SU-DHL-8, and U2932 (Provided by Dr. Jose A. Martinez-Climent, Molecular Oncology Laboratory, University of Navarra, Pamplona, Spain) were included (Table 1). The cell lines were cultured under standard conditions at 37 °C in humidified atmosphere of 95% air and 5% CO_2_ with RPMI1640 medium containing 10% fetal bovine serum (FBS) and 1% penicillin/streptomycin (P/S) for no longer than 20 passages. All cell lines were authenticated by DNA barcoding as previously described [18] and examined for mycoplasma infection when terminating their culturing period.

**Table 1:** Cell line specifications

Cell line	Seeding concentration	Rituximab		Cyclophosphamide		Doxorubicin		Vincristine		ABC/GCB
	cells/mL	AUC		AUC		AUC		AUC		
DB	0.125 × 10^6^	318.1	Int	345.6	Res	275.5	Res	130.7	Res	GCB
FARAGE	0.25 × 10^6^	344.1	Int	311.0	Res	179.6	Sen	56.1	Sen	GCB
HBL-1	0.25 × 10^6^	421.9	Res	226.0	Int	272.2	Int	84.5	Int	ABC
MC-116	0.25 × 10^6^	NA	NA	NA	NA	271.8	Int	62.0	NA*	GCB
NU-DHL-1	0.125 × 10^6^	316.6	Int	171.7	Sen	200.6	Sen	NA	NA	ABC
NU-DUL-1	0.25 × 10^6^	236.4	Sen	214.1	Sen	223.9	Int	90.3	Int	UC
OCI-Ly3	0.125 × 10^6^	407.6	Res	261.7	Int	253.4	Int	74.8	Int	ABC
OCI-Ly7	0.125 × 10^6^	218.3	Res	257.9	Sen	324.9	Res	114.5	Int	GCB
OCI-Ly8	0.25 × 10^6^	406.6	Res	NA	NA	NA	NA	NA	NA	UC
OCI-Ly19	0.25 × 10^6^	400.4	Res	202.1	Sen	166.9	Sen	54.0	Sen	UC
RIVA	0.25 × 10^6^	275.8	Res	296.0	Int	326.7	Res	109.0	Res	ABC
SU-DHL-4	0.25 × 10^6^	150.4	Sen	NA	NA	288.8	Res	NA	NA	GCB
SU-DHL-5	0.25 × 10^6^	251.2	Sen	164.5	Sen	201.5	Sen	57.9	Sen	GCB
SU-DHL-8	0.5 × 10^6^	425.0	Res	270.6	Int	222.0	Sen	126.1	Res	GCB
U2932	0.4 × 10^6^	354.1	Int	329.8	Res	294.8	Res	85.3	Int	GCB

For each drug, DLBCL cell lines were ranked according to sensitivity based on area under the dose-response curve (AUC) for rituximab, cyclophosphamide, doxorubicin, and vincristine. Division into tertiles defines: Rituximab, 4 sensitive, 5 intermediate, 5 resistant; Cyclophosphamide, 4 sensitive, 4 intermediate, 4 resistant; Doxorubicin, 5 sensitive, 4 intermediate, 5 resistant; Vincristine, 4 sensitive, 4 intermediate, 4 resistant. Based on GEP, DLBCL cell lines were classified into ABC/GCB subclasses by Wright classification using published algorithms at hemaClass.org [17]. ABC, activated B-cell like; GCB, germinal center B-cell like; Int, intermediate; NA, not available; Res, resistant; Sen, sensitive; * excluded due to large variation between replicates

### Clinical cohorts

The study was conducted in accordance with the Declaration of Helsinki and tumor biopsies from 73 primary DLBCL patients were collected at time of diagnosis in accordance with the RetroGen research protocol, approved by the Health Ethic Committee of North Denmark Region (Approval jr. no. N-20140099). All patients were treated with R-CHOP according to standard protocols. This retrospective cohort is referred to as the AAU dataset. In addition, we used the following data sets from the National Center for Biotechnology Information Gene Expression Omnibus (GEO) repository: Lymphoma/Leukemia Molecular Profiling Project R-CHOP (LLMPP R-CHOP) (GSE10846) [19] and International DLBCL Rituximab-CHOP Consortium MD Anderson Project (IDRC) (GSE31312) [20]. All patients were classified into the molecular ABC/GCB subclasses using GEP (Table 2).

### Dose-response experiments

Dose-response screens with rituximab, cyclophosphamide, doxorubicin, and vincristine, respectively, were performed as described previously [18, 21]. Since cyclophosphamide is a prodrug that requires hepatic activation to produce its active metabolite, the synthetic oxazaphophorine derivate mafosfamide was used in the dose-response assays. As the pharmacological effect of R-CHOP has limited cross-resistance rather than synergism [8], single drug screens were used instead of combinations in order to identify miRNAs that were specifically associated to response for the individual components of the treatment regimen. The cells were seeded 24 h prior to addition of the drug using cell line specific seeding concentrations to ensure exponential growth for 48 h (Table 1). For rituximab dose-response screens, each cell line was subjected to 16 concentrations in serial 2-fold dilutions ranging from 133.3 μg/mL to 407 × 10^−5^μg/mL and 30 min after rituximab addition, normal pooled human AB serum (INR IPLA-SERAB, Novakemi AB, Sweden) was added to a final concentration of 20%. For cyclophosphamide, doxorubicin, and vincristine, the cell lines were exposed to 18 drug-specific concentrations in 2-fold dilutions starting from 80, 10, and 20 μg/mL, respectively [21]. The number of metabolic active cells was evaluated after 48 h of drug exposure using MTS assay (CellTiter 96 Aqueous One Solution Reagent, Promega, Madison, WI). Absorbance was measured at 492 nm using an Optima Fluorostar plate reader (BMG LAB-TECH, Ortenberg, Germany). All border wells were omitted from data analysis in order to avoid border effect. All drug screens were conducted with 3 replicates of each drug dose and with 3 biological replicates for each cell line. Since a fixed time of drug exposure were used, fast proliferating cells will appear more sensitive compared to slow proliferating ones. Therefore, area under the dose-response curve were used as summary statistic of the drug screens, making the results independent of cell line doubling time as it takes growth kinetic into account [21].

### Global miRNA and mRNA expression profiling

Total RNA was extracted using a modified protocol combining TRIzol Reagent (Invitrogen, Paisley, UK) and mirVana miRNA Isolation Kit (Ambion/ThermoFisher Scientific, Grand Island, NY) as previously described [22]. RNA quality and concentration was determined by Agilent 2100 Bioanalyzer analysis (Agilent Technologies, Santa Clara, CA) and NanoDrop ND-1000 spectrophotometer (ThermoFisher Scientific), respectively. miRNA expression profiling was performed using GeneChip miRNA 1.0.2 arrays (Affymetrix, Santa Clara, CA) according to the manufacturer’s protocol. The cell lines DB, FARAGE, OCI-Ly3, OCI-Ly7, OCI-Ly8, OCI-Ly19, NU-DHL-1, RIVA, and U2932 were prepared for hybridization using Flashtag HSR kit from Genesphere (Genesphere, Hatfield, PA) whereas HBL-1, MC-116, NU-DUL-1, SU-DHL-4, SU-DHL-5, and SU-DHL-8 were prepared using Fashtag Biotin HSR RNA labeling kit (Affymetrix) [14]. For GEP, RNA was labeled and hybridized to Affymetrix GeneChip Human Genome U133 (HG-U133) Plus 2.0 arrays, as described by the manufacturer. Generated miRNA and HG-U133 CEL files are deposited at NCBI GEO repository GSE72648 and GSE109027, respectively. The data comply with MIAME requirements [23].

### Experimental validation of miRNA expression in cell lines by digital droplet polymerase chain reaction

Two independent cDNA syntheses were conducted and pooled before amplification in digital droplet polymerase chain reaction (ddPCR) analysis. Each sample was analyzed in duplicate/triplicate using ddPCR assays and correlated to probes on U133 + 2; hsa-miR-146a (000468), hsa-miR-155 (002623), hsa-miR-21 (000397), hsa-miR-27a (000408) and hsa-miR-34a (000426). miRNA expression was normalized to RNU6B (001093) and RNU24 (001001) and log2 transformed prior to correlation to miRNA specific probes on U133 + 2 (232504_at for miR-146a, 22937_at for miR-155, 220990_s_at for miR-21, 1555847_a_at for miR-23a –miR-27a-miR-24-2, and 235571_at for miR-34a). Correlation coefficients shown in Supplementary Figure 5.

### Statistical analysis

All statistical analyses were performed with R version 3.5.1; an accompanying knitR document with detailed information on the analysis and package versions is supplied in Supplementary Document S2. Prior to statistical analysis, the array data were cohort-wise background corrected and normalized at probe level by robust multichip average (RMA) [24] implemented in the Bioconductor package *affy* v1.58 [25].

Differentially expressed miRNAs between DLBCL cells lines classified as sensitive and resistant for rituximab, cyclophosphamide, doxorubicin, and vincristine, respectively, were identified using the empirical Bayes method [26] implemented in the R package *limma* v3.36.3 [27]. Since cell lines were prepared for hybridization to the miRNA 1.0.2 microarray platform with different labeling kits [14], differential miRNA expression analyses were adjusted for possible confounding with a kit effect by including it as a covariate in the model. MiRNAs with absolute log-fold changes greater than two (|FC| > 2) were considered as differentially expressed and were included in the list of candidate miRNAs subjected for further analysis. The set of candidate miRNAs for development of the prognostic classifier was chosen by filtering the set of differentially expressed miRNAs against those detected by HG-U133 probe sets. For each candidate probe set, correlation analysis between miRNA 1.0.2 array and HG-U133 array was conducted to validate HG-U133 array-based miRNA expression assessment.

Three clinical data sets (Table 2) were combined into a meta-cohort for training and validation of the prognostic classifiers. Validation was performed by repeated cross-validation with 10 folds and 10 repeats rather than using an independent validation set, since only two large clinical cohorts were available and we wanted to investigate the potential of a model trained on a combined dataset. By repeating the cross-validation we were able to investigate the variation in prediction accuracy resulting from the randomization in the cross-validation folds.

**Table 2:** Patient characteristics

	IDRC	LLMPP R-CHOP	AAU
n	468	233	73
Gender			
Female	198 (42%)	99 (42%)	30 (41%)
Male	270 (58%)	134 (58%)	43 (59%)
Age			
Median	63	61	66
Range	18–92	17–92	20–87
IPI			
0–1	254 (54%)	94 (40%)	48 (66%)
2–5	168 (36%)	70 (30%)	21 (29%)
NA	46 (10%)	69 (30%)	4 (5%)
ABC / GCB			
ABC	199 (43%)	93 (40%)	32 (44%)
GCB	225 (48%)	107 (46%)	32 (44%)
UC	44 (9%)	33 (14%)	9 (12%)

Number of patients and percentage within cohort / variable. *ABC* activated B-cell-like, *GCB* germinal center B-cell-like, *IPI* International prognostic index, *NA* not available, *n* number of patients, *UC* unclassified

To compensate for cohort-wise technical batch effects, the ComBat function implemented in the R-package *sva* [28] was applied. Training of prognostic classifiers were performed for all DLBCL patients and for subsets of ABC and GCB classified patients, respectively. Progression-free survival (PFS) was chosen as the outcome, since it is a treatment evaluation parameter as closely as possible to the time of drug exposure and the tested miRNAs were all associated directly to drug specific response. Furthermore, overall survival (OS) was used for verification of findings.

The prognostic miRNA-panel models were identified and trained by both multivariate Cox regression or random survival forest with 1000 trees using either drug-specific probes alone or in combination with a dichotomized IPI score (IPI “0–1” and “2–5” for low and high risk, respectively). The random survival forest implementation from the R-package *randomForestSRC* v2.7.0 was used [29–31]. For Cox regression models, variable selection was performed by preselecting probes that had a statistically significant effect in univariate Cox regressions adjusted for study effects (*p* < 0.05). The preselection of probes was performed for each cross-validation fold and repetition to avoid sharing information between training and validation sets. Additionally, univariate Cox regression models using either IPI or age as input features were trained and used as a baseline comparison for the models including miRNA probes. The prognostic accuracies of the classifiers were validated by performing 10-fold cross-validation using either all DLBCL patients in the combined dataset, or the respective subsets of ABC or GCB classified patients. The models were ranked by the Brier score and time varying area under the ROC curves by comparing the predicted survival probability for each individual in the combined validation set from the cross-validations to the observed PFS and OS every half year from zero to five years.

The linear predictions from the Cox models in the cross-validation were used to get a score for each individual, by averaging across the scores from the 10 repeats of the cross-validation. By splitting these scores into tertiles Kaplan-Meier plots for low, intermediate, and high risk patients were generated within the respective cohorts and with significance evaluated by log-rank tests. Since more than 90% of events for both PFS and OS happened within the first 5 years Kaplan-Meier plots were restricted to this period. For all Kaplan-Meier analyses, significance threshold were set to 0.05.

## Results

### Identification of miRNAs associated with drug-specific response

**Fig. 1:**
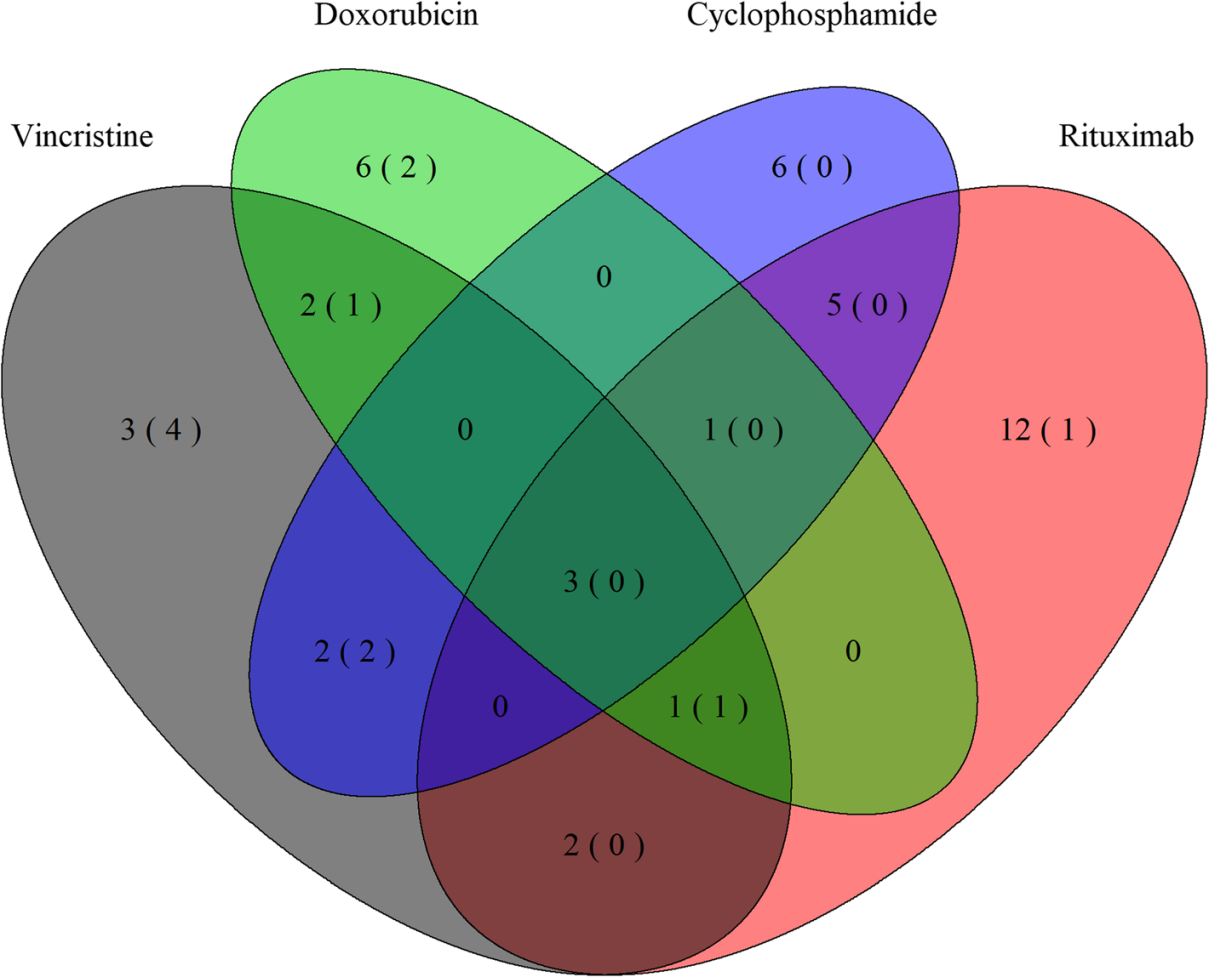
Drug response-specific miRNAs and HG-U133 probes. Venn-diagram depicting response specific differentially expressed miRNAs for rituximab, cyclophosphamide, doxorubicin, and vincristine. Numbers in parentheses show the number of matching HG-U133 Plus 2.0 probes. Figure was constructed using the R package VennDiagram v1.6.20

Triplicate dose-response experiments were analyzed using area under the dose-response curve for rituximab, cyclophosphamide, doxorubicin, and vincristine, respectively, taking the individual cell line doubling time into account [21]. For each drug, the cell lines were ranked according to their sensitivity, grouped into tertiles and categorized as sensitive, intermediate responsive, or resistant (Table 1). Global miRNA expression screens were conducted on untreated cell lines for optimal candidate selection and subsequent differential miRNA expression analysis between sensitive and resistant cell lines identified 43 miRNAs to be associated with compounds of the R-CHOP regimen (Supplementary Tables 1, 2, 3, and 4). The majority of miRNAs associated with vincristine and doxorubicin were downregulated in resistant DLBCL cells, whereas rituximab resistance was primarily associated with upregulated miRNAs (Supplementary Tables 1, 3, and 4). Conversely, differentially expressed miRNAs for cyclophosphamide were equally distributed between up- and downregulation (Supplementary Table 2). Several miRNAs were associated with more than one drug (Fig. 1); of notice, 6 out of 13 miRNAs were shared between vincristine and doxorubicin. Additionally, miR-146a, miR-148a, miR-155, miR-221, and miR-222 displayed ambiguous association to responses of different compounds of the regimen.

Since the miRNA microarray platform is not used in clinical context, miRNAs were matched to HG-U133 microarray probe sets detecting miRNA encoding genes, which reduced the candidate list to 11 probe sets detecting the following 9 miRNAs: miR-146a, miR-155, miR-21, miR-23a~27a~ 24–2 cluster, miR-34a, miR-503, and let-7b, which cover drug specific miRNAs for all four drugs (Fig. 1, Table 3).

**Table 3:** Candidate miRNAs

Probe ID (HG-U133)	miRNA	Drug sensitivity	Annotation grade
232504_at	miR-146a	↓ Cyc, ↑ Vin	A
238225_at	miR-146a	↓ Cyc, ↑ Vin	B
229437_at	miR-155	↓ Rtx, ↑ Dox, ↑Vin	A
220990_s_at	miR-21	↑ Vin	A
229417_at	miR-21	↑ Vin	E
235317_at	miR-23a~miR-27a~miR-24-2	↑ Dox, ↑ Vin	B
1555847_a_at	miR-23a~miR-27a~miR-24-2	↑ Dox, ↑ Vin	A
235571_at	miR-34a	↑ Dox, ↑ Vin	A
1557342_a_at	let-7b	↑ Vin	A
241464_s_at	let-7b	↑ Vin	B
227488_at	miR-503	↓ Rtx	B

Differentially expressed miRNAs detected by HG-U133 Plus 2.0 probes were selected as candidate miRNAs. The list consisted of 11 probe sets detecting 9 miRNAs. The annotation grade was assessed by NetAffx (Affymetrix) a transcript assignment pipeline creating a relationship between GeneChip probe sets and current transcript record. The transcript assignment grades fall into five categories A-E that describe the quality of the direct evidence. Grade A is a matching probe set having nine or more probes matching transcript mRNA. Grade B transcript assignments have partial overlap between transcripts and target sequence. Grade E is given when no transcript is found. Abbreviations: ↑ and ↓ defines up- and downregulation, respectively, in drug sensitive cell lines. Cyc, cyclophosphamide; Dox, doxorubicin; Rtx, rituximab; Vin, vincristine

### miRNA-panel prognostic classifier

Based on the identified drug-specific miRNAs, multiple Cox regression and random survival forest models were used to build classifiers predicting disease progression in patients with DLBCL to test both parametric and non-parametric ensemble based survival models. Since ABC and GCB-classified patients display different miRNA expression patterns, pathogenesis and clinical outcome [2, 3, 11], the training and validation of prognostic models were conducted for all DLBCL patients and for ABC and GCB classified patients, respectively. Additionally, since IPI is well-established in the clinical setting, it was essential to evaluate if the prognostic miRNA classifiers added to the prognostic performance of IPI.

**Fig. 2:**
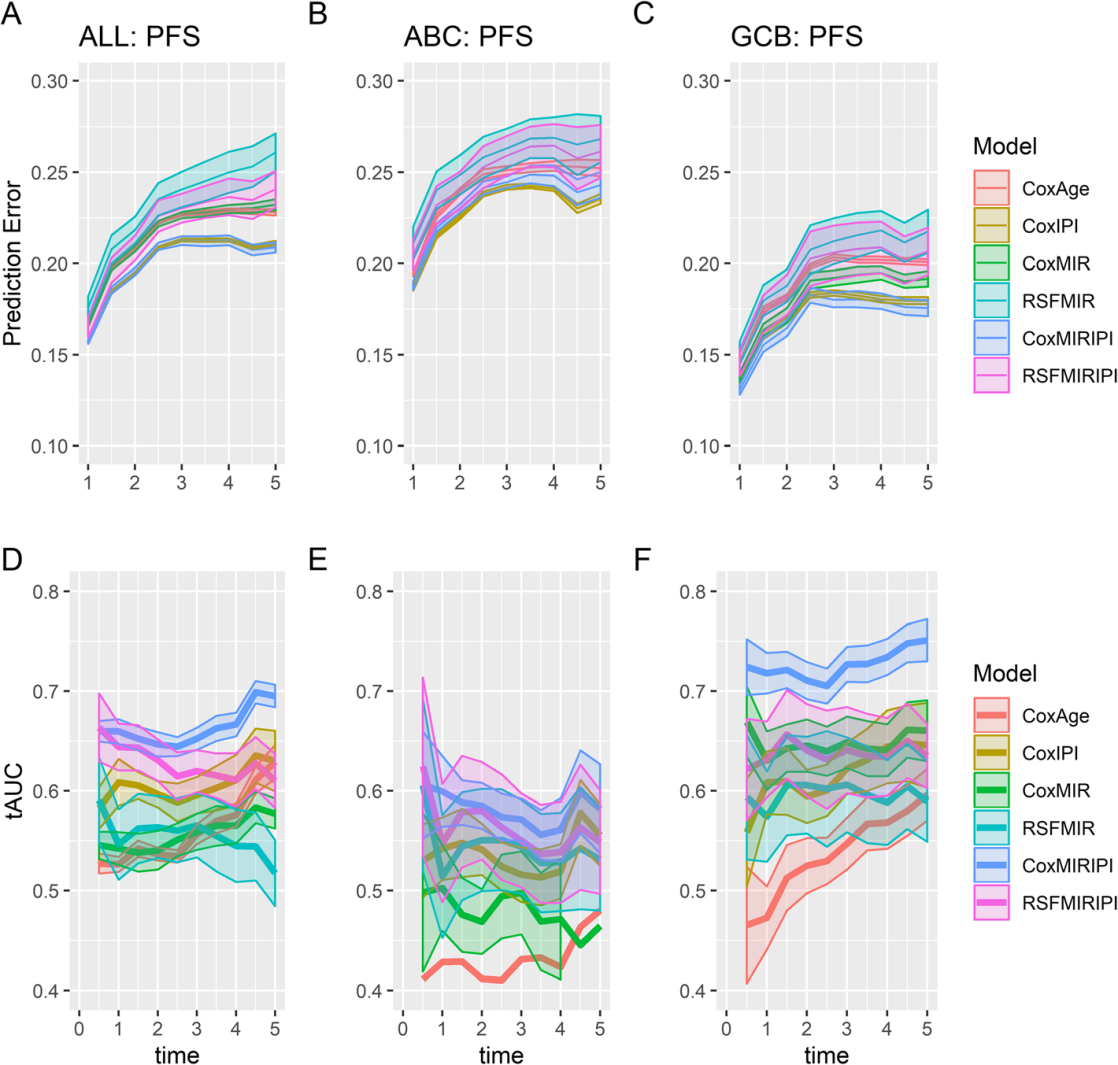
Evaluation of prognostic accuracy. Predicted survival from various prognostic classifiers vs observed progression free survival with the Brier score (top row, **a-c**) or tAUC (bottom row, **d-f**). Figures display means + − 2SD evaluated across the 10 cross-validation repetitions. The prognostic classifiers include: multivariate Cox regression models using either age (CoxAge), IPI (CoxIPI), miRNA expression (CoxMIR), or miRNA expression combined with IPI score (CoxMIRIPI), and random survival forest models using miRNA expression (RSFMIR) or miRNA expression in combination with IPI (RSFMIRIPI). Time in years. Figure was constructed using the R packages *ggplot2* v3.2.1 and *gridExtra* v2.3. ABC, activated B-cell-like; GCB, germinal center B-cell-like; IPI, international prognostic index; MIR, microRNA panel; PFS, progression-free survival; RSF, random survival forest; tAUC, time-varying are under the ROC curve

Prognostic accuracies of the generated miRNA-panel classifiers were assessed by Brier scores, which revealed better performance of the multivariate Cox models compared to the random survival forest models that had the largest prediction error regardless of input features (Fig. 2a-c). In contrast, evaluations of predictive performances by time varying area under the ROC curves were not as unambiguous, however, the highest predictive accuracy was still observed for multivariate Cox models (Fig. 2d-e). Comparison of analyses conducted for the respective cohorts (All DLBCL, ABC, and GCB patients) showed the lowest prediction errors for all models within the GCB subclass (Fig. 2a-c), with a multivariate Cox miRNA-panel model displaying prognostic utility comparable to IPI (Fig. 2f). In addition, combination of the miRNA-panel and IPI substantially increased prognostic performance in GCB classified patients (Fig. 2f), indicating a prognostic signal from the response-specific miRNAs independent of IPI. Furthermore, the Cox model combining IPI and the miRNA probe sets within the GCB subclass had the highest prognostic utility for all cross-validation repetitions showing a robust improvement.

In the ABC subclass, the developed miRNA-panel classifier did not provide additional prognostic information to IPI, either alone or in combination with IPI (Fig. 2b and e), suggesting little utility of the drug-specific miRNAs in this subclass. In agreement, similar results for ABC and GCB classified patients were observed when comparing the predicted PFS to the observed OS (Supplementary Figure 1). Consequently, the analyses were focused on the GCB subclass of DLBCL, which accounts for 46% of all cases (Table 2).

**Fig. 3:**
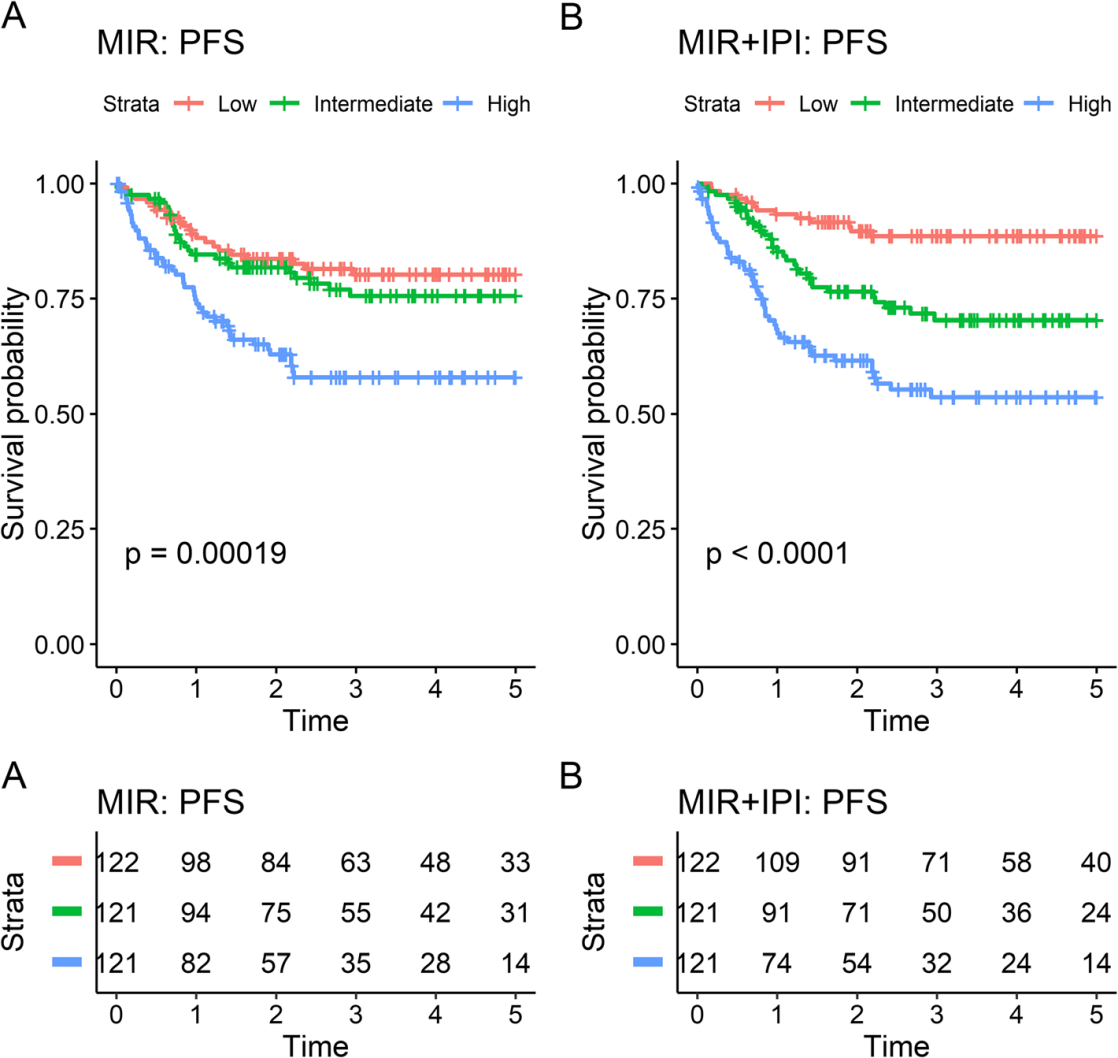
Predicted risk group vs 5-year progression free survival in the combined GCB dataset. Risk groups were obtained by taking the average predicted risk score across validation folds for the repeated cross-validation for the multivariate Cox models using (**a**) miRNA probes either alone or (**b**) in combination with IPI and splitting these into tertiles. Figure was constructed using the R package *survminer* v0.4.6

A linear prediction score was obtained for each individual in the combined GCB dataset by averaging the scores from the validation sets across the 10 repetitions of cross-validation for the multivariate Cox models using miRNA probe sets alone or in combination with IPI. These scores were used to rank the individual risk for all GCB classified patients, and by splitting the scores into tertiles, all GCB DLBCL patients were classified in defined groups of low, intermediate, and high-risk, the latter with significantly inferior prognosis as shown in the Kaplan-Meier plots for PFS and OS (Fig. 3, Supplementary Figure 2). The low and intermediate-risk group are not very distinct for the model including only miRNA probe sets, addition of IPI, however, clearly separates the patients into the distinct risk groups. The prognostic potential of the miRNA-panel was tested in each individual dataset validating the findings of inferior survival of patients with high-risk score (Supplementary Figure 3a-d).

**Fig. 4:**
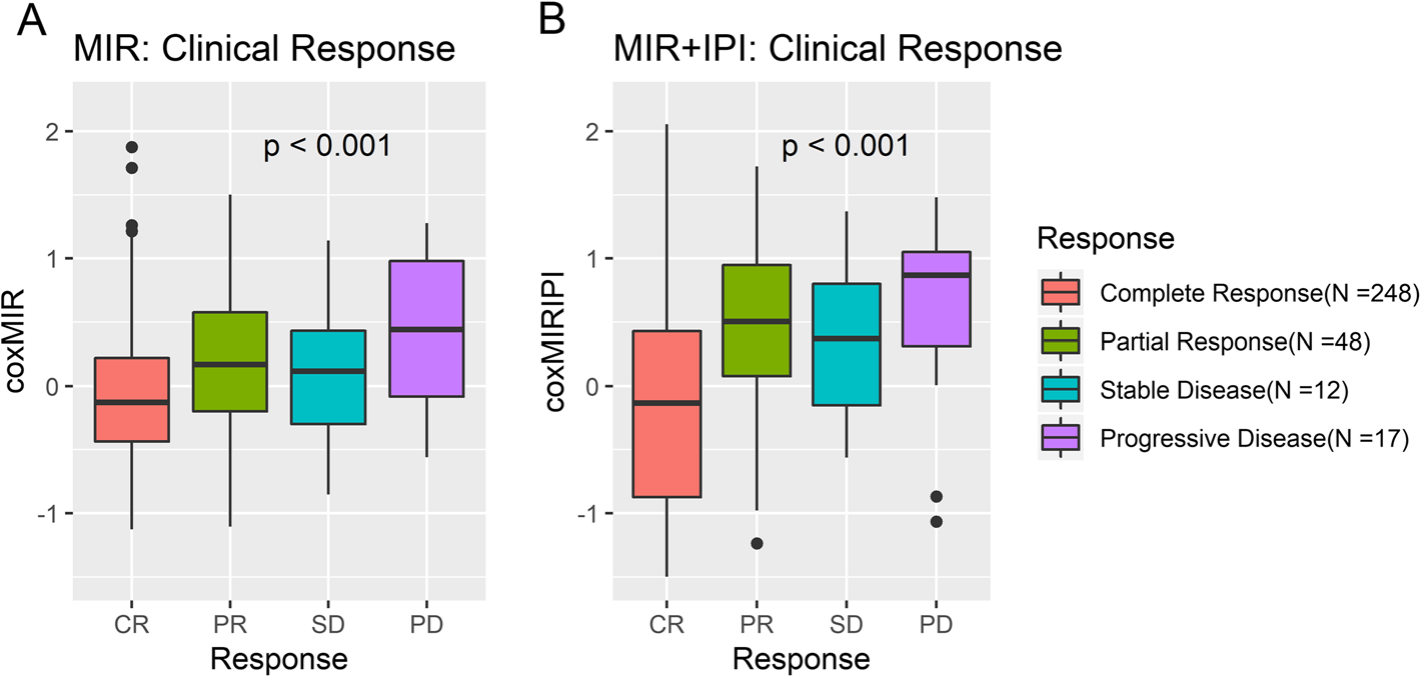
Risk scores in Cheson response evaluation classes. Response evaluations of GCB classified patients were investigated for association to predicted risk scores. CR, complete remission; PD, progressive disease; PR, partial response; SD, stable disease. Risk scores were obtained by taking the average predicted risk score across validation folds for the repeated cross-validation for the multivariate Cox models using miRNA probes either alone or in combination with IPI. Figure was constructed using the R packages *ggplot2* v3.2.1 and *gridExtra* v2.3

In accordance, GCB DLBCL patients with stable or progressive disease at time of response evaluation [32] display higher risk scores (Fig. 4), with the biggest difference in mean risk scores for the model including IPI. In addition, the multivariate Cox regression model combining IPI and probe sets detecting drug-specific miRNAs (miR-146a, miR-155, miR-21, miR-34a, and the miR-23a~miR-27a~miR-24-2 cluster) displayed the strongest prognostic performance (Fig. 2c and f) and was selected as the best model. Selected features and coefficients of the developed prognostic classifier are presented in Table 4, showing that most of the prognostic signal is carried by IPI, and that some of the probes have insignificant signal, although they were significant in the univariate analysis (Supplementary Table 5). Six of the candidate probe sets were not included in the final model, as they did not display significant effect in univariate Cox regression analysis (Supplementary Table 5).

**Table 4:** Selected features and corresponding coefficients for the final multivariate Cox model

Feature	miRNA	Hazard Ratio
IPI2–5		3.335
232504_at	miR-146a	0.857
229437_at	miR-155	0.868
220990_s_at	miR-21	0.995
1555847_a_at	miR-23a~miR-27a~miR-24-2	0.958
235571_at	miR-34a	0.776

The model was trained within the GCB subset of patients. MiRNA probes were pre-filtered to only include those significant in univariate Cox regression

Expression levels of the probe sets included in the final prognostic model (Table 4) were highly correlated to the mature miRNA measured by miRNA array (Supplementary Figure 4), supporting HG-U133 array-based miRNA expression assessment. Of notice, expression of the six miRNAs without significant effect were not correlated to the mature miRNA (Supplementary Figure 4), most likely due to low probe set specificity (Annotation Grade B and E, Table 3). For all drug-specific miRNAs a hazard ratio below 1 is observed (Table 4), indicating that higher expression is associated with better prognosis corresponding to the original observation of down-regulation being associated to resistance in vitro (Table 3). Additionally, it is evident that vincristine and doxorubicin are the main contributors to the prognostic miRNA signature as most of the miRNAs in the final classifier were initially included due to significant effect on doxorubicin and vincristine resistance.

## Discussion

Here, we combined global miRNA expression profiles and systematic dose-response screens to identify miRNAs associated to growth responses towards single drug components of the R-CHOP regimen. MiRNA-panel classifiers that assign individual DLBCL patients into low- and high-risk patients were generated utilizing two different approaches, multivariate Cox regression and random survival forest.

Several of the identified drug-specific miRNAs displayed ambiguous association to compounds of the R-CHOP regimen. Thus, high expression of miR-155 was associated with sensitivity to both doxorubicin and vincristine and with resistance to rituximab, most likely due to different drug mechanisms of actions and affected target genes. Noteworthy, doxorubicin and vincristine had 46% of the response specific miRNAs in common (miR-23a~miR-27a~miR-24, miR-34a, miR-155, and miR-222), indicating resistance mechanisms affecting the same molecular pathways. Consistently, ectopic induction of miR-34a have been shown to sensitize Ewing’s sarcoma cells to doxorubicin and vincristine [33].

Anti-tumor drugs often have several mechanisms of actions. Cyclophosphamide has, beside the function as an alkylating agent, been shown to induce cytokine release from the cancer cells, thereby attracting macrophages and facilitating antibody-mediated elimination [34]. Our in vitro drug screen system lacks stromal cells and the tumor microenvironment, thus, it is important to emphasize that the identified cyclophosphamide-specific miRNAs is restricted to the DNA crosslinking mechanism of action. Furthermore, prednisone is a synthetic corticosteroid drug suppressing the immune system without cytotoxic effects and consequently, not included in this study. The majority of the miRNAs included in the final prognostic miRNA signature are associated with doxorubicin and vincristine, which consistently, are the two major cornerstones for efficacy of R-CHOP [35].

Evaluation of prognostic impact in this study is based on retrospective clinical cohorts where treatment conditions are not strictly unified regarding number of cycles, dosing [19, 20] and individual dose reductions due to side-effects like neuropathy [36]. Taking this and the pharmacological information on no synergistic effect of R-CHOP into account [8], single drug screens were used rather than combinational assays to identify candidate miRNAs to be assessed in non-weighted models using either multivariate Cox regression or random survival forest models. The candidate miRNA originating from single drug screens are thus tested and evaluated for prognostic impacts in DLBCL patients receiving the full R-CHOP regimen, ensuring that only miRNAs with enough statistical power alone in univariate Cox-regression analysis and in modelled combinations are included in the final miRNA signature/panel.

The gene expression-based ABC/GCB subclasses of DLBCL rely on distinct oncogenic mechanisms [37, 38] and since affected miRNA targets vary depending upon the cell type and differentiation stage in which the miRNA is expressed [39], prognostic classifiers were generated separately in ABC and GCB subclasses. The drug-response specific miRNA-panel demonstrated a significant prognostic association with PFS and OS in GCB classified DLBCL patients treated with R-CHOP. Consistently, the complete response rate after R-CHOP was significantly higher for low-risk patients than high-risk, documenting association between miRNA expression and response to R-CHOP.

Of the seven miRNAs significant in univariate Cox regression, expression levels of miR-21, miR-146a, and miR-155 have been confirmed to differentiate the ABC/GCB subclasses in ≥4 studies [11, 12, 40–46], emphasizing the molecular heterogeneity between the two molecular subclasses of DLBCL. This difference in expression levels as well as the cell type specific effect of a particular miRNA, could be the reason why only little prognostic utility of the miRNA-panel was observed for ABC classified patients in contrast to GCB patients. Additionally, IPI displayed lower prognostic accuracy in these patients compared to GCB classified patients, indicating that new prognostic tools for ABC-DLBCL are needed.

The prognostic gold standard IPI is based solely on clinical parameters [4] and does not provide insight into the molecular pathways driving tumorigenesis and treatment resistance. Thus, molecular markers could potentially improve the prognostic accuracy of IPI and simultaneously provide information about the underlying molecular mechanisms. In line, combination of IPI with drug-specific miRNAs increased the prognostic accuracy in GCB-DLBCL. miRNAs display potential as a promising source of biomarkers as they, due to their small size, are relatively resistant to RNase degradation and are well-preserved in FFPE tissue [47]. Additionally, miRNAs can be detected in plasma and serum, thus, holding potential as liquid biomarkers [45, 48]. However, this study is proof of concept that addition of single drug-specific miRNAs to IPI improves prognostic stratification.

The prognostic utility of the miRNA probe sets was tested by training models using both the widely used semi-parametric multivariate Cox model, and the non-parametric ensemble based random survival forest. The Cox models relies on an assumption of proportional hazards and has a straightforward interpretation of parameters, whereas the random forest model makes no such assumptions and is able to fit more complex interactions among variables in the training data, but is more difficult to interpret. However, in our data we found no benefit of the random survival model compared to the Cox model. This might be caused by the limited number of variables which means that the individual survival trees are of limited depth, leading to too much bias in the predictions. Experimental validation of miRNA expression levels in cell lines are performed by ddPCR prior to correlation to miRNA specific probes on U133 + 2 (Supplementary Figure 4) generating coefficients of correlations ranging between 0.65 and 0.94 (Supplementary document S2, Table 5) supporting the usability of miRNAs in prognostic models with IPI.

A possible drawback of the current study is the lack of an independent external validation set, since the prediction accuracies from the cross-validation might be overestimated. The focus of the current study is, however, not the absolute but the relative prediction accuracy and thus ranking of different models. The ranking of the models could be affected by the randomization into cross-validation folds, results however showed that the multivariate cox model including both miRNA probe sets and IPI had superior prognostic signal within the GCB subgroup, regardless of cross-validation randomization, strengthening the hypothesis that miRNAs carry independent prognostic signal from IPI. However, further studies including an external validation set are needed before making recommendations on clinical application.

The miRNAs included in the final model are well studied miRNAs with known functions in normal B-cell differentiation and tumorigenesis [13, 49, 50]. miR-21 and miR-155 has been reported to be upregulated and to possess oncogenic properties in numerous cancers including breast cancer, glioblastoma, and DLBCL [51–55]. In addition, they have shown specific importance in the pathogenesis of DLBCL, highlighted by the fact that transgenic mice overexpressing miR-21 or miR-155 spontaneously develop lymphoma [56, 57]. In line, high expression of miR-21 is associated with inferior prognosis in DLBCL patients [55], and functional studies document a direct link between high miR-21 expression and CHOP resistance through regulation of PTEN [58]. miR-155 has been shown to control vincristine sensitivity in DLBCL cells through downregulation of Wee1 and clinical outcome analysis documented a significantly prolonged survival of GCB-classified DLBCL patient with high miR-155 expression [16].

## Conclusions

In conclusion, we found as proof of concept that adding gene expression data detecting drug-specific miRNAs to the clinically established IPI improved the prognostic stratification of GCB-DLBCL patients treated with R-CHOP.

## Supplementary information

**Additional file 1: Table 1.** Rituximab response-specific miRNAs. **Table 2.** Cyclophosphamide response-specific miRNAs. **Table 3.** Doxorubicin response-specific miRNAs. **Table 4.** Vincristine response-specific miRNAs. **Table 5.** Uni and multivariate cox regression analysis. **Figure 1.** Predicted survival from various prognostic classifiers vs observed overall survival with the brier score (top row) or time varying AUC (bottom row). **Figure 2.** Predicted risk group vs 5-year overall survival in the combined GCB dataset. **Figure 3.** Predicted risk group vs 5-year overall survival in GCB classified patients within each dataset: IDRC, LLMPPR-CHOP, and AAU. **Figure 4.** Correlation analysis between mature miRNA and miRNA encoding gene. **Figrue 5.** Correlation analysis between mature miRNA and miRNA encoding gene.

**Additional file 2.** MicroRNA associated to single drug components of R-CHOP identifies diffuse large B-cell lymphoma patients with poor outcome and adds prognostic value to the international prognostic index.

## Data Availability

The datasets supporting the conclusions of this article are available in the National Center for Biotechnology Information Gene Expression Omnibus (GEO) repository, GSE72648 (https://www.ncbi.nlm.nih.gov/geo/query/acc.cgi?acc=GSE72648) and GSE109027 (https://www.ncbi.nlm.nih.gov/geo/query/acc.cgi?acc=GSE109027). In addition, we used the following online available data sets: Lymphoma/Leukemia Molecular Profiling Project R-CHOP (GSE10846) and International DLBCL Rituximab-CHOP Consortium MD Anderson Project (GSE31312).

## Abbreviations

ABC: Activated B-cell-like
ddPCR: Digital droplet polymerase chain reaction
DLBCL: Diffuse large B-cell lymphoma
GCB: The germinal center B-cell-like
GEO: Gene Expression Omnibus
GEP: Gene expression profiling
IDRC: International DLBCL Rituximab-CHOP Consortium MD Anderson Project
IPI: International prognostic index
LLMPP R-CHOP: Lymphoma/Leukemia Molecular Profiling Project R-CHOP
miRNA: MicroRNA
OS: Overall survival
PFS: Progression-free survival
R-CHOP: Rituximab, cyclophosphamide, doxorubicin, vincristine, prednisone
RMA: Robust multichip average
tAUC: Time varying area under the ROC curves

## References

[1] Nogai H, Dörken B, Lenz G (2011). Pathogenesis of non-Hodgkin’s lymphoma. J Clin Oncol.

[2] Alizadeh AA, Eisen MB, Davis RE, Ma C, Lossos IS, Rosenwald A (2000). Distinct types of diffuse large B-cell lymphoma identified by gene expression profiling. Nature..

[3] Swerdlow SH, Campo E, Pileri SA, Harris NL, Stein H, Siebert R (2016). The 2016 revision of the World Health Organization classification of lymphoid neoplasms. Blood..

[4] Project TIN-HLPF (1993). A predictive model for aggressive non-Hodgkin’s lymphoma. N Engl J Med.

[5] Coiffier B, Lepage E, Briere J, Herbrecht R, Tilly H, Bouabdallah R (2002). CHOP chemotherapy plus rituximab compared with CHOP alone in elderly patients with diffuse large-B-cell lymphoma. N Engl J Med.

[6] Sehn LH, Donaldson J, Chhanabhai M, Fitzgerald C, Gill K, Klasa R (2005). Introduction of combined CHOP plus rituximab therapy dramatically improved outcome of diffuse large B-cell lymphoma in British Columbia. J Clin Oncol.

[7] Friedberg JW (2011). Relapsed/refractory diffuse large B-cell lymphoma. Hematol Am Soc Hematol Educ Progr..

[8] Palmer AC, Chidley C, Sorger PK. A curative combination cancer therapy achieves high fractional cell killing through low cross-resistance and drug additivity. Elife. 2019;8:e50036. 10.7554/eLife.50036. 10.7554/eLife.50036 PMC6897534 31742555

[9] Bartel DP (2004). MicroRNAs: genomics, biogenesis, mechanism, and function. Cell.

[10] Zhang B, Pan X, Cobb GP, Anderson TA (2007). microRNAs as oncogenes and tumor suppressors. Dev Biol.

[11] Iqbal J, Shen Y, Huang X, Liu Y, Wake L, Liu C (2015). Global microRNA expression profiling uncovers molecular markers for classification and prognosis in aggressive B-cell lymphoma. Blood.

[12] Due Hanne, Svendsen Pernille, Bødker Julie Støve, Schmitz Alexander, Bøgsted Martin, Johnsen Hans Erik, El-Galaly Tarec Christoffer, Roug Anne Stidsholt, Dybkær Karen (2016). miR-155 as a Biomarker in B-Cell Malignancies. BioMed Research International.

[13] Marques SC, Laursen MB, Bødker JS, Kjeldsen MK, Falgreen S, Schmitz A (2015). MicroRNAs in B-cells: from normal differentiation to treatment of malignancies. Oncotarget.

[14] Marques SC, Ranjbar B, Laursen MB, Falgreen S, Bilgrau AE, Bødker JS (2016). High miR-34a expression improves response to doxorubicin in diffuse large B-cell lymphoma. Exp Hematol.

[15] Rasmussen MH, Lyskjær I, Jersie-Christensen RR, Tarpgaard LS, Primdal-Bengtson B, Nielsen MM (2016). miR-625-3p regulates oxaliplatin resistance by targeting MAP2K6-p38 signalling in human colorectal adenocarcinoma cells. Nat Commun.

[16] Due H, Schönherz AA, Ryø L, Primo MN, Jespersen DS, Thomsen EA (2019). MicroRNA-155 controls vincristine sensitivity and predicts superior clinical outcome in diffuse large B-cell lymphoma. Blood Adv.

[17] Falgreen Steffen, Ellern Bilgrau Anders, Brøndum Rasmus Froberg, Hjort Jakobsen Lasse, Have Jonas, Lindblad Nielsen Kasper, El-Galaly Tarec Christoffer, Bødker Julie Støve, Schmitz Alexander, H. Young Ken, Johnsen Hans Erik, Dybkær Karen, Bøgsted Martin (2016). hemaClass.org: Online One-By-One Microarray Normalization and Classification of Hematological Cancers for Precision Medicine. PLOS ONE.

[18] Falgreen S, Dybkær K, Young KH, Xu-Monette ZY, El-Galaly TC, Laursen MB (2015). Predicting response to multidrug regimens in cancer patients using cell line experiments and regularised regression models. BMC Cancer.

[19] Lenz G, Wright G, Dave SS, Xiao W, Powell J, Zhao H (2008). Stromal gene signatures in large-B-cell lymphomas. N Engl J Med.

[20] Visco C, Li Y, Xu-Monette ZY, Miranda RN, Green TM, Li Y (2012). Comprehensive gene expression profiling and immunohistochemical studies support application of immunophenotypic algorithm for molecular subtype classification in diffuse large B-cell lymphoma: a report from the international DLBCL rituximab-CHOP Consortiu. Leukemia.

[21] Falgreen S, Laursen M, Bødker J, Kjeldsen M, Schmitz A, Nyegaard M (2014). Exposure time independent summary statistics for assessment of drug dependent cell line growth inhibition. BMC Bioinformatics.

[22] Dybkær K, Bøgsted M, Falgreen S, Bødker JS, Kjeldsen MK, Schmitz A (2015). Diffuse large B-cell lymphoma classification system that associates normal B-cell subset phenotypes with prognosis. J Clin Oncol.

[23] Brazma A, Hingamp P, Quackenbush J, Sherlock G, Spellman P, Stoeckert C (2001). Minimum information about a microarray experiment (MIAME)—toward standards for microarray data. Nat Genet.

[24] Irizarry RA, Hobbs B, Collin F, Beazer-Barclay YD, Antonellis KJ, Scherf U (2003). Exploration, normalization, and summaries of high density oligonucleotide array probe level data. Biostatistics.

[25] Gautier L, Cope L, Bolstad BM, Irizarry RA (2004). Affy--analysis of Affymetrix GeneChip data at the probe level. Bioinformatics.

[26] Phipson B, Lee S, Majewski IJ, Alexander WS, Smyth GK (2016). Robust hyperparameter estimation protects against hypervariable genes and improves power to detect differential expression. Ann Appl Stat.

[27] Ritchie ME, Phipson B, Wu D, Hu Y, Law CW, Shi W (2015). Limma powers differential expression analyses for RNA-sequencing and microarray studies. Nucleic Acids Res.

[28] Leek JT, Johnson WE, Parker HS, Fertig EJ, Jaffe AE, Storey JD, Zhang Y TL. sva: Surrogate Variable Analysis. R package version 3.28.0. 2018.

[29] Ishwaran H, Kogalur UB, Blackstone EH, Lauer MS (2008). Random survival forests. Ann Appl Stat.

[30] Ishwaran H, Kogalur UB (2007). Random survival forests for R. R News.

[31] Ishwaran H, Kogalur UB (2019). Fast unified random forests for survival, regression, and classification (RF-SRC).

[32] Cheson BD, Pfistner B, Juweid ME, Gascoyne RD, Specht L, Horning SJ (2007). Revised response criteria for malignant lymphoma. J Clin Oncol.

[33] Nakatani F, Ferracin M, Manara MC, Ventura S, Del Monaco V, Ferrari S (2012). miR-34a predicts survival of Ewing’s sarcoma patients and directly influences cell chemo-sensitivity and malignancy. J Pathol J Pathol.

[34] Pallasch CP, Leskov I, Braun CJ, Vorholt D, Drake A, Soto-Feliciano YM (2014). Sensitizing protective tumor microenvironments to antibody-mediated therapy. Cell..

[35] Wilson WH (2013). Treatment strategies for aggressive lymphomas: what works?. Hematol Am Soc Hematol Educ Progr.

[36] Madsen Marie Lindhard, Due Hanne, Ejskjær Niels, Jensen Paw, Madsen Jakob, Dybkær Karen (2019). Aspects of vincristine-induced neuropathy in hematologic malignancies: a systematic review. Cancer Chemotherapy and Pharmacology.

[37] Lenz G, Wright GW, Tolga Emre NC, Kohlhammer H, Dave SS, Davis RE, et al. Molecular subtypes of diffuse large B-cell lymphoma arise by distinct genetic pathways. 2008. www.pnas.org/cgi/content/full/. 10.1073/pnas.0804295105 PMC2533222 18765795

[38] Pasqualucci L, Trifonov V, Fabbri G, Ma J, Rossi D, Chiarenza A (2011). Analysis of the coding genome of diffuse large B-cell lymphoma. Nat Genet.

[39] Clark PM, Loher P, Quann K, Brody J, Londin ER, Rigoutsos I (2015). Argonaute CLIP-Seq reveals miRNA targetome diversity across tissue types. Sci Rep.

[40] Caramuta S, Lee L, Özata DM, Akçakaya P, Georgii-Hemming P, Xie H (2013). Role of microRNAs and microRNA machinery in the pathogenesis of diffuse large B-cell lymphoma. Blood Cancer J.

[41] Lawrie CH, Saunders NJ, Soneji S, Palazzo S, Dunlop HM, Cooper CDO (2008). MicroRNA expression in lymphocyte development and malignancy. Leukemia.

[42] Lawrie CH, Chi J, Taylor S, Tramonti D, Ballabio E, Palazzo S (2009). Expression of microRNAs in diffuse large B cell lymphoma is associated with immunophenotype, survival and transformation from follicular lymphoma. J Cell Mol Med.

[43] Malumbres R, Sarosiek KA, Cubedo E, Ruiz JW, Jiang X, Gascoyne RD (2009). Differentiation stage-specific expression of microRNAs in B lymphocytes and diffuse large B-cell lymphomas. Blood..

[44] Lawrie CH, Soneji S, Marafioti T, Cooper CDO, Palazzo S, Paterson JC (2007). Microrna expression distinguishes between germinal center B cell-like and activated B cell-like subtypes of diffuse large B cell lymphoma. Int J Cancer.

[45] Chen W, Wang H, Chen H, Liu S, Lu H, Kong D (2014). Clinical significance and detection of microRNA-21 in serum of patients with diffuse large B-cell lymphoma in Chinese population. Eur J Haematol.

[46] Zhong H, Xu L, Zhong J-H, Xiao F, Liu Q, Huang H-H (2012). Clinical and prognostic significance of miR-155 and miR-146a expression levels in formalin-fixed/paraffin-embedded tissue of patients with diffuse large B-cell lymphoma. Exp Ther Med.

[47] Li J, Smyth P, Flavin R, Cahill S, Denning K, Aherne S (2007). Comparison of miRNA expression patterns using total RNA extracted from matched samples of formalin-fixed paraffin-embedded (FFPE) cells and snap frozen cells. BMC Biotechnol.

[48] Jones K, Nourse JP, Keane C, Bhatnagar A, Gandhi MK (2014). Plasma microRNA are disease response biomarkers in classical Hodgkin lymphoma. Clin Cancer Res.

[49] Kurkewich JL, Hansen J, Klopfenstein N, Zhang H, Wood C, Boucher A (2017). The miR-23a~27a~24-2 microRNA cluster buffers transcription and signaling pathways during hematopoiesis. PLoS Genet.

[50] Thai T-H, Calado DP, Casola S, Ansel KM, Xiao C, Xue Y (2007). Regulation of the germinal center response by microRNA-155. Science..

[51] Qian B, Katsaros D, Lu L, Preti M, Durando A, Arisio R (2009). High miR-21 expression in breast cancer associated with poor disease-free survival in early stage disease and high TGF-β1. Breast Cancer Res Treat.

[52] Jiang S, Zhang H-W, Lu M-H, He X-H, Li Y, Gu H (2010). MicroRNA-155 functions as an OncomiR in breast cancer by targeting the suppressor of cytokine signaling 1 gene. Cancer Res.

[53] Yang CH, Yue J, Pfeffer SR, Fan M, Paulus E, Hosni-Ahmed A (2014). MicroRNA-21 promotes glioblastoma tumorigenesis by down-regulating insulin-like growth factor-binding protein-3 (IGFBP3). J Biol Chem.

[54] Marsigliante S, Storelli C, Mallardo M, Gianfreda CD, Montinaro A, D’Urso OF (2012). miR-155 is up-regulated in primary and secondary glioblastoma and promotes tumour growth by inhibiting GABA receptors. Int J Oncol.

[55] Go H, Jang J-Y, Kim P-J, Kim Y-G, Nam SJ, Paik JH (2015). MicroRNA-21 plays an oncogenic role by targeting FOXO1 and activating the PI3K/AKT pathway in diffuse large B-cell lymphoma. Oncotarget.

[56] Babar IA, Cheng CJ, Booth CJ, Liang X, Weidhaas JB, Saltzman WM (2012). Nanoparticle-based therapy in an in vivo microRNA-155 (miR-155)-dependent mouse model of lymphoma. Proc Natl Acad Sci U S A.

[57] Medina PP, Nolde M, Slack FJ (2010). OncomiR addiction in an in vivo model of microRNA-21-induced pre-B-cell lymphoma. Nature.

[58] Bai H, Wei J, Deng C, Yang X, Wang C, Xu R (2013). MicroRNA-21 regulates the sensitivity of diffuse large B-cell lymphoma cells to the CHOP chemotherapy regimen. Int J Hematol.

[59] Dybkær K, Due H, Brøndum RF, Young KH, Bøgsted M (2019). Addition of Drug-Response Specific Micro-RNAs to the International Prognostic Index Improves Prognostic Stratification of GCB-DLBCL Patients Treated with R-CHOP. Blood.

