# Supplementary material for: MicroRNAs associated to single drug components of R-CHOP identifies diffuse large B-cell lymphoma patients with poor outcome and adds prognostic value to the international prognostic index
Source: BMC Cancer. 2020 Mar 20;20:237. doi: 10.1186/s12885-020-6643-8 (PMC7082970; doi:10.1186/s12885-020-6643-8)
Supplement: Supplementary file 1 — Additional file 1: Table 1. Rituximab response-specific miRNAs. Table 2. Cyclophosphamide response-specific miRNAs. Table 3. Doxorubicin response-specific miRNAs. Table 4. Vincristine response-specific miRNAs. Table 5. Uni and multivariate cox regression analysis. Figure 1. Predicted survival from various prognostic classifiers vs observed overall survival with the brier score (top row) or time varying AUC (bottom row). Figure 2. Predicted risk group vs 5-year overall survival in the combined GCB dataset. Figure 3. Predicted risk group vs 5-year overall survival in GCB classified patients within each dataset: IDRC, LLMPPR-CHOP, and AAU. Figure 4. Correlation analysis between mature miRNA and miRNA encoding gene. Figrue 5. Correlation analysis between mature miRNA and miRNA encoding gene. [file 12885_2020_6643_MOESM1_ESM.docx]

Supplementary document S1

MicroRNAs associated to single drug components of R-CHOP identifies diffuse large B-cell lymphoma patients with poor outcome and adds prognostic value to the international prognostic index

Hanne Due, Rasmus Froberg Brøndum, Ken H. Young, Martin Bøgsted, and Karen Dybkær

Supplementary Tables

**Supplementary Table 1. Rituximab response-specific miRNAs.**

|  | logFC | AveExpr | t | P.Value | adj.P.Val | B |
| --- | --- | --- | --- | --- | --- | --- |
| hsa-miR-222_st | 6,127705 | 6,746617 | 2,335574 | 0,046563 | 0,99527 | -4,46427 |
| hsa-miR-221_st | 5,053428 | 6,674317 | 1,886941 | 0,094462 | 0,99527 | -4,50909 |
| hsa-miR-155_st | 3,043049 | 10,72422 | 1,965219 | 0,083584 | 0,99527 | -4,50105 |
| hsa-miR-345_st | 2,866339 | 4,783869 | 1,723372 | 0,121665 | 0,99527 | -4,52602 |
| hsa-miR-30a_st | 2,786987 | 4,874182 | 2,433865 | 0,039838 | 0,99527 | -4,45496 |
| hsa-miR-125b_st | 2,598253 | 4,129437 | 1,049439 | 0,323503 | 0,99527 | -4,59299 |
| hsa-miR-183_st | 2,526305 | 5,650952 | 3,008412 | 0,016116 | 0,99527 | -4,40543 |
| hsa-miR-503_st | 2,517528 | 3,58562 | 2,299829 | 0,04928 | 0,99527 | -4,4677 |
| hsa-miR-9-star_st | 2,441592 | 2,589669 | 3,016243 | 0,015921 | 0,99527 | -4,40481 |
| hsa-miR-551b_st | 2,399113 | 2,331537 | 1,782125 | 0,111144 | 0,99527 | -4,51992 |
| hsa-miR-27b_st | 2,294824 | 5,77071 | 2,15522 | 0,061967 | 0,99527 | -4,48188 |
| hsa-miR-424-star_st | 2,293201 | 3,463778 | 1,858482 | 0,098741 | 0,99527 | -4,51202 |
| hsa-miR-99a_st | 2,121462 | 3,714087 | 0,905464 | 0,390688 | 0,997061 | -4,60535 |
| hsa-miR-708_st | 2,108274 | 4,108474 | 1,155873 | 0,279847 | 0,99527 | -4,58322 |
| hsa-miR-886-5p_st | 2,069917 | 4,041259 | 0,988245 | 0,3509 | 0,997061 | -4,59838 |
| hsa-miR-886-3p_st | 2,059803 | 3,926248 | 1,353949 | 0,211383 | 0,99527 | -4,56395 |
| hsa-miR-30a-star_st | 2,039417 | 1,706873 | 1,795034 | 0,108949 | 0,99527 | -4,51859 |
| hsa-miR-629-star_st | -2,25157 | 3,931262 | -2,16803 | 0,060724 | 0,99527 | -4,48061 |
| hsa-miR-151-3p_st | -2,46828 | 6,287612 | -2,63478 | 0,028975 | 0,99527 | -4,43666 |
| hsa-miR-1303_st | -2,53781 | 2,774613 | -2,41659 | 0,040945 | 0,99527 | -4,45658 |
| hsa-miR-151-5p_st | -2,73673 | 9,110051 | -3,10579 | 0,013857 | 0,99527 | -4,39791 |
| hsa-miR-193b-star_st | -3,76472 | 4,193284 | -3,83514 | 0,004628 | 0,99527 | -4,34937 |
| hsa-miR-193b_st | -4,03679 | 7,623577 | -3,91546 | 0,004119 | 0,99527 | -4,3448 |
| hsa-miR-138_st | -4,16554 | 6,175924 | -3,0731 | 0,014576 | 0,99527 | -4,4004 |

Differentially expressed miRNAs detected comparing global miRNA expression profiles of rituximab sensitive and resistant DLBCL cell lines.

**Supplementary Table 2. Cyclophosphamide response-specific miRNAs.**

|  | logFC | AveExpr | t | P.Value | adj.P.Val | B |
| --- | --- | --- | --- | --- | --- | --- |
| hsa-miR-146a_st | 4,703233 | 9,474632 | 1,863339 | 0,103797 | 0,957447 | -4,41698 |
| hsa-miR-148a_st | 2,945592 | 5,477236 | 1,728867 | 0,126546 | 0,957447 | -4,58636 |
| hsa-miR-193b-star_st | 2,551795 | 4,381195 | 1,831364 | 0,108821 | 0,957447 | -4,45761 |
| hsa-miR-30a_st | 2,492496 | 3,816329 | 2,71736 | 0,029269 | 0,918181 | -3,29987 |
| hsa-miR-99b_st | 2,396544 | 2,20903 | 2,000733 | 0,084656 | 0,957447 | -4,24042 |
| hsa-miR-551b_st | 2,350764 | 2,459864 | 1,485161 | 0,180185 | 0,971399 | -4,88087 |
| hsa-miR-148a-star_st | 2,321438 | 2,687642 | 1,890166 | 0,099756 | 0,957447 | -4,38275 |
| hsa-miR-664-star_st | 2,152382 | 3,391757 | 2,159586 | 0,066825 | 0,957447 | -4,03327 |
| hsa-miR-152_st | 2,023634 | 4,542684 | 2,094523 | 0,073626 | 0,957447 | -4,11841 |
| hsa-miR-151-3p_st | -2,23555 | 5,757154 | -2,31506 | 0,053015 | 0,957447 | -3,8288 |
| hsa-miR-486-3p_st | -2,30555 | 2,473152 | -7,08054 | 0,000178 | 0,075391 | 0,853311 |
| hsa-miR-151-5p_st | -2,71017 | 7,972961 | -1,35084 | 0,21792 | 0,984429 | -5,03406 |
| hsa-miR-138_st | -3,66317 | 6,355507 | -1,66731 | 0,138474 | 0,957447 | -4,66244 |
| hsa-miR-486-5p_st | -4,4186 | 4,465431 | -7,28733 | 0,000148 | 0,075391 | 0,976114 |
| hsa-miR-221_st | -5,31488 | 7,620498 | -1,82912 | 0,109182 | 0,957447 | -4,46044 |
| hsa-miR-222_st | -5,98562 | 7,443487 | -1,72428 | 0,1274 | 0,957447 | -4,59206 |
| hsa-miR-708_st | -6,07315 | 4,649318 | -6,33909 | 0,000356 | 0,100596 | 0,367718 |

Differentially expressed miRNAs detected comparing global miRNA expression profiles of cyclophosphamide sensitive and resistant DLBCL cell lines.

**Supplementary Table 3. Doxorubicin response-specific miRNAs.**

|  | logFC | AveExpr | t | P.Value | adj.P.Val | B |
| --- | --- | --- | --- | --- | --- | --- |
| hsa-miR-222_st | -6,37335 | 6,110226 | -2,86841 | 0,01821 | 0,955019 | -4,35893 |
| hsa-miR-221_st | -5,51097 | 6,38203 | -2,95342 | 0,015839 | 0,955019 | -4,34936 |
| hsa-miR-34a_st | -3,28063 | 3,920212 | -2,12977 | 0,06153 | 0,955019 | -4,45114 |
| hsa-miR-27a_st | -2,84788 | 6,522453 | -3,52912 | 0,006252 | 0,955019 | -4,29097 |
| hsa-miR-708_st | -2,7983 | 4,150388 | -1,92045 | 0,08644 | 0,955019 | -4,47946 |
| hsa-miR-200c_st | -2,62599 | 5,556842 | -1,63585 | 0,135722 | 0,955019 | -4,51833 |
| hsa-miR-23a_st | -2,60387 | 9,104988 | -3,11672 | 0,012131 | 0,955019 | -4,33167 |
| hsa-miR-155_st | -2,1725 | 10,32635 | -1,5163 | 0,16318 | 0,955019 | -4,53447 |
| hsa-miR-129-5p_st | 2,009089 | 2,5239 | 2,751015 | 0,022094 | 0,955019 | -4,37252 |
| hsa-miR-193b-star_st | 2,192303 | 4,765981 | 1,659642 | 0,130781 | 0,955019 | -4,5151 |
| hsa-miR-1295_st | 2,240827 | 2,366998 | 2,595591 | 0,028558 | 0,955019 | -4,39119 |
| hsa-miR-181a-2-star_st | 2,466703 | 4,281511 | 1,525795 | 0,16083 | 0,955019 | -4,5332 |
| hsa-miR-125a-5p_st | 2,477203 | 3,493375 | 2,06706 | 0,068163 | 0,955019 | -4,45957 |

Differentially expressed miRNAs detected comparing global miRNA expression profiles of doxorubicin sensitive and resistant DLBCL cell lines.

**Supplementary Table 4. Vincristine response-specific miRNAs.**

|  | logFC | AveExpr | t | P.Value | adj.P.Val | B |
| --- | --- | --- | --- | --- | --- | --- |
| hsa-miR-886-5p_st | -2,03165 | 4,060709 | -1,62739 | 0,155239 | 0,94908 | -4,54467 |
| hsa-miR-27a_st | -2,08251 | 6,60642 | -1,83862 | 0,116057 | 0,94908 | -4,52719 |
| hsa-miR-708_st | -2,09384 | 4,1347 | -0,97634 | 0,36694 | 0,94908 | -4,59881 |
| hsa-miR-146a_st | -2,10943 | 9,790432 | -1,1267 | 0,303288 | 0,94908 | -4,58685 |
| hsa-miR-21-star_st | -2,28182 | 4,20414 | -3,37078 | 0,015242 | 0,94908 | -4,43104 |
| hsa-miR-886-3p_st | -2,32203 | 3,868368 | -2,28088 | 0,063129 | 0,94908 | -4,49342 |
| hsa-let-7b_st | -2,34635 | 9,877806 | -1,59086 | 0,163205 | 0,94908 | -4,54775 |
| hsa-miR-148a_st | -2,60258 | 4,725986 | -1,55092 | 0,172358 | 0,94908 | -4,55113 |
| hsa-miR-221_st | -2,81947 | 5,78796 | -1,0042 | 0,354391 | 0,94908 | -4,59665 |
| hsa-miR-34a_st | -3,14712 | 3,557925 | -1,65758 | 0,14894 | 0,94908 | -4,54214 |
| hsa-miR-21_st | -3,21691 | 4,197068 | -3,41433 | 0,014451 | 0,94908 | -4,42913 |
| hsa-miR-222_st | -3,85905 | 5,283139 | -1,20921 | 0,272469 | 0,94908 | -4,58005 |
| hsa-miR-155_st | -3,95284 | 10,24486 | -2,566 | 0,042918 | 0,94908 | -4,47421 |

Differentially expressed miRNAs detected comparing global miRNA expression profiles of vincristine sensitive and resistant DLBCL cell lines.

**Supplementary Table 5. Uni and multivariate cox regression analysis**

|  | miRNA | uni.HR | uni.conf | uni.p | multi.HR | multi.conf | multi.p |
| --- | --- | --- | --- | --- | --- | --- | --- |
| 238225_at | miR-146a | 0.92 | (0.49;1.71) | 0.792 | (0.61;2.14) | (0.61;2.14) | 0.669 |
| 232504_at | miR-146a | 0.69 | (0.58;0.81) | 0.000 | (0.65;1) | (0.65;1) | 0.047 |
| 229437_at | miR-155 | 0.75 | (0.66;0.85) | 0.000 | (0.73;1.04) | (0.73;1.04) | 0.120 |
| 220990_s_at | miR-21 | 0.74 | (0.62;0.88) | 0.001 | (0.79;1.39) | (0.79;1.39) | 0.760 |
| 229417_at | miR-21 | 0.90 | (0.55;1.46) | 0.666 | (0.54;1.42) | (0.54;1.42) | 0.591 |
| 235317_at | miR-23a | 0.98 | (0.64;1.5) | 0.932 | (0.8;2.29) | (0.8;2.29) | 0.257 |
| 1555847_a_at | miR-23a | 0.76 | (0.59;0.97) | 0.025 | (0.61;1.23) | (0.61;1.23) | 0.418 |
| 235571_at | miR-34a | 0.54 | (0.38;0.77) | 0.001 | (0.44;1.02) | (0.44;1.02) | 0.064 |
| 1557342_a_at | hsa-let-7b | 1.02 | (0.57;1.84) | 0.945 | (0.46;1.51) | (0.46;1.51) | 0.549 |
| 241464_s_at | hsa-let-7b | 0.73 | (0.33;1.58) | 0.422 | (0.35;1.62) | (0.35;1.62) | 0.464 |
| 227488_at | miR-503 | 1.11 | (0.62;1.98) | 0.726 | (0.57;1.93) | (0.57;1.93) | 0.875 |

Hazard ratio (HR), 95% confidence interval and p-value for uni- and multivariate Cox regression.

Supplementary Figures

**Supplementary figure 1:**
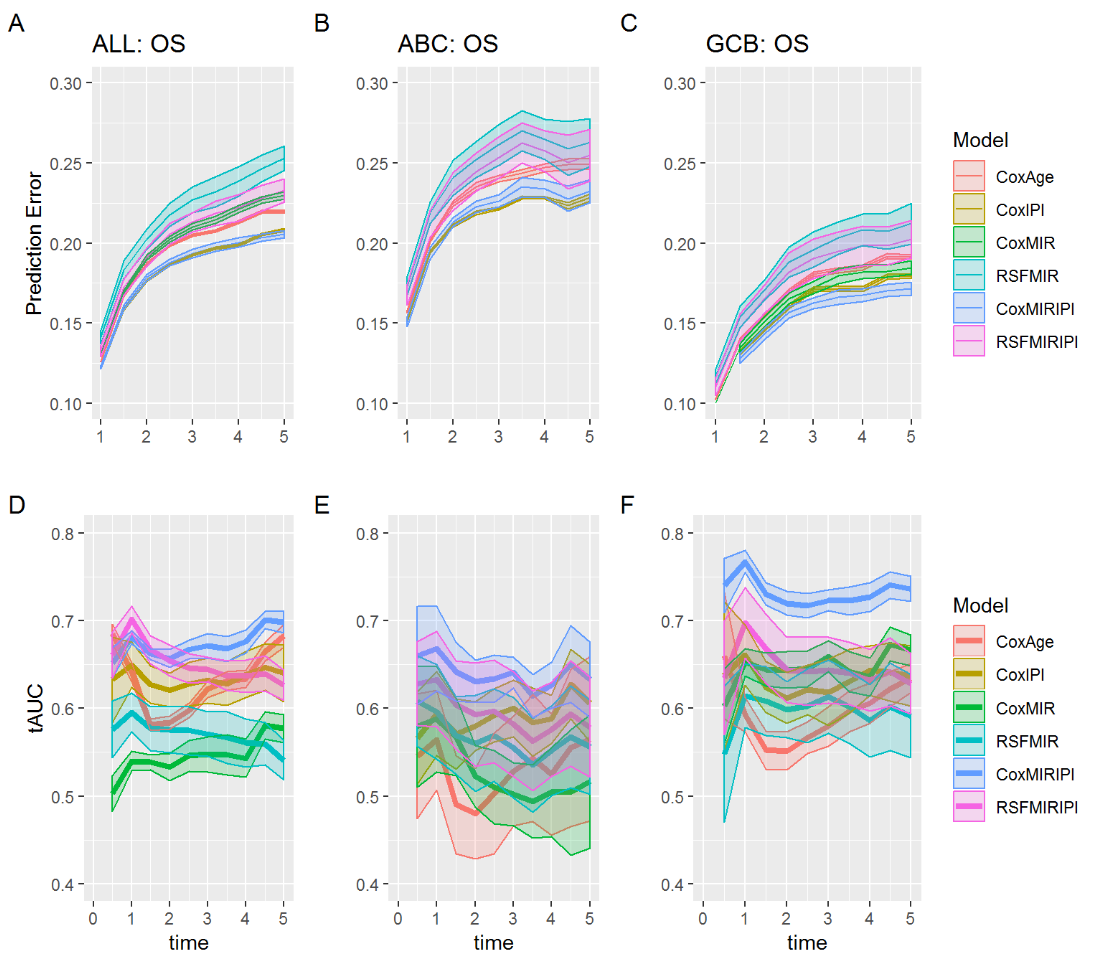


Predicted survival from various prognostic classifiers vs observed overall survival with the brier score (top row) or time varying AUC (bottom row). Figures display means +- 2SD evaluated across the 10 cross-validation repetitions. The prognostic classifiers include: multivariate Cox regression models using either age (CoxAge), IPI (CoxIPI), miRNA expression (CoxMIR), or miRNA expression combined with IPI score (CoxMIRIPI), and random survival forest models using miRNA expression (RSFMIR) or miRNA expression in combination with IPI (RSFMIRIPI). ABC, activated B-cell-like; GCB, germinal center B-cell-like; IPI, international prognostic index; MIR, microRNA panel; PFS, progression-free survival; RSF, random survival forest. Time in years.

**Supplementary figure 2:**


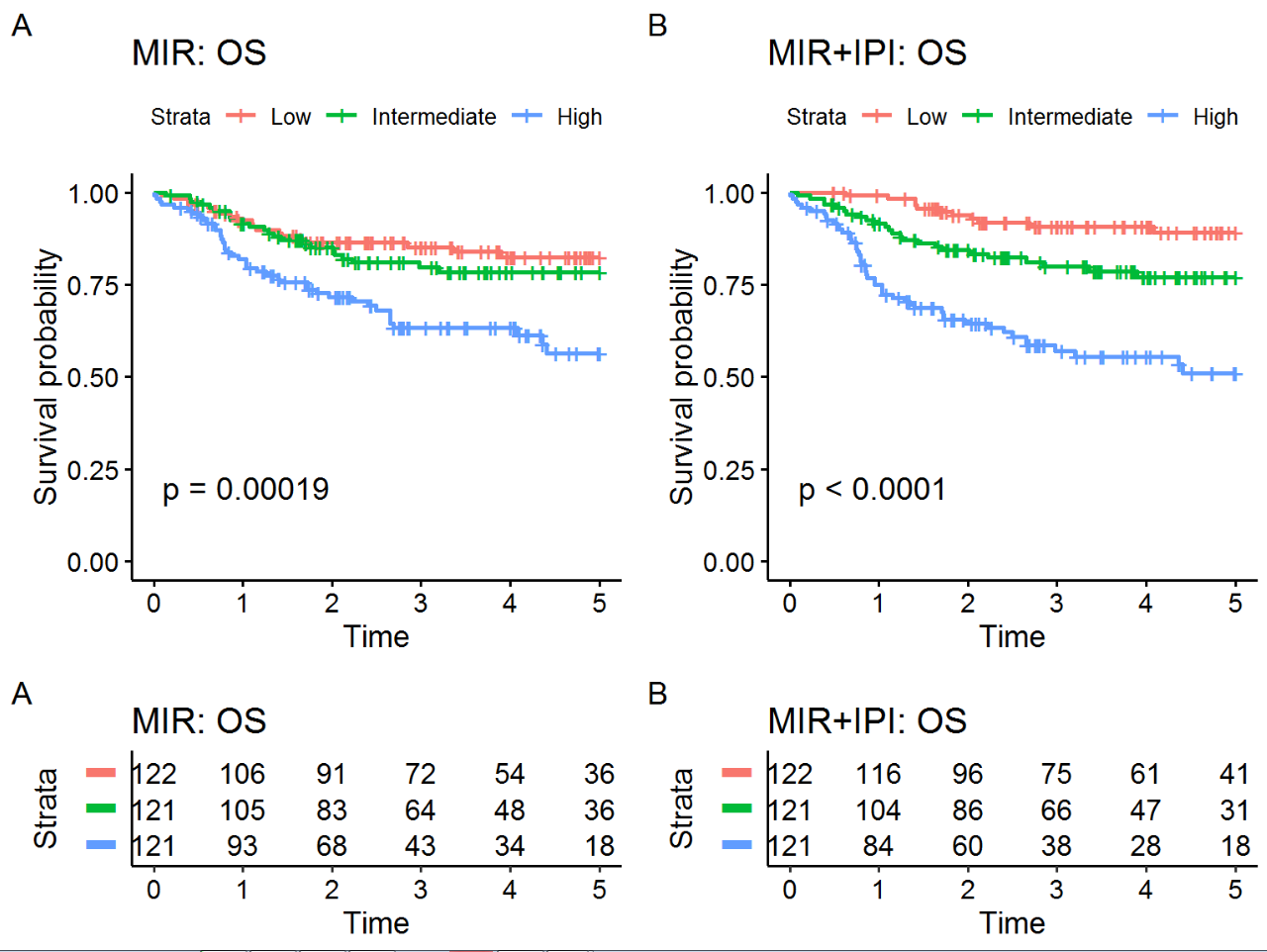


Predicted risk group vs 5-year overall survival in the combined GCB dataset. Risk scores were obtained by taking the average predicted risk score across validation folds for the repeated cross-validation and diving these into tertiles.


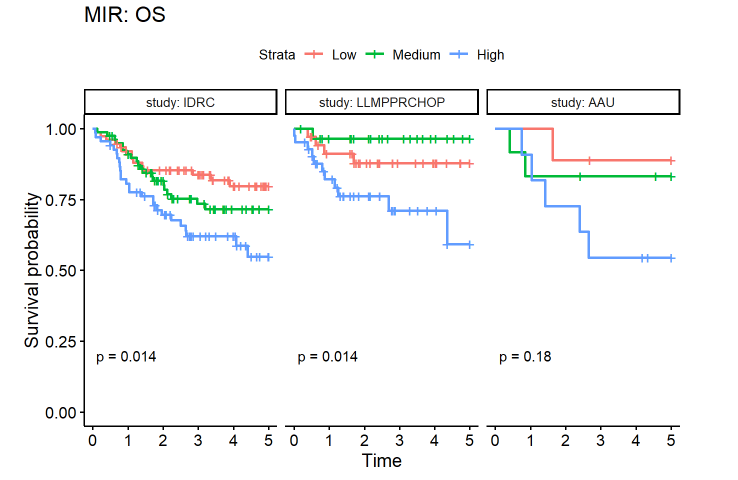
**Supplementary figure 3:**

A


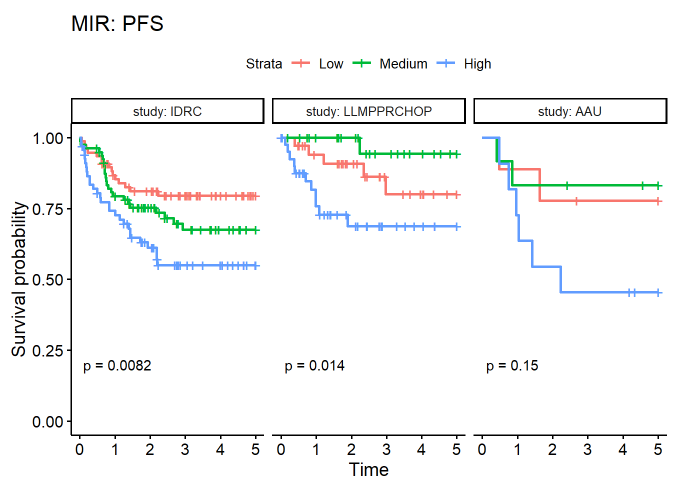


B


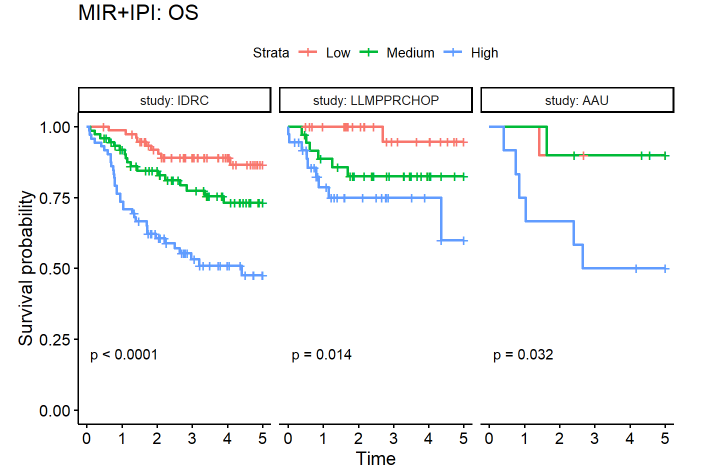
C


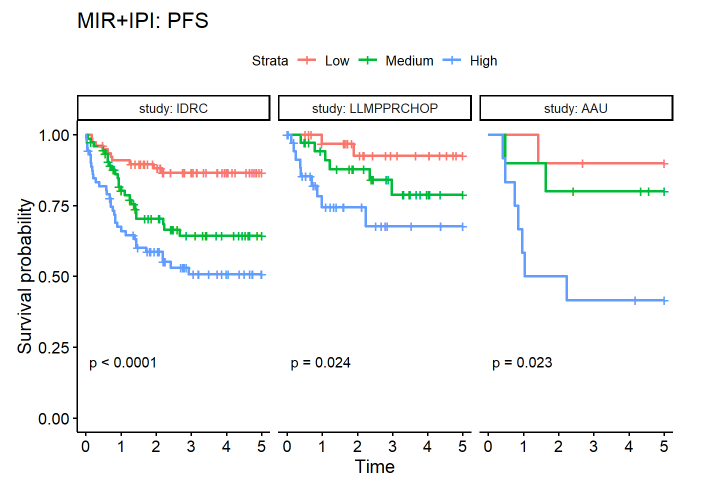


D

Predicted risk group vs 5-year overall survival in GCB classified patients within each dataset: IDRC, LLMPPR-CHOP, and AAU. Risk scores were obtained by taking the average predicted risk score across validation folds for the repeated cross-validation and diving these into tertiles.

**Supplementary figure 4:**
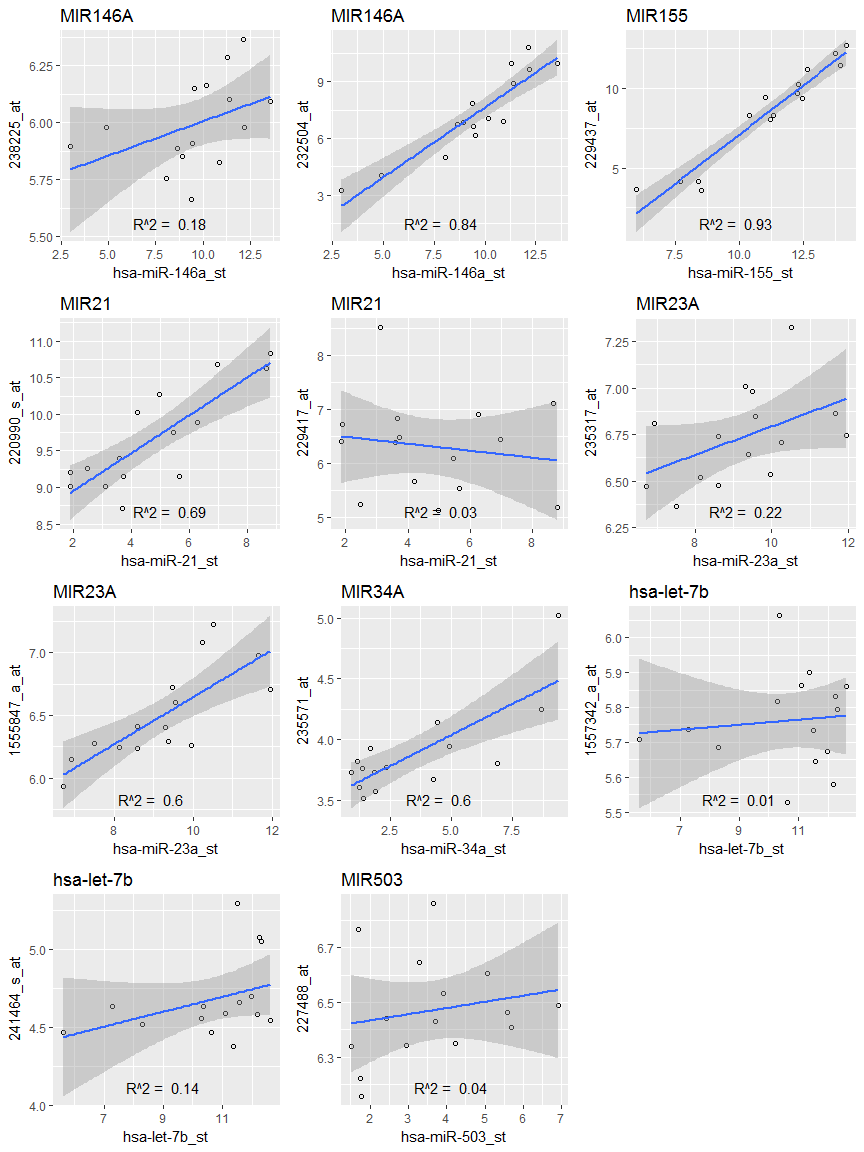


Correlation analysis between mature miRNA and miRNA encoding gene. In 15 DLBCL cell lines, the mature miRNA expression levels were measured by GeneChip miRNA 1.0.2 arrays and the precursors measured by Human Genome U133 Plus 2.0 arrays.

**Supplementary figure 5:**


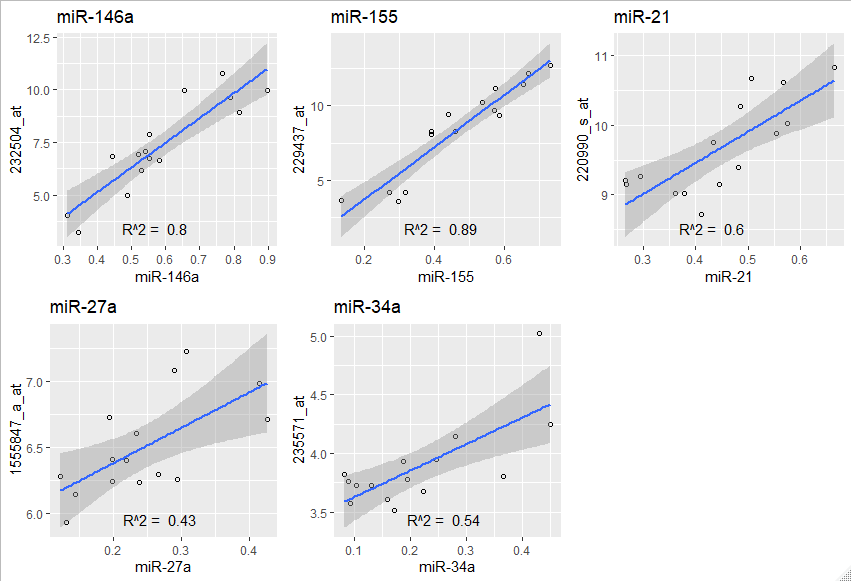


Correlation analysis between mature miRNA and miRNA encoding gene. In 15 DLBCL cell lines, the mature miRNA expression levels were measured by ddPCR and the precursors measured by Human Genome U133 Plus 2.0 arrays.
