# Supplementary material for: MicroRNAs associated to single drug components of R-CHOP identifies diffuse large B-cell lymphoma patients with poor outcome and adds prognostic value to the international prognostic index
Source: BMC Cancer. 2020 Mar 20;20:237. doi: 10.1186/s12885-020-6643-8 (PMC7082970; doi:10.1186/s12885-020-6643-8)

S2: MicroRNA associated to single drug components of R-CHOP identifies diffuse large B-cell lymphoma patients with poor outcome and adds prognostic value to the international prognostic index

Hanne Due, Rasmus Froberg Brøndum, Ken H. Young, Martin Bøgsted, and Karen Dybkær

January 6th 2020

Load Libraries

```r
library(affy)
library(limma)
library(knitr)
library(ggbiplot)
library(venn)
library(readxl)
library(tidyr)
library(sva)
library(survival)
library(survminer)
library(MASS)
library(randomForestSRC)
library(pec)
library(timeROC)
library(gridExtra)
library(VennDiagram)
library(devtools)
library(prodlim)
```

```r
session_info()
```

```
## - Session info ----------------------------------------------------------
##  setting  value
##  version  R version 3.6.1 (2019-07-05)
##  os       Windows 10 x64
##  system   x86_64, mingw32
##  ui       RTerm
##  language (EN)
##  collate  Danish_Denmark.1252
##  ctype    Danish_Denmark.1252
##  tz       Europe/Paris
##  date     2020-01-06
##
## - Packages --------------------------------------------------------------
##  ! package         * version    date       lib
##    affy            * 1.63.0     2019-05-14 [1]
##    affyio            1.55.0     2019-05-14 [1]
##    annotate          1.63.0     2019-05-14 [1]
```

```
##    AnnotationDbi      1.47.1     2019-08-20 [1]
##    assertthat         0.2.1      2019-03-21 [1]
##    backports          1.1.4      2019-04-10 [1]
##    Biobase          * 2.45.0     2019-05-14 [1]
##    BiocGenerics     * 0.31.5     2019-07-03 [1]
##    BiocManager        1.30.4     2018-11-13 [1]
##    BiocParallel     * 1.19.0     2019-05-02 [1]
##    bit                1.1-14     2018-05-29 [1]
##    bit64              0.9-7      2017-05-08 [1]
##    bitops             1.0-6      2013-08-17 [1]
##    blob               1.2.0      2019-07-09 [1]
##    broom              0.5.2      2019-04-07 [1]
##    callr              3.3.1      2019-07-18 [1]
##    cellranger         1.1.0      2016-07-27 [1]
##    cli                1.1.0      2019-03-19 [1]
##    codetools          0.2-16     2018-12-24 [1]
##    colorspace         1.4-1      2019-03-18 [1]
##    crayon             1.3.4      2017-09-16 [1]
##    data.table         1.12.2     2019-04-07 [1]
##    DBI                1.0.0      2018-05-02 [1]
##    desc               1.2.0      2018-05-01 [1]
##    devtools         * 2.1.0      2019-07-06 [1]
##    digest             0.6.20     2019-07-04 [1]
##    dplyr              0.8.3      2019-07-04 [1]
##    evaluate           0.14       2019-05-28 [1]
##    foreach            1.4.7      2019-07-27 [1]
##    formatR            1.7        2019-06-11 [1]
##    fs                 1.3.1      2019-05-06 [1]
##    futile.logger    * 1.4.3      2016-07-10 [1]
##    futile.options     1.0.1      2018-04-20 [1]
##    genefilter       * 1.67.1     2019-05-14 [1]
##    generics           0.0.2      2018-11-29 [1]
##    ggbiplot         * 0.55       2018-08-31 [1]
##    ggplot2          * 3.2.1      2019-08-10 [1]
##    ggpubr           * 0.2.3      2019-09-03 [1]
##    ggsignif           0.6.0      2019-08-08 [1]
##    glue               1.3.1      2019-03-12 [1]
##    gridExtra        * 2.3        2017-09-09 [1]
##    gtable             0.3.0      2019-03-25 [1]
##    htmltools          0.3.6      2017-04-28 [1]
##    IRanges            2.19.14    2019-08-25 [1]
##    iterators          1.0.12     2019-07-26 [1]
##    km.ci              0.5-2      2009-08-30 [1]
##    KMsurv             0.1-5      2012-12-03 [1]
##    knitr            * 1.24       2019-08-08 [1]
##    lambda.r           1.2.3      2018-05-17 [1]
##  D lattice            0.20-38    2018-11-04 [1]
##    lava               1.6.6      2019-08-01 [1]
##    lazyeval           0.2.2      2019-03-15 [1]
##    limma            * 3.41.15    2019-07-24 [1]
##    magrittr         * 1.5        2014-11-22 [1]
##    MASS             * 7.3-51.4   2019-03-31 [1]
##  D Matrix             1.2-17     2019-03-22 [1]
##    matrixStats        0.54.0     2018-07-23 [1]
```

```
##    memoise            1.1.0      2017-04-21 [1]
##  D mgcv             * 1.8-28     2019-03-21 [1]
##    munsell            0.5.0      2018-06-12 [1]
##    mvtnorm            1.0-11     2019-06-19 [1]
##    nlme             * 3.1-141    2019-08-01 [1]
##    numDeriv           2016.8-1.1 2019-06-06 [1]
##    pec              * 2018.07.26 2018-07-26 [1]
##    pillar             1.4.2      2019-06-29 [1]
##    pkgbuild           1.0.5      2019-08-26 [1]
##    pkgconfig          2.0.2      2018-08-16 [1]
##    pkgload            1.0.2      2018-10-29 [1]
##    plyr             * 1.8.4      2016-06-08 [1]
##    preprocessCore     1.47.1     2019-05-14 [1]
##    prettyunits        1.0.2      2015-07-13 [1]
##    processx           3.4.1      2019-07-18 [1]
##    prodlim          * 2018.04.18 2018-04-18 [1]
##    ps                 1.3.0      2018-12-21 [1]
##    purrr              0.3.3      2019-10-18 [1]
##    R6                 2.4.0      2019-02-14 [1]
##    randomForestSRC * 2.9.1      2019-07-08 [1]
##    Rcpp               1.0.2      2019-07-25 [1]
##    RCurl              1.95-4.12  2019-03-04 [1]
##    readxl           * 1.3.1      2019-03-13 [1]
##    remotes            2.1.0      2019-06-24 [1]
##    rlang              0.4.1      2019-10-24 [1]
##    rmarkdown          1.15       2019-08-21 [1]
##    rprojroot          1.3-2      2018-01-03 [1]
##    RSQLite            2.1.2      2019-07-24 [1]
##    S4Vectors          0.23.19    2019-08-21 [1]
##    scales           * 1.0.0      2018-08-09 [1]
##    sessioninfo        1.1.1      2018-11-05 [1]
##    stringi            1.4.3      2019-03-12 [1]
##    stringr            1.4.0      2019-02-10 [1]
##    survival         * 2.44-1.1   2019-04-01 [1]
##    survminer        * 0.4.6      2019-09-03 [1]
##    survMisc           0.5.5      2018-07-05 [1]
##    sva              * 3.33.1     2019-05-22 [1]
##    testthat           2.2.1      2019-07-25 [1]
##    tibble             2.1.3      2019-06-06 [1]
##    tidyr            * 0.8.3      2019-03-01 [1]
##    tidyselect         0.2.5      2018-10-11 [1]
##    timereg            1.9.4      2019-07-30 [1]
##    timeROC          * 0.3        2015-03-25 [1]
##    usethis          * 1.5.1      2019-07-04 [1]
##    vctrs              0.2.0      2019-07-05 [1]
##    venn             * 1.7        2018-07-31 [1]
##    VennDiagram      * 1.6.20     2018-03-28 [1]
##    withr              2.1.2      2018-03-15 [1]
##    xfun               0.9        2019-08-21 [1]
##    XML                3.98-1.20  2019-06-06 [1]
##    xtable             1.8-4      2019-04-21 [1]
##    yaml               2.2.0      2018-07-25 [1]
##    zeallot            0.1.0      2018-01-28 [1]
##    zlibbioc           1.31.0     2019-05-14 [1]
```

```
##    zoo               1.8-6      2019-05-28 [1]
##   source
##   Bioconductor
##   Bioconductor
##   Bioconductor
##   Bioconductor
##   CRAN (R 3.6.1)
##   CRAN (R 3.6.0)
##   Bioconductor
##   Bioconductor
##   CRAN (R 3.6.0)
##   Bioconductor
##   CRAN (R 3.6.0)
##   CRAN (R 3.6.0)
##   CRAN (R 3.6.0)
##   CRAN (R 3.6.1)
##   CRAN (R 3.6.0)
##   CRAN (R 3.6.1)
##   CRAN (R 3.6.0)
##   CRAN (R 3.6.1)
##   CRAN (R 3.6.0)
##   CRAN (R 3.6.1)
##   CRAN (R 3.6.1)
##   CRAN (R 3.6.1)
##   CRAN (R 3.6.0)
##   CRAN (R 3.6.1)
##   CRAN (R 3.6.1)
##   CRAN (R 3.6.1)
##   CRAN (R 3.6.1)
##   CRAN (R 3.6.1)
##   CRAN (R 3.6.1)
##   CRAN (R 3.6.0)
##   CRAN (R 3.6.1)
##   CRAN (R 3.6.0)
##   CRAN (R 3.6.0)
##   Bioconductor
##   CRAN (R 3.6.0)
##   Github (vqv/ggbiplot@7325e88)
##   CRAN (R 3.6.1)
##   CRAN (R 3.6.1)
##   CRAN (R 3.6.1)
##   CRAN (R 3.6.1)
##   CRAN (R 3.6.1)
##   CRAN (R 3.6.1)
##   CRAN (R 3.6.1)
##   Bioconductor
##   CRAN (R 3.6.1)
##   CRAN (R 3.6.0)
##   CRAN (R 3.6.0)
##   CRAN (R 3.6.1)
##   CRAN (R 3.6.0)
##   CRAN (R 3.6.0)
##   CRAN (R 3.6.1)
##   CRAN (R 3.6.1)
```

```
##   Bioconductor
##   CRAN (R 3.6.1)
##   CRAN (R 3.6.0)
##   CRAN (R 3.6.0)
##   CRAN (R 3.6.0)
##   CRAN (R 3.6.1)
##   CRAN (R 3.6.0)
##   CRAN (R 3.6.1)
##   CRAN (R 3.6.0)
##   CRAN (R 3.6.1)
##   CRAN (R 3.6.0)
##   CRAN (R 3.6.0)
##   CRAN (R 3.6.1)
##   CRAN (R 3.6.1)
##   CRAN (R 3.6.1)
##   CRAN (R 3.6.1)
##   CRAN (R 3.6.1)
##   Bioconductor
##   CRAN (R 3.6.1)
##   CRAN (R 3.6.1)
##   CRAN (R 3.6.0)
##   CRAN (R 3.6.1)
##   CRAN (R 3.6.1)
##   CRAN (R 3.6.1)
##   CRAN (R 3.6.1)
##   CRAN (R 3.6.1)
##   CRAN (R 3.6.0)
##   CRAN (R 3.6.0)
##   CRAN (R 3.6.1)
##   CRAN (R 3.6.1)
##   CRAN (R 3.6.1)
##   CRAN (R 3.6.1)
##   CRAN (R 3.6.1)
##   Bioconductor
##   CRAN (R 3.6.1)
##   CRAN (R 3.6.1)
##   CRAN (R 3.6.0)
##   CRAN (R 3.6.1)
##   CRAN (R 3.6.1)
##   CRAN (R 3.6.1)
##   CRAN (R 3.6.0)
##   Bioconductor
##   CRAN (R 3.6.1)
##   CRAN (R 3.6.1)
##   CRAN (R 3.6.1)
##   CRAN (R 3.6.1)
##   CRAN (R 3.6.1)
##   CRAN (R 3.6.0)
##   CRAN (R 3.6.1)
##   CRAN (R 3.6.1)
##   CRAN (R 3.6.0)
##   CRAN (R 3.6.0)
##   CRAN (R 3.6.1)
##   CRAN (R 3.6.1)
```

```
##  CRAN (R 3.6.0)
##  CRAN (R 3.6.0)
##  CRAN (R 3.6.0)
##  CRAN (R 3.6.1)
##  Bioconductor
##  CRAN (R 3.6.0)
##
## [1] C:/Users/y12c/Documents/R/win-library/3.6
## [2] C:/Program Files/R/R-3.6.1/library
##
##  D -- DLL MD5 mismatch, broken installation.
```

Set GGPLOT2 colors

```
#options(
#  ggplot2.continuous.colour = "viridis",
#  ggplot2.continuous.fill = "viridis"
#)
```

Custom functions

```
microarrayScale <- function(x, center = "median", scale = "sd") {
  if (class(x) == "ExpressionSet") {
    x.m <- affy::exprs(x)
  } else {
    x.m <- x
  }

  if(center == "median") {
    x.m <-  x.m - matrixStats::rowMedians(x.m,  na.rm = TRUE)
  }
  if(center == "mean") {
    x.m <-  x.m - rowMeans(x.m,  na.rm = TRUE)
  }
  if (scale == "sd") {
    x.m <- x.m / rowSds(x.m, na.rm = TRUE)
  }
  if (class(x) == "ExpressionSet") {
    Biobase::exprs(x)  <- x.m
  } else {
    x <- x.m
  }

  return(x)
}

cutFun <- function(x) {
cut(x,
breaks=c(-Inf, quantile(x, c(1/3, 2/3), na.rm = TRUE), Inf),
labels = c("Low", "Medium","High"))
}
```

Differential Expression Analysis

Load CEL files

```
cel.files <- just.rma(list.files("../ExternalData/Cell lines/CEL/All/", full.names = TRUE))
```

```
## Warning in just.rma(list.files("../ExternalData/Cell lines/CEL/All/", full.names = TRUE)): Incompati

## Warning: replacing previous import 'AnnotationDbi::tail' by 'utils::tail'
## when loading 'mirna102xgaincdf'

## Warning: replacing previous import 'AnnotationDbi::head' by 'utils::head'
## when loading 'mirna102xgaincdf'

##
```

```
cel.files
```

```
## ExpressionSet (storageMode: lockedEnvironment)
## assayData: 7815 features, 15 samples
##   element names: exprs, se.exprs
## protocolData
##   sampleNames: DB_(miRNA-1_0_2Xgain).CEL
##     FARAGE_M_(miRNA-1_0_2Xgain).CEL ...
##     U2932_M_(miRNA-1_0_2Xgain).CEL (15 total)
##   varLabels: ScanDate
##   varMetadata: labelDescription
## phenoData
##   sampleNames: DB_(miRNA-1_0_2Xgain).CEL
##     FARAGE_M_(miRNA-1_0_2Xgain).CEL ...
##     U2932_M_(miRNA-1_0_2Xgain).CEL (15 total)
##   varLabels: sample
##   varMetadata: labelDescription
## featureData: none
## experimentData: use 'experimentData(object)'
## Annotation: mirna102xgain
```

Extract expression matrix and rename samples

```
#Expression
expr <- exprs(cel.files)

#Select human miRNAs
hsa.miRNA <- expr[grep("hsa-", rownames(expr)), ]
nrow(hsa.miRNA)
```

```
## [1] 847
```

```
colnames(hsa.miRNA) <- sapply(strsplit(colnames(hsa.miRNA), "_"), function(x) x[1])
colnames(hsa.miRNA)
```

```
##  [1] "DB"       "FARAGE"   "HBL-1"    "MC-116"   "NU-DHL-1" "NU-DUL-1"
##  [7] "OCI-Ly19" "OCI-Ly3"  "OCI-Ly7"  "OCI-Ly8"  "RIVA"     "SU-DHL-4"
## [13] "SU-DHL-5" "SU-DHL-8" "U2932"
```

Load resistance classes

```
hsa.meta <- read.table("../ExternalData/Cell lines/Meta-data/Cell_line_division.csv",
                       sep = ";", header = TRUE)
kable(hsa.meta)
```

Cell_line	kit	VIN	RTX	DOX	CYC
OCI-Ly19	1	S	R	S	S
FARAGE	1	S	I	S	R
SU-DHL-5	2	S	S	S	S
MC-116	2	NA	NA	I	NA
OCI-Ly3	1	I	R	I	I
HBL-1	2	I	R	I	I
U2932	1	I	I	R	R
NU-DUL-1	2	I	S	I	S
RIVA	1	R	I	R	R
OCI-Ly7	1	R	S	R	I
SU-DHL-8	2	R	R	S	I
DB	1	R	I	R	R
SU-DHL-4	2	NA	S	R	NA
NU-DHL-1	1	NA	I	S	S
OCI-Ly8	1	NA	R	NA	NA

PCA and plot to check for batch effects

```
hsa.miRNA.pca <- prcomp(t(expr))
kit <- as.factor(hsa.meta$kit[order(hsa.meta$Cell_line)])

ggbiplot(hsa.miRNA.pca,
         obs.scale = 1,
         var.scale = 1,
         ellipse = T,
         var.axes = FALSE,
         choices = 1:2,
         groups = kit)
```

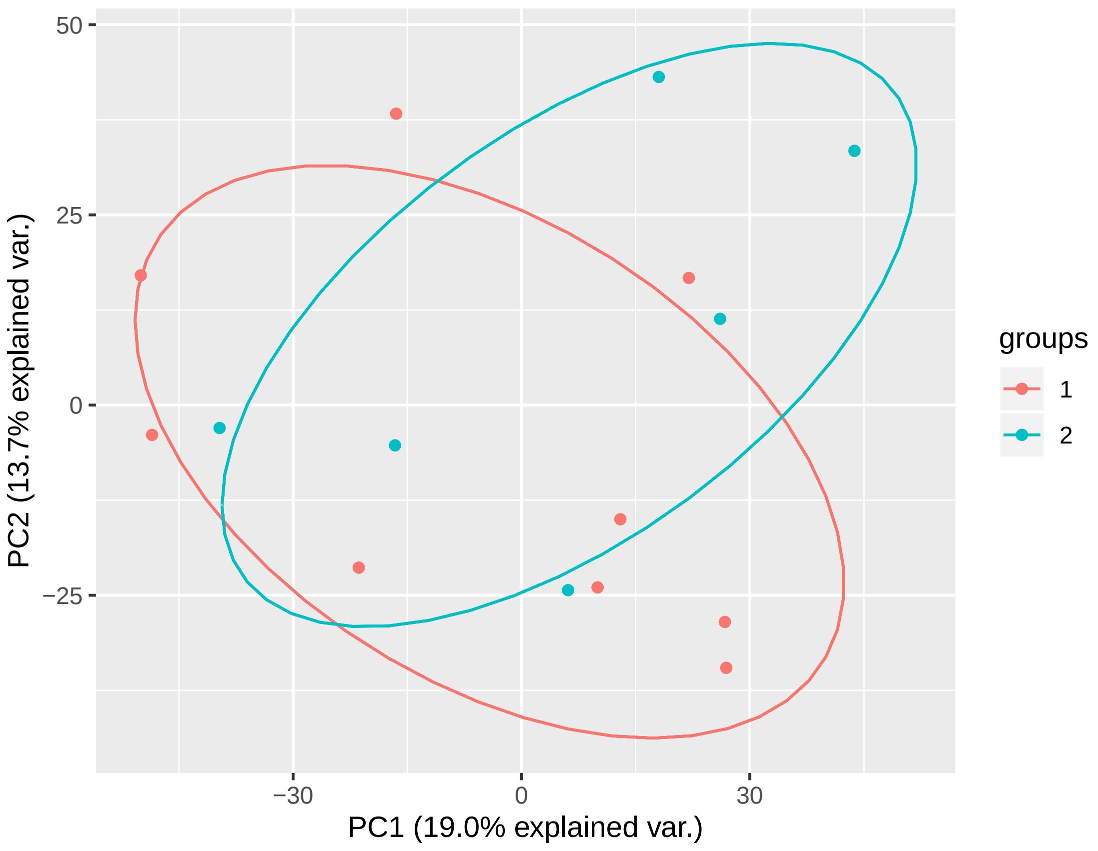

Vincristine

Extract subset for Vincristine

```
vinClass <- subset(hsa.meta, VIN %in% c("S", "R"), select = c("Cell_line", "kit", "VIN"))
vinClass
```

```
##    Cell_line kit VIN
## 1   OCI-Ly19   1   S
## 2     FARAGE   1   S
## 3   SU-DHL-5   2   S
## 9       RIVA   1   R
## 10   OCI-Ly7   1   R
## 11  SU-DHL-8   2   R
## 12        DB   1   R
```

Perform DE analysis for Vincristine Resistance

```
vinModel <- model.matrix(~ (VIN=="R")+kit, vinClass)
vinFit   <- lmFit(hsa.miRNA[, vinClass$Cell_line], vinModel)
vinFit.eb <- eBayes(vinFit)
vinResults <- topTable(vinFit.eb, coef = 2, number = 1000)
vinResults <- subset(vinResults, logFC > 2 | logFC < -2)
round(vinResults, 3)
```

```
##                      logFC AveExpr      t P.Value adj.P.Val      B
## hsa-miR-21_st       -3.217   4.197 -3.414   0.014     0.949 -4.429
```

```
## hsa-miR-21-star_st -2.282   4.204 -3.371   0.015     0.949 -4.431
## hsa-miR-155_st     -3.953  10.245 -2.566   0.043     0.949 -4.474
## hsa-miR-886-3p_st  -2.322   3.868 -2.281   0.063     0.949 -4.493
## hsa-miR-27a_st     -2.083   6.606 -1.839   0.116     0.949 -4.527
## hsa-miR-34a_st     -3.147   3.558 -1.658   0.149     0.949 -4.542
## hsa-miR-886-5p_st  -2.032   4.061 -1.627   0.155     0.949 -4.545
## hsa-let-7b_st      -2.346   9.878 -1.591   0.163     0.949 -4.548
## hsa-miR-148a_st    -2.603   4.726 -1.551   0.172     0.949 -4.551
## hsa-miR-222_st     -3.859   5.283 -1.209   0.272     0.949 -4.580
## hsa-miR-146a_st    -2.109   9.790 -1.127   0.303     0.949 -4.587
## hsa-miR-221_st     -2.819   5.788 -1.004   0.354     0.949 -4.597
## hsa-miR-708_st     -2.094   4.135 -0.976   0.367     0.949 -4.599
```

Rituximab

Extract subset for Rituximab

```
rtxClass <- subset(hsa.meta, RTX %in% c("S", "R"), select = c("Cell_line", "kit", "RTX"))
rtxClass
```

```
##    Cell_line kit RTX
## 1   OCI-Ly19   1   R
## 3   SU-DHL-5   2   S
## 5    OCI-Ly3   1   R
## 6      HBL-1   2   R
## 8   NU-DUL-1   2   S
## 10   OCI-Ly7   1   S
## 11  SU-DHL-8   2   R
## 13  SU-DHL-4   2   S
## 15   OCI-Ly8   1   R
```

Perform DE analysis for Rituximab Resistance

```
rtxModel <- model.matrix(~ (RTX=="R")+kit, rtxClass)
rtxFit   <- lmFit(hsa.miRNA[, rtxClass$Cell_line], rtxModel)
rtxFit.eb <- eBayes(rtxFit)
rtxResults <- topTable(rtxFit.eb, coef = 2, number = 1000)
rtxResults <- subset(rtxResults, logFC > 2 | logFC < -2)
round(rtxResults, 3)
```

```
##                      logFC AveExpr      t P.Value adj.P.Val      B
## hsa-miR-193b_st      -4.037   7.624 -3.915   0.004     0.995 -4.345
## hsa-miR-193b-star_st -3.765   4.193 -3.835   0.005     0.995 -4.349
## hsa-miR-151-5p_st    -2.737   9.110 -3.106   0.014     0.995 -4.398
## hsa-miR-138_st       -4.166   6.176 -3.073   0.015     0.995 -4.400
## hsa-miR-9-star_st     2.442   2.590  3.016   0.016     0.995 -4.405
## hsa-miR-183_st        2.526   5.651  3.008   0.016     0.995 -4.405
## hsa-miR-151-3p_st    -2.468   6.288 -2.635   0.029     0.995 -4.437
## hsa-miR-30a_st        2.787   4.874  2.434   0.040     0.995 -4.455
## hsa-miR-1303_st      -2.538   2.775 -2.417   0.041     0.995 -4.457
## hsa-miR-222_st        6.128   6.747  2.336   0.047     0.995 -4.464
## hsa-miR-503_st        2.518   3.586  2.300   0.049     0.995 -4.468
## hsa-miR-629-star_st  -2.252   3.931 -2.168   0.061     0.995 -4.481
## hsa-miR-27b_st        2.295   5.771  2.155   0.062     0.995 -4.482
## hsa-miR-155_st        3.043  10.724  1.965   0.084     0.995 -4.501
```

```
## hsa-miR-221_st        5.053   6.674  1.887   0.094     0.995 -4.509
## hsa-miR-424-star_st   2.293   3.464  1.858   0.099     0.995 -4.512
## hsa-miR-30a-star_st   2.039   1.707  1.795   0.109     0.995 -4.519
## hsa-miR-551b_st       2.399   2.332  1.782   0.111     0.995 -4.520
## hsa-miR-345_st        2.866   4.784  1.723   0.122     0.995 -4.526
## hsa-miR-886-3p_st     2.060   3.926  1.354   0.211     0.995 -4.564
## hsa-miR-708_st        2.108   4.108  1.156   0.280     0.995 -4.583
## hsa-miR-125b_st       2.598   4.129  1.049   0.324     0.995 -4.593
## hsa-miR-886-5p_st     2.070   4.041  0.988   0.351     0.997 -4.598
## hsa-miR-99a_st        2.121   3.714  0.905   0.391     0.997 -4.605
```

Doxorubicin

Extract subset for Doxorubicin

```
doxClass <- subset(hsa.meta, DOX %in% c("S", "R"), select = c("Cell_line", "kit", "DOX"))
doxClass
```

```
##    Cell_line kit DOX
## 1   OCI-Ly19   1   S
## 2     FARAGE   1   S
## 3   SU-DHL-5   2   S
## 7      U2932   1   R
## 9       RIVA   1   R
## 10   OCI-Ly7   1   R
## 11  SU-DHL-8   2   S
## 12        DB   1   R
## 13  SU-DHL-4   2   R
## 14  NU-DHL-1   1   S
```

Perform DE analysis for Doxorubicin Resistance

```
doxModel <- model.matrix(~ (DOX=="R")+kit, doxClass)
doxFit   <- lmFit(hsa.miRNA[, doxClass$Cell_line], doxModel)
doxFit.eb <- eBayes(doxFit)
doxResults <- topTable(doxFit.eb, coef = 2, number = 1000)
doxResults <- subset(doxResults, logFC > 2 | logFC < -2)
round(doxResults, 3)
```

```
##                          logFC AveExpr      t P.Value adj.P.Val      B
## hsa-miR-27a_st          -2.848   6.522 -3.529   0.006     0.955 -4.291
## hsa-miR-23a_st          -2.604   9.105 -3.117   0.012     0.955 -4.332
## hsa-miR-221_st          -5.511   6.382 -2.953   0.016     0.955 -4.349
## hsa-miR-222_st          -6.373   6.110 -2.868   0.018     0.955 -4.359
## hsa-miR-129-5p_st        2.009   2.524  2.751   0.022     0.955 -4.373
## hsa-miR-1295_st          2.241   2.367  2.596   0.029     0.955 -4.391
## hsa-miR-34a_st          -3.281   3.920 -2.130   0.062     0.955 -4.451
## hsa-miR-125a-5p_st       2.477   3.493  2.067   0.068     0.955 -4.460
## hsa-miR-708_st          -2.798   4.150 -1.920   0.086     0.955 -4.479
## hsa-miR-193b-star_st     2.192   4.766  1.660   0.131     0.955 -4.515
## hsa-miR-200c_st         -2.626   5.557 -1.636   0.136     0.955 -4.518
## hsa-miR-181a-2-star_st  2.467   4.282  1.526   0.161     0.955 -4.533
## hsa-miR-155_st          -2.172  10.326 -1.516   0.163     0.955 -4.534
```

Cyclophosphamide

Extract subset for Cyclophosphamide

```
cycClass <- subset(hsa.meta, CYC %in% c("S", "R"), select = c("Cell_line", "kit", "CYC"))
cycClass
```

```
##    Cell_line kit CYC
## 1   OCI-Ly19   1   S
## 2     FARAGE   1   R
## 3   SU-DHL-5   2   S
## 7      U2932   1   R
## 8   NU-DUL-1   2   S
## 9       RIVA   1   R
## 12        DB   1   R
## 14  NU-DHL-1   1   S
```

Perform DE analysis for Cyclophosphamide Resistance

```
cycModel <- model.matrix(~ (CYC=="R")+kit, cycClass)
cycFit   <- lmFit(hsa.miRNA[, cycClass$Cell_line], cycModel)
cycFit.eb <- eBayes(cycFit)
cycResults <- topTable(cycFit.eb, coef = 2, number = 1000)
cycResults <- subset(cycResults, logFC > 2 | logFC < -2)
round(cycResults, 3)
```

```
##                         logFC AveExpr      t P.Value adj.P.Val      B
## hsa-miR-486-5p_st      -4.419   4.465 -7.287   0.000     0.075  0.976
## hsa-miR-486-3p_st      -2.306   2.473 -7.081   0.000     0.075  0.853
## hsa-miR-708_st         -6.073   4.649 -6.339   0.000     0.101  0.368
## hsa-miR-30a_st          2.492   3.816  2.717   0.029     0.918 -3.300
## hsa-miR-151-3p_st      -2.236   5.757 -2.315   0.053     0.957 -3.829
## hsa-miR-664-star_st     2.152   3.392  2.160   0.067     0.957 -4.033
## hsa-miR-152_st          2.024   4.543  2.095   0.074     0.957 -4.118
## hsa-miR-99b_st          2.397   2.209  2.001   0.085     0.957 -4.240
## hsa-miR-148a-star_st    2.321   2.688  1.890   0.100     0.957 -4.383
## hsa-miR-146a_st         4.703   9.475  1.863   0.104     0.957 -4.417
## hsa-miR-193b-star_st    2.552   4.381  1.831   0.109     0.957 -4.458
## hsa-miR-221_st         -5.315   7.620 -1.829   0.109     0.957 -4.460
## hsa-miR-148a_st         2.946   5.477  1.729   0.127     0.957 -4.586
## hsa-miR-222_st         -5.986   7.443 -1.724   0.127     0.957 -4.592
## hsa-miR-138_st         -3.663   6.356 -1.667   0.138     0.957 -4.662
## hsa-miR-551b_st         2.351   2.460  1.485   0.180     0.971 -4.881
## hsa-miR-151-5p_st      -2.710   7.973 -1.351   0.218     0.984 -5.034
```

Dump DE results

```
write.csv2(vinResults, file = "../Output/vinResults.csv")
write.csv2(rtxResults, file = "../Output/rtxResults.csv")
write.csv2(doxResults, file = "../Output/doxResults.csv")
write.csv2(cycResults, file = "../Output/cycResults.csv")
```

Match MIRs to u133+2 probes

Make list with selected MIRs from DE analysis

```
vennResults <- list("Vincristine"      = rownames(vinResults),
                    "Rituximab"        = rownames(rtxResults),
                    "Doxorubicin"      = rownames(doxResults),
                    "Cyclophosphamide" = rownames(cycResults))
```

Function to rename MIRS to match u133+2 array

```
renameMir <- function(x){
  x <- sub("hsa-", "", x)
  x <- sub("_st", "", x)
  x <- sub("-", "", x)
  x <- toupper(x)
  x[x == "LET7B"] <- "hsa-let-7b"
  return(x)
}
```

Combined list of DE genes

```
comb.de.genes       <- data.frame("deMIR" = unique(unlist(vennResults)))
comb.de.genes$miRNA <- renameMir(comb.de.genes$deMIR)
comb.de.genes$miRNA
```

```
##  [1] "MIR21"           "MIR21-STAR"      "MIR155"          "MIR886-3P"
##  [5] "MIR27A"          "MIR34A"          "MIR886-5P"       "hsa-let-7b"
##  [9] "MIR148A"         "MIR222"          "MIR146A"         "MIR221"
## [13] "MIR708"          "MIR193B"         "MIR193B-STAR"    "MIR151-5P"
## [17] "MIR138"          "MIR9-STAR"       "MIR183"          "MIR151-3P"
## [21] "MIR30A"          "MIR1303"         "MIR503"          "MIR629-STAR"
## [25] "MIR27B"          "MIR424-STAR"     "MIR30A-STAR"     "MIR551B"
## [29] "MIR345"          "MIR125B"         "MIR99A"          "MIR23A"
## [33] "MIR129-5P"       "MIR1295"         "MIR125A-5P"      "MIR200C"
## [37] "MIR181A-2-STAR" "MIR486-5P"       "MIR486-3P"       "MIR664-STAR"
## [41] "MIR152"          "MIR99B"          "MIR148A-STAR"
```

Match MIRs to u133+2 probes

```
MIRu133 <- read_xlsx("../ExternalData/Cell lines/Meta-data/miRNAs_U133.xlsx", sheet = 1)
MIRu133 <- separate(MIRu133, "miRNA", sep =" /// ", into=letters[1:7])
```

```
## Warning: Expected 7 pieces. Missing pieces filled with `NA` in 298 rows [1,
## 2, 3, 4, 5, 6, 7, 8, 9, 10, 11, 12, 13, 14, 15, 16, 17, 18, 19, 20, ...].
```

```
comb.de.genes.u133 <- merge(comb.de.genes, MIRu133, by.x = "miRNA", by.y = "a")
comb.de.genes.u133 <- comb.de.genes.u133[, c("miRNA", "deMIR", "Probe")]
for(column in letters[2:7]){
  temp <- merge(comb.de.genes, MIRu133, by.x = "miRNA", by.y = column)
  comb.de.genes.u133 <- rbind(comb.de.genes.u133, temp[, c("miRNA", "deMIR", "Probe")])
}
probes <- comb.de.genes.u133$Probe
names(probes) <- comb.de.genes.u133$miRNA
probes
```

```
##       MIR146A       MIR146A        MIR155         MIR21         MIR21
##    "238225_at"   "232504_at"   "229437_at"  "220990_s_at"   "229417_at"
```

```
##         MIR23A         MIR23A         MIR34A     hsa-let-7b     hsa-let-7b
##    "235317_at" "1555847_a_at"    "235571_at" "1557342_a_at"  "241464_s_at"
##         MIR503
##    "227488_at"
```

Match selected probes to lists from drugs too see how many of the selected probes correspond to DE Mirs in specific drugs

```r
vennResults2 <- lapply(vennResults, renameMir)
probeInDrug <- function(x){
  DrugProbes    <- probes[names(probes) %in% x]
  numProbes     <- length(DrugProbes)
  return(list("nProbes" = numProbes,
              "probes"  = DrugProbes)
  )
}
drugProbes <- lapply(vennResults2, probeInDrug)
drugProbes
```

```
## $Vincristine
## $Vincristine$nProbes
## [1] 8
##
## $Vincristine$probes
##        MIR146A        MIR146A         MIR155          MIR21          MIR21
##    "238225_at"    "232504_at"    "229437_at" "220990_s_at"    "229417_at"
##         MIR34A     hsa-let-7b     hsa-let-7b
##    "235571_at" "1557342_a_at"  "241464_s_at"
##
##
## $Rituximab
## $Rituximab$nProbes
## [1] 2
##
## $Rituximab$probes
##      MIR155      MIR503
## "229437_at" "227488_at"
##
##
## $Doxorubicin
## $Doxorubicin$nProbes
## [1] 4
##
## $Doxorubicin$probes
##         MIR155         MIR23A         MIR23A         MIR34A
##    "229437_at"    "235317_at" "1555847_a_at"    "235571_at"
##
##
## $Cyclophosphamide
## $Cyclophosphamide$nProbes
## [1] 2
##
## $Cyclophosphamide$probes
##     MIR146A     MIR146A
## "238225_at" "232504_at"
```

Construct Venn-diagram with selected MIRS and probes

```
v1 <- venn.diagram(vennResults, filename = NULL, fill = 1:4)
v2 <- venn.diagram(lapply(drugProbes, function(x) x$probes), filename = NULL)

# Combine Labels
for(i in 9:23){
  if(v1[[i]]$label != "0"){
     v1[[i]]$label = paste(v1[[i]]$label, "(", v2[[i]]$label, ")")
  }
}

grid.newpage()
grid.draw(v1)
```

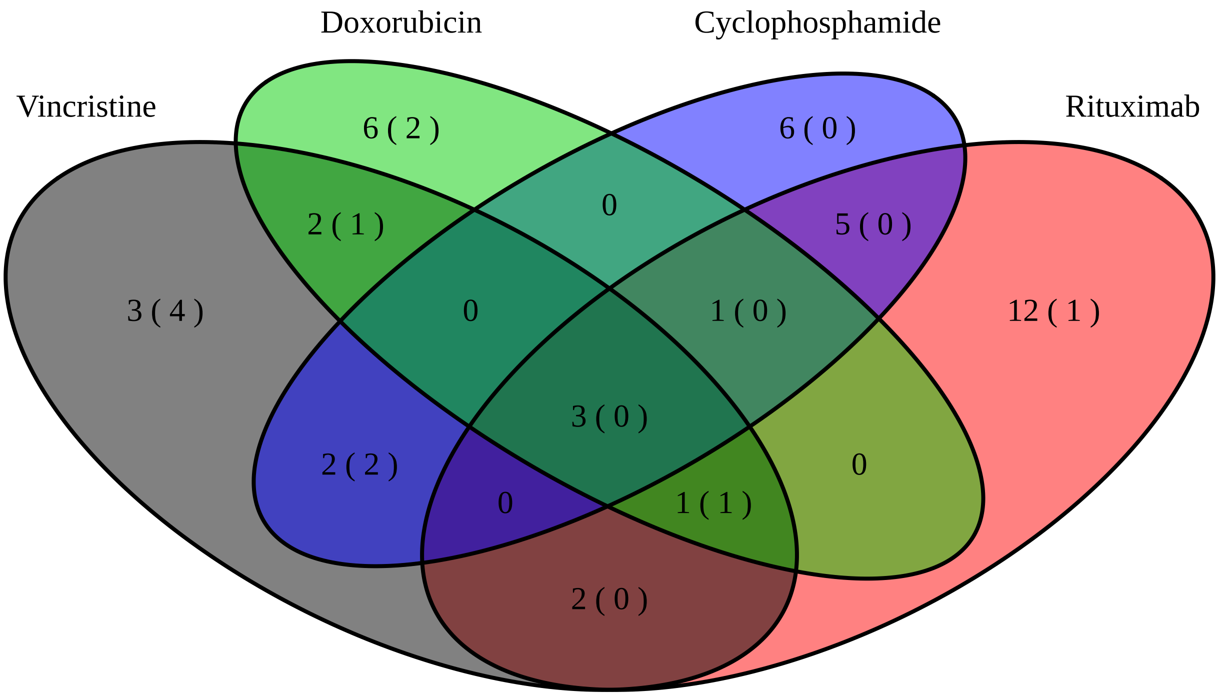

Check correlation of MIR and U133+2 array

load U133+2 data for cell-lines and adjust column names

```
bCellu133 <- just.rma(list.files("../ExternalData/Cell lines/CEL U133", full.names = T))
```

```
## Warning in just.rma(list.files("../ExternalData/Cell lines/CEL U133", full.names = T)): Incompatible

## Warning: replacing previous import 'AnnotationDbi::tail' by 'utils::tail'
## when loading 'hgu133plus2cdf'

## Warning: replacing previous import 'AnnotationDbi::head' by 'utils::head'
```

```
## when loading 'hgu133plus2cdf'

##

##
## Attaching package: 'hgu133plus2cdf'

## The following objects are masked from 'package:mirna102xgaincdf':
##
##     i2xy, xy2i
```

```
tempNames <- sapply(strsplit(colnames(bCellu133), "_"), function(x) x[3])
tempNames <- sub(".CEL", "", tempNames)
tempNames[15] <- substr(colnames(bCellu133)[15],1,8)
colnames(bCellu133) <- tempNames
bCellu133 <- bCellu133[, order(colnames(bCellu133))]
bCellu133 <- exprs(bCellu133)
```

Match columns in MIR and u133+2 datasets

```
table(colnames(bCellu133) == colnames(hsa.miRNA))
```

```
##
## TRUE
##   15
```

```
p <- list()
for(i in 1:nrow(comb.de.genes.u133)){
  mirName <- as.character(comb.de.genes.u133[i,2])
  u133Name <- as.character(comb.de.genes.u133[i,3])
  regression  <- lm(bCellu133[u133Name,] ~  hsa.miRNA[mirName,])

  tempPlot <- data.frame("mir" = hsa.miRNA[mirName,],
                         "u133" = bCellu133[u133Name,])

  p[[i]] <- ggplot(tempPlot, aes(x=mir, y=u133)) +
    geom_point(shape=1) +
    geom_smooth(method=lm) +
    xlab(mirName) +
    ylab(u133Name) +
    ggtitle(comb.de.genes.u133[i,1]) +
    annotate("text", x = -Inf, y = -Inf, hjust = -1, vjust = -1,
             label = paste("R^2 = ", round(summary(regression)$r.squared, 2)))

 }

grid.arrange(grobs = p, ncol = 3)
```

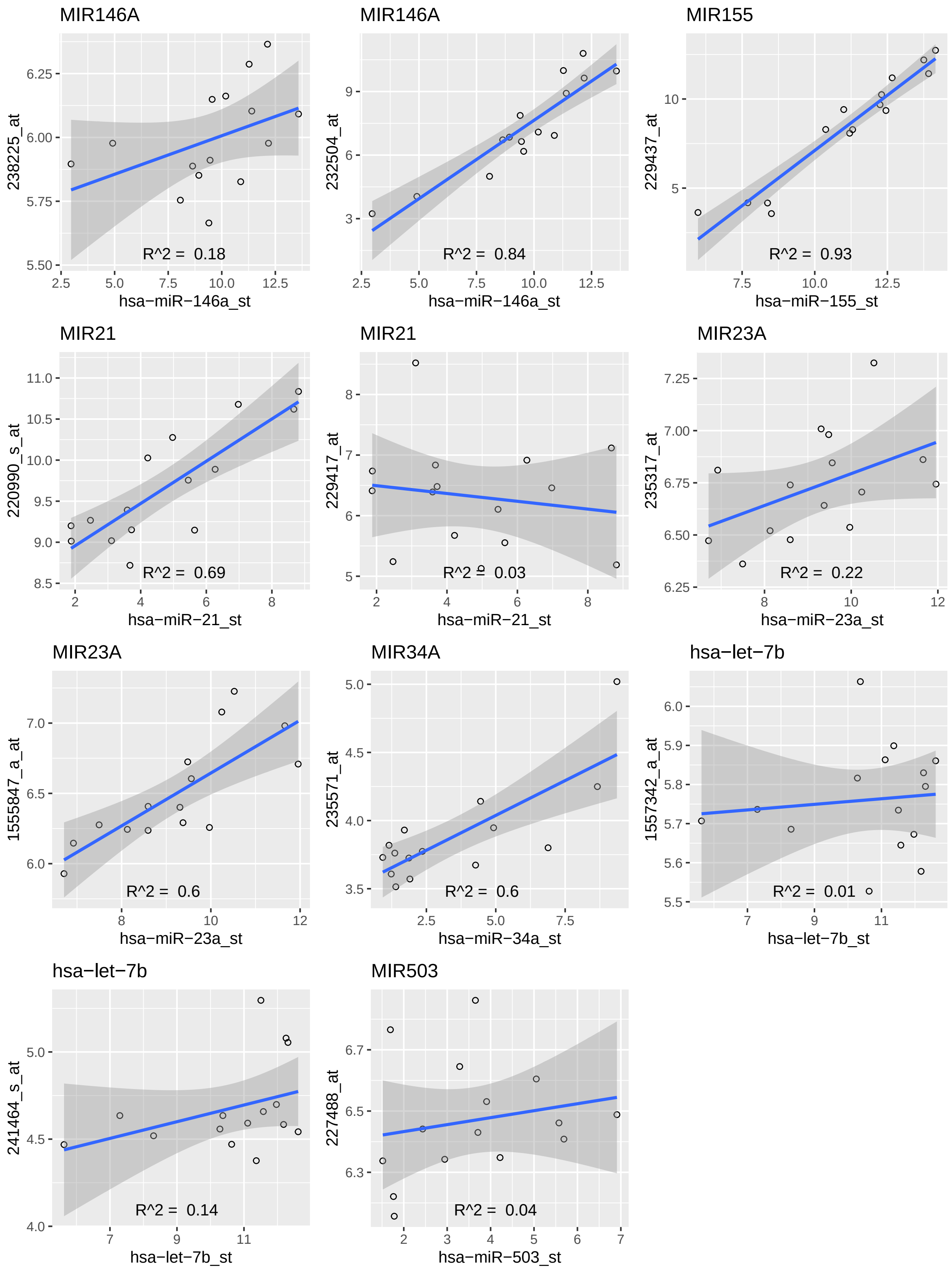
MIR146A
238225_at
hsa−miR−146a_st
R^2 = 0.18
MIR146A
232504_at
hsa−miR−146a_st
R^2 = 0.84
MIR155
229437_at
hsa−miR−155_st
R^2 = 0.93
MIR21
220990_s_at
hsa−miR−21_st
R^2 = 0.69
MIR21
229417_at
hsa−miR−21_st
R^2 = 0.03
MIR23A
235317_at
hsa−miR−23a_st
R^2 = 0.22
MIR23A
1555847_a_at
hsa−miR−23a_st
R^2 = 0.6
MIR34A
235571_at
hsa−miR−34a_st
R^2 = 0.6
hsa−let−7b
1557342_a_at
hsa−let−7b_st
R^2 = 0.01
hsa−let−7b
241464_s_at
hsa−let−7b_st
R^2 = 0.14
MIR503
227488_at
hsa−miR−503_st
R^2 = 0.04

Clinical Data

```
combined.data.combat.file <- "../GeneratedData/combined.data.combat.RData"
```

Load and pre-process GEP Data

Load pre-normalized u133+2 datasets

```
load("../ExternalData/Clinical data/IDRC_cohort/CelFiles/pre-processed/GEPIDRC_RMA_affy.RData")
load("../ExternalData/Clinical data/LLMPP_cohort/CelFiles/pre-processed/GEPLLMPPRCHOP_RMA_affy.RData")
```

Read and normalize new data

```
AAUGEP.file <- "../GeneratedData/AAUGEP.file.Rdata"
if(!file.exists(AAUGEP.file)){
  AAU <- just.rma(list.files("../ExternalData/Clinical data/AAU_cohort/CelFiles/All/",
                             full.names = TRUE))
  GEPAAU <- exprs(AAU)
  save(GEPAAU, file = AAUGEP.file)
} else{
  load(AAUGEP.file)
}
```

Combine data and remove probes with missing data

```
if(!file.exists(combined.data.combat.file)){
  combined.data <- cbind(exprs(GEPIDRC), exprs(GEPLLMPPRCHOP), GEPAAU)
  combined.data <- combined.data[rowSums(is.na(combined.data))==0, ]
}
```

PCA plot to check for batch effects

```
combined.data.pca.file <- "../GeneratedData/combined.data.pca.Rdata"

if(!file.exists(combined.data.pca.file)){
  combineddata.pca <- prcomp(t(combined.data))
  save(combineddata.pca, file = combined.data.pca.file)
} else{
  load(combined.data.pca.file)
}
batch <- c(rep("IDRC", ncol(GEPIDRC)),
           rep("LLMPPRCHOP", ncol(GEPLLMPPRCHOP)),
           rep("AAU", ncol(GEPAAU)))

ggbiplot(combineddata.pca,
         obs.scale = 1,
         var.scale = 1,
         ellipse = T,
         var.axes = FALSE,
         choices = 1:2,
         groups = batch
         )
```

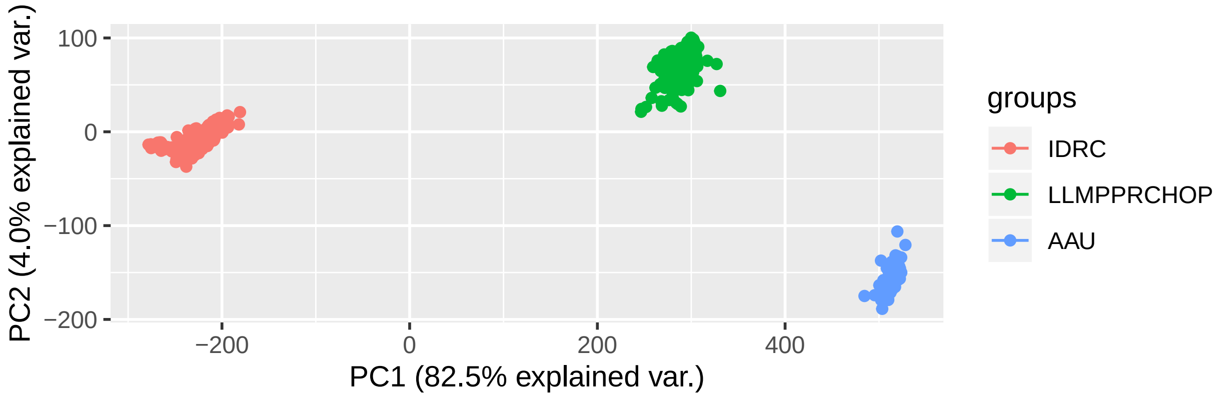

COMBAT adjust data

```
combined.data.combat.file <- "../GeneratedData/combined.data.combat.RData"
if(!file.exists(combined.data.combat.file)){
  combined.data.combat <- ComBat(combined.data,
                                 batch = batch)
  save(combined.data.combat, file = combined.data.combat.file)
} else{
  load(combined.data.combat.file)
}

colnames(combined.data.combat) <- sub(".CEL", "", colnames(combined.data.combat))
colnames(combined.data.combat) <- sub(".cel", "", colnames(combined.data.combat))
colnames(combined.data.combat) <- sub("_\\(HG-U133_Plus_2\\)", "", colnames(combined.data.combat))
```

Check for batch effect after removal

```
combined.data.combat.pca.file <- "../GeneratedData/combined.data.combat.pca.RData"
if(!file.exists(combined.data.combat.pca.file)){
  combineddata.combat.pca <- prcomp(t(combined.data.combat))
  save(combineddata.combat.pca, file = combined.data.combat.pca.file)
} else{
  load(combined.data.combat.pca.file)
}

ggbiplot(combineddata.combat.pca,
         obs.scale = 1,
```

```r
        var.scale = 1,
        ellipse = T,
        var.axes = FALSE,
        choices = 1:2,
        groups = batch
        )
```

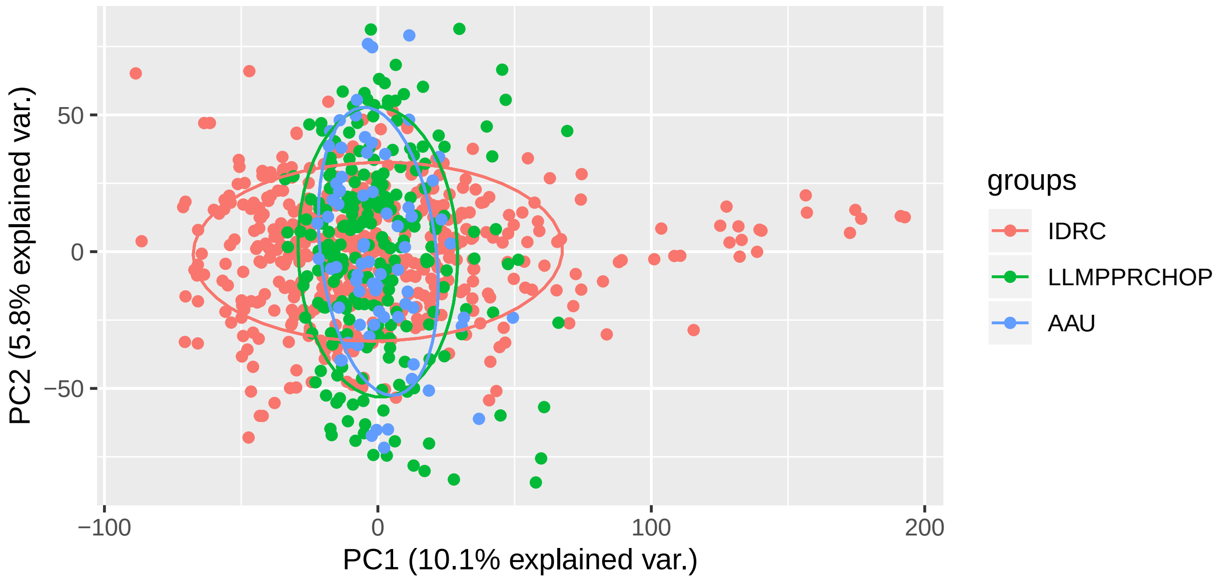

Combine metadata

Load raw data

```r
load("../ExternalData/Clinical data/IDRC_cohort/Metadata/metadataIDRC.RData")
load("../ExternalData/Clinical data/LLMPP_cohort/Metadata/metadataLLMPPRCHOP.RData")
load("../ExternalData/Clinical data/AAU_cohort/Metadata/metadata_AAUcohort_RMA_BC_affy.RData")
metadataAAU <- tmp1
rm(tmp1)
```

Extract columns from IDRC

```r
IDRC.tmp <- subset(metadataIDRC, select = c("Array.Data.File",
                                            "IPI.score",
                                            "WrightClass2",
                                            "OS",
                                            "PFS",
                                            "age",
                                            "Response",
```

```
                                                 "Gender"))
IDRC.tmp$batch <- rep("IDRC", nrow(IDRC.tmp))
names(IDRC.tmp) <- c("ID", "IPI", "ABCGCB", "OS", "PFS", "age","Response", "Gender", "study")

IDRC.tmp$IPI[IDRC.tmp$IPI == "UNK"] <- NA
```

Extract columns from LLMPPRCHOP

```
LLMPRCHOP.tmp <- subset(metadataLLMPPRCHOP, select = c("GEO.ID",
                                                        "ipi",
                                                        "WrightClass2",
                                                        "OS",
                                                        "PFS",
                                                        "age",
                                                        "Response.to.Initial.Therapy",
                                                        "Gender"))
LLMPRCHOP.tmp$batch <- rep("LLMPPRCHOP", nrow(LLMPRCHOP.tmp))
names(LLMPRCHOP.tmp) <- c("ID", "IPI", "ABCGCB", "OS", "PFS","age", "Response", "Gender","study")
LLMPRCHOP.tmp$PFS[is.na(LLMPRCHOP.tmp$PFS)] <- LLMPRCHOP.tmp$OS[is.na(LLMPRCHOP.tmp$PFS)]


## Recode response
LLMPRCHOP.tmp$Response <- as.character(LLMPRCHOP.tmp$Response)
LLMPRCHOP.tmp$Response[LLMPRCHOP.tmp$Response == ""]            <- NA
LLMPRCHOP.tmp$Response[LLMPRCHOP.tmp$Response == "complete"]    <- "CR"
LLMPRCHOP.tmp$Response[LLMPRCHOP.tmp$Response == "partial"]     <- "PR"
LLMPRCHOP.tmp$Response[LLMPRCHOP.tmp$Response == "progression"] <- "PD"
LLMPRCHOP.tmp$Response[LLMPRCHOP.tmp$Response == "stable"]      <- "SD"
```

Extract columns from AAU

```
metadataAAU$OS <- with(metadataAAU, Surv(FU, OS.censor))
metadataAAU$PFS <- with(metadataAAU, Surv(PFS, PFScensor))
AAU.tmp <- subset(metadataAAU, select = c("H.nummer",
                                          "IPI",
                                          "ABC.GCB",
                                          "OS",
                                          "PFS",
                                          "Alder"))

AAU.tmp$ABC.GCB <- as.character(AAU.tmp$ABC.GCB)
AAU.tmp$ABC.GCB[AAU.tmp$ABC.GCB == "NC"] <- "UC"
AAU.tmp$Response <- rep(NA, nrow(AAU.tmp))
AAU.tmp$Gender <- ifelse(metadataAAU$Gender == "Kvinde", "F", "M")
AAU.tmp$batch <- rep("AAU", nrow(AAU.tmp))
names(AAU.tmp) <- c("ID", "IPI", "ABCGCB", "OS", "PFS","age", "Response", "Gender","study")
```

Combine data

```
combined.metadata <- rbind(IDRC.tmp, LLMPRCHOP.tmp, AAU.tmp)
combined.metadata <- droplevels.data.frame(combined.metadata)
combined.metadata$ID <- sub(".CEL", "", combined.metadata$ID)
combined.metadata$OS <- Surv(combined.metadata$OS[,1], combined.metadata$OS[,2])
combined.metadata$PFS <- Surv(combined.metadata$PFS[,1], combined.metadata$PFS[,2])
```

Unwrap OS and PFS variables. This is needed for calcualtion of the Brier score and time varying AUC.

```r
combined.metadata$OStime   <- combined.metadata$OS[,1]
combined.metadata$OSstatus <- combined.metadata$OS[,2]

combined.metadata$PFStime   <- combined.metadata$PFS[,1]
combined.metadata$PFSstatus <- combined.metadata$PFS[,2]

# 5 year OS / PFS
time5   <- pmin(combined.metadata$OStime, 5)
status5 <- as.numeric(time5 < 5 & combined.metadata$OSstatus == 1)
combined.metadata$OS5 <- Surv(time = time5, event = status5)

time5   <- pmin(combined.metadata$PFStime, 5)
status5 <- as.numeric(time5 < 5 & combined.metadata$PFSstatus == 1)
combined.metadata$PFS5 <- Surv(time = time5, event = status5)
```

Plot survival in different studies included in the combined data

```r
pfs.survfit <- survfit(PFS5~study, data = combined.metadata)
names(pfs.survfit$strata) <- sub("study=", "", names(pfs.survfit$strata))
pfs.study.plot <- ggsurvplot(pfs.survfit,
           data = combined.metadata,
           title = "PFS5 by study",
           pval = TRUE)

os.survfit <- survfit(OS5~study, data = combined.metadata)
names(os.survfit$strata) <- sub("study=", "", names(os.survfit$strata))
os.study.plot <- ggsurvplot(os.survfit,
           data = combined.metadata,
           title = "OS5 by study",
           pval = TRUE)

arrange_ggsurvplots(list(pfs.study.plot, os.study.plot))
```

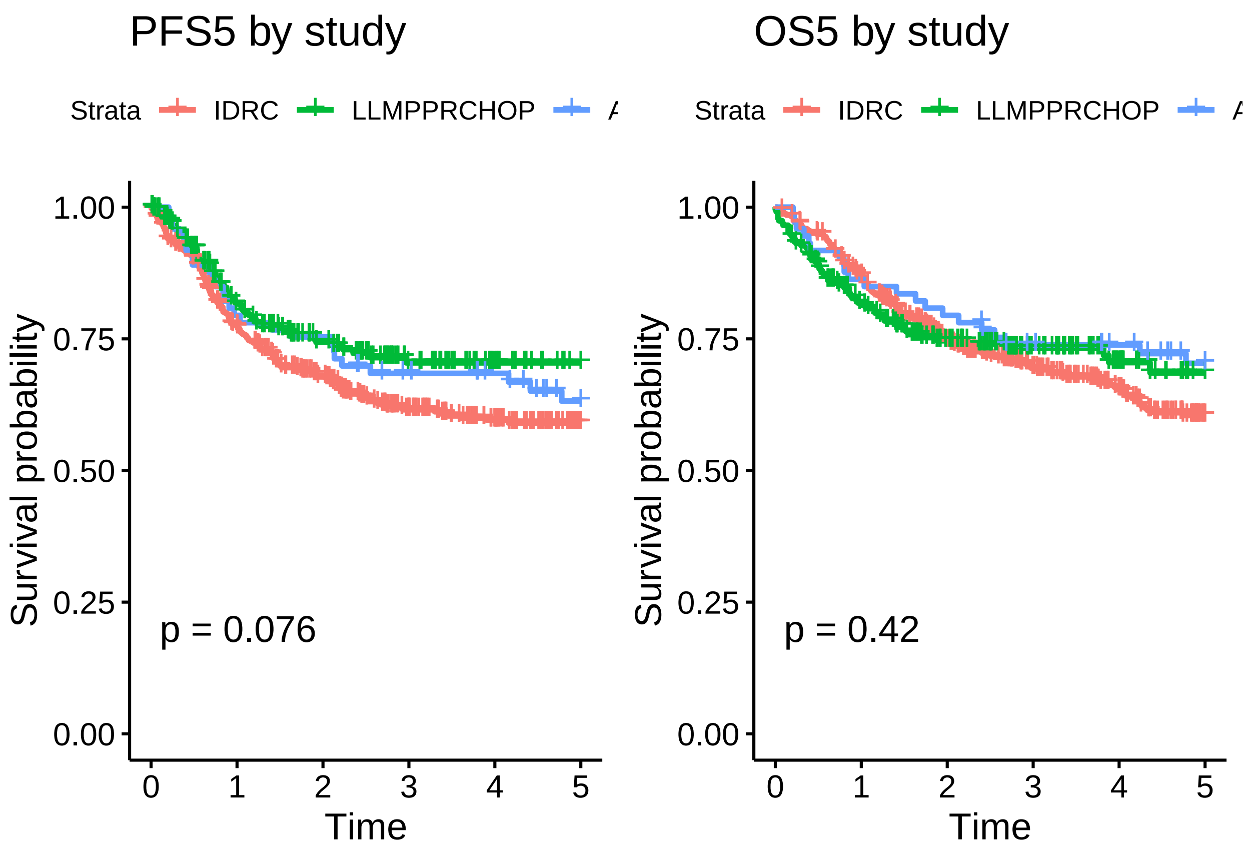

Get 5-year OS / PFS times

```
pfs.sum <- summary(pfs.survfit, times = 5)
os.sum <- summary(os.survfit, times = 5)
pfs.sum
```

```
## Call: survfit(formula = PFS5 ~ study, data = combined.metadata)
##
##                 IDRC
##          time        n.risk       n.event      survival       std.err
##         5.000        81.000       172.000         0.590         0.025
## lower 95% CI upper 95% CI
##         0.543         0.641
##
##                 LLMPPRCHOP
##          time        n.risk       n.event      survival       std.err
##        5.0000       22.0000       56.0000        0.7045        0.0343
## lower 95% CI upper 95% CI
##        0.6403        0.7751
##
##                 AAU
##          time        n.risk       n.event      survival       std.err
##        5.0000       33.0000       26.0000        0.6318        0.0582
## lower 95% CI upper 95% CI
##        0.5275        0.7568
```

```r
os.sum
```

```
## Call: survfit(formula = OS5 ~ study, data = combined.metadata)
##
##                 IDRC
##         time       n.risk      n.event     survival      std.err
##       5.0000      98.0000     155.0000       0.6044       0.0266
## lower 95% CI upper 95% CI
##       0.5544       0.6589
##
##                 LLMPPRCHOP
##         time       n.risk      n.event     survival      std.err
##       5.0000      24.0000      60.0000       0.6854       0.0393
## lower 95% CI upper 95% CI
##       0.6125       0.7670
##
##                 AAU
##         time       n.risk      n.event     survival      std.err
##       5.0000      38.0000      21.0000       0.7036       0.0548
## lower 95% CI upper 95% CI
##       0.6040       0.8195
```

Create metadata summary table

```r
# Survival summary
table1.pfs <- paste0(round(pfs.sum$surv, 2), " (", round(pfs.sum$lower, 2), ";", round(pfs.sum$upper, 2
table1.os <- paste0(round(os.sum$surv, 2), " (", round(os.sum$lower, 2), ";", round(os.sum$upper, 2), "


metaSummary <- rbind(table(combined.metadata$study),
t(table(combined.metadata$study, combined.metadata$Gender)),
tapply(combined.metadata$age, combined.metadata$study, median),
tapply(combined.metadata$age, combined.metadata$study,
       function(x) paste(range(x), collapse = "-")),
t(table(combined.metadata$study, as.numeric(as.character(combined.metadata$IPI)) < 2)),
t(tapply(combined.metadata$IPI, combined.metadata$study, function(x) sum(is.na(x)))),
t(table(combined.metadata$study, combined.metadata$ABCGCB)),
t(table1.pfs),
t(table1.os))
row.names(metaSummary) <- c("N", "Female", "Male", "Median age",
                            "Range age", "IPI 0-1", "IPI 2-5",
                            "IPI NA", "ABC", "GCB", "UC", "5-year PFS", "5-year OS")
kable(metaSummary)
```

	IDRC	LLMPPRCHOP	AAU
N	468	233	73
Female	198	99	30
Male	270	134	43
Median age	63	61	66
Range age	18-92	17-92	20-87
IPI 0-1	254	94	48
IPI 2-5	168	70	21
IPI NA	46	69	4
ABC	199	93	32
GCB	225	107	32

	IDRC	LLMPPRCHOP	AAU
UC	44	33	9
5-year PFS	0.59 (0.54;0.64)	0.7 (0.64;0.78)	0.63 (0.53;0.76)
5-year OS	0.6 (0.55;0.66)	0.69 (0.61;0.77)	0.7 (0.6;0.82)

Check if metadata match GEP data

```
table(colnames(combined.data.combat) %in% combined.metadata$ID)
```

```
##
## FALSE  TRUE
##     2   774
```

Two GEP files have no data, so they are removed

```
combined.data.combat <- combined.data.combat[, colnames(combined.data.combat) %in%
                                               combined.metadata$ID]
```

Function to do uni- and multivariate cox regressions and summarize results

```
uni.multi.cox <- function(cox.data, probes, outcome = "OS"){
  ## Univariate Cox for each probe
  uni.cox.results <- list()
  for(i in 1:length(probes)){
    uni.cox.formula <- formula(paste0(outcome,"~","study+", "`",probes[i],"`"))
    uni.cox.results[[probes[i]]] <- summary(coxph(uni.cox.formula, data = cox.data))
  }

  ## Summarize univariante results
  uni.coef <- round(sapply(uni.cox.results, function(x) x$coefficients[3,2]),2 )
  uni.conf <- round(t(sapply(uni.cox.results, function(x) x$conf.int[3,3:4])),2)
  uni.conf <- paste0("(", uni.conf[,1], ";", uni.conf[,2],")")
  uni.p    <- round(sapply(uni.cox.results, function(x) x$coefficients[3,5]),3)

  ## Multivariate Cox
  multi.cox.formula <- formula(paste0(outcome,"~","study+","`",
                                      paste(probes, collapse="`+`"),"`"))
  multi.cox  <- summary(coxph(multi.cox.formula, data = cox.data))

  ## Summarize multivariate results
  multi.coef <- round(multi.cox$coefficients[-c(1,2),2], 2)
  multi.conf <- round(multi.cox$conf.int[-c(1,2),3:4], 2)
  multi.conf <- paste0("(", multi.conf[,1], ";", multi.conf[,2],")")
  multi.p    <- round(multi.cox$coefficients[-c(1,2),5], 3)

  combined.results <- data.frame("MIR" = names(probes),
                                 "uni.HR"    = uni.coef,
                                 "uni.conf" = uni.conf,
                                 "uni.p"     = uni.p,
                                 "multi.HR" = multi.conf,
                                 "multi.conf" = multi.conf,
                                 "multi.p"     = multi.p)

  return(list("uni.cox.results"   = uni.cox.results,
              "multi.cox.results" = multi.cox,
```

```
                  "combined.results"   = combined.results))
}
```

Analysis with Cross-validation

```
timeVaryingAUC <- function(survivalPrediction, timeVar, delta, evalTimes){
      timeAUC <- rep(NA, length(evalTimes))
      for(i in 1:length(evalTimes)){
        timeAUC[i] <- timeROC(T = timeVar,
                              marker = - survivalPrediction[, i],
                              delta = delta,
                              times = evalTimes[i],
                              cause = 1)$AUC[2]
      }
      timeAUC
    }
```

Function to plot a list of timeVarying AUCs

```
plottAUC <- function(x, times.interest = times.interest){
  nvar <- length(x)
  xx <- data.frame("time" = rep(times.interest, nvar),
                   "tAUC" = unlist(x),
                   "Model" = rep(names(x), each = length(times.interest)))
  ggplot(xx, aes(x = time, y = tAUC, col = Model)) +
    geom_line() +
    ylab("tAUC") +
    ylim(c(0.4,0.75))
}

plottAUCList <- function(x, times.interest = times.interest){
  nvar <- length(x[[1]])
  xx <- list()
  for(i in 1:length(x)){
    xx[[i]] <- data.frame("time" = rep(times.interest, nvar),
                     "tAUC" = unlist(x[[i]]),
                     "Model" = rep(names(x[[i]]), each = length(times.interest)))
  }
  p <- ggplot(xx[[i]], aes(x = time, y = tAUC, col = Model)) +
    geom_line() +
    ylab("tAUC") +
    ylim(c(0.4,0.8))

  for(i in 2:length(xx)){
    p <- p + geom_line(data = xx[[i]])
  }
  return(p)
}

plottAUCListMean <- function(x, times.interest = times.interest){
  models <- names(x[[1]])
  plotData <- data.frame("Model" = NULL,"time" =NULL, "Mean" = NULL, "SD" = NULL)
```

```
  for(m in models){
    y <- sapply(x, function(x) x[[m]])
    means <- rowMeans(y)
    sds   <- rowSds(y)
    temp <- data.frame("Model" = rep(m, length(means)),
                       "time" = times.interest,
                       "Mean" = means,
                       "SD" = sds)
    plotData = rbind(plotData, temp)
  }

  ggplot(plotData, aes(x = time, y = Mean, col = Model)) +
    geom_line(size  = 1.5) +
    ylab("tAUC") +
    ylim(c(0.4,0.8)) +
    geom_ribbon(aes(x = time, ymin = (Mean -2*SD),
                    ymax = (Mean+2*SD), fill = Model), alpha = 0.2)
}
```

Function to do plots for Brier Score

```
plotPec <- function(pecx){
  nvar <- length(pecx$AppErr)
  xx <- data.frame("time" = rep(pecx$time, nvar),
                   "AppErr" = unlist(pecx$AppErr),
                   "Model" = rep(names(pecx$AppErr), each = length(pecx$time)))
  ggplot(xx, aes(x = time, y = AppErr, col = Model)) +
    geom_line() +
    ylab("Prediction Error") +
    ylim(c(0,0.3))
}

plotPecList <- function(pecx){
  nvar <- length(pecx[[1]][["AppErr"]])
  xx <- list()
  for(i in 1:length(pecx)){
    xx[[i]] <- data.frame("time" = rep(pecx[[i]][["time"]], nvar),
                          "AppErr" = unlist(pecx[[i]][["AppErr"]]),
                          "Model" = rep(names(pecx[[i]][["AppErr"]]),
                          each = length(pecx[[i]][["time"]])))
  }
  p <- ggplot(xx[[i]], aes(x = time, y = AppErr, col = Model)) +
    geom_line() +
    ylab("Prediction Error") +
    ylim(c(0,0.3))

  for(i in 2:length(xx)){
    p <- p + geom_line(data = xx[[i]])
  }
  return(p)
}

plotPecListMean <- function(pecx){
  models   <- names(pecx[[1]][["AppErr"]])
```

```r
  plotData <- data.frame("Model" = NULL,"time" =NULL, "Mean" = NULL, "SD" = NULL)

  for(m in models){
    y <- sapply(pecx, function(x) x[["AppErr"]][[m]])
    means <- rowMeans(y)
    sds   <- rowSds(y)
    temp <- data.frame("Model" = rep(m, length(means)),
                       "time"  = pecx[[1]][["time"]],
                       "Mean"  = means,
                       "SD"    = sds)
    plotData = rbind(plotData, temp)
  }
  ggplot(plotData, aes(x = time, y = Mean, col = Model)) +
    geom_line() +
    ylab("Prediction Error") +
    ylim(c(0.1,0.3)) + xlim(c(1,5)) +
    geom_ribbon(aes(x = time, ymin = (Mean -2*SD),
                    ymax = (Mean+2*SD), fill = Model), alpha = 0.2)
}
```

Specify times of interest

```r
evalTimes <- seq(0,5, by = 0.5)
```

In this section we perform a more formal 10 fold cross validation of the different models. we randomly split the data into ten groups, and then iteratively train on 9/10 of the data and predict on the remaining 1/10. We can then evalulate the performance of the model in the entire dataset.

Set parameters for cross validation

```r
nFolds <- 10
nRepsCV  <- 10
recompute.cv <- FALSE
```

Split data into training and validation. Train models and predict.

```r
results.cv.file <- file.path("../GeneratedData/results.cvRep.file.Rdata")

if(file.exists(results.cv.file) & recompute.cv == FALSE){
  load(results.cv.file)
} else{
  results.cv.reps <- list()
  times.interest  <- seq(0,5, by = 0.5)
  batchSize       <- table(combined.metadata$study)
  set.seed(191919)

  for(rep in 1:nRepsCV){
    results.cv <- list()
    results.cv.scores <- list()

    combined.metadata$cv <- c(sample(1:nFolds, batchSize[1], replace = TRUE),
                              sample(1:nFolds, batchSize[2], replace = TRUE),
                              sample(1:nFolds, batchSize[3], replace = TRUE))


    ## Loop over different subsets of data
```

```r
for(subData in c("ALL", "ABC", "GCB")){

  ## Select subset of metadata
  if(subData != "ALL"){
    combined.metadata2 <- subset(combined.metadata, ABCGCB == subData)
  } else{
    combined.metadata2 <- combined.metadata
  }

  ## Dicotomize IPI
  combined.metadata2$IPI  <- factor(ifelse(combined.metadata2$IPI %in% c("0", "1"), "0-1", "2-5"))

  ## Prepare matrices for output
  predictCoxIPI <- matrix(NA, ncol = length(times.interest), nrow = nrow(combined.metadata2),
                          dimnames = list(combined.metadata2$ID))
  predictCoxAge <- matrix(NA, ncol = length(times.interest), nrow = nrow(combined.metadata2),
                          dimnames = list(combined.metadata2$ID))
  predictCoxMIR <- matrix(NA, ncol = length(times.interest), nrow = nrow(combined.metadata2),
                          dimnames = list(combined.metadata2$ID))
  predictRSF <- matrix(NA, ncol = length(times.interest), nrow = nrow(combined.metadata2),
                          dimnames = list(combined.metadata2$ID))
  predictCoxMIRIPI <- matrix(NA, ncol = length(times.interest), nrow = nrow(combined.metadata2),
                          dimnames = list(combined.metadata2$ID))
  predictRSFMIRIPI <- matrix(NA, ncol = length(times.interest), nrow = nrow(combined.metadata2),
                          dimnames = list(combined.metadata2$ID))

  ## Matrices for linear predictions
  scoreCoxMIR <- matrix(NA, ncol = 1, nrow = nrow(combined.metadata2),
                          dimnames = list(combined.metadata2$ID))
  scoreCoxMIRIPI <- matrix(NA, ncol = 1, nrow = nrow(combined.metadata2),
                          dimnames = list(combined.metadata2$ID))

  ## Loop over folds
  for(i in 1:nFolds){

    ## Select meta and gep data for training and validation
    meta.train <- subset(combined.metadata2, cv != i)
    meta.val   <- subset(combined.metadata2, cv == i)
    gep.train.sc <- microarrayScale(combined.data.combat[probes, meta.train$ID], scale = FALSE)
    gep.val.sc   <- microarrayScale(combined.data.combat[probes, meta.val$ID])

    ## Fit cox models with age or IPI
    fit.cox.age <- coxph(PFS ~ age, data = meta.train)
    fit.cox.IPI <- coxph(PFS ~ IPI, data = meta.train)

    ## Fit Random survival forest w/ wo IPI
    trainInput <- data.frame("time"=meta.train$PFS[,1],
                             "status"=meta.train$PFS[,2], t(gep.train.sc))
    fit.rsf <- rfsrc(formula = Surv(time, status)~.,
                  data = trainInput,
                  ntree = 1000)

    trainInput$IPI <- meta.train$IPI
```

```r
fit.rsf.IPI <- rfsrc(formula = Surv(time, status)~.,
                     data = trainInput,
                     ntree = 1000)

## Fit cox models with MIR by first doing univariate cox for each probe
## adjusted for effect of study, and then selecting the significant probes
cox.data <- data.frame(meta.train, t(gep.train.sc))
names(cox.data) <- c(names(meta.train), probes)

cox.temp   <- uni.multi.cox(cox.data, probes, outcome = "PFS")
sig.probes <- row.names(cox.temp$combined.results[cox.temp$combined.results$uni.p<0.05,])

trainInput.sig.probes <- data.frame(meta.train$PFS, t(gep.train.sc[sig.probes,,drop=FALSE]))
names(trainInput.sig.probes) <- c("PFS", sig.probes)
fit.cox.MIR <- coxph(PFS~., data = trainInput.sig.probes)

## Add IPI to MIR prediction
trainInput.sig.probes$IPI <- meta.train$IPI
fit.cox.MIRIPI <- coxph(PFS~., data = trainInput.sig.probes)

## Get standard deviation of probes in training data and build validation data
trainSDs <- rowSds(combined.data.combat[probes, meta.train$ID])
valInput <- data.frame(meta.val$age, meta.val$IPI, t(gep.val.sc*trainSDs))
names(valInput) <- c("age", "IPI", probes)
valInput.rsf <- data.frame(valInput[,probes])
valInput.rsf.ipi <- data.frame(valInput[,-1])

## Predict in validation data and save results
predictCoxAge[meta.val$ID,] <- predictSurvProb(fit.cox.age,
                                               newdata = valInput,
                                               times = times.interest)
predictCoxIPI[meta.val$ID,] <- predictSurvProb(fit.cox.IPI,
                                               newdata = valInput,
                                               times = times.interest)
if(!is.null(coef(fit.cox.MIR))){
  predictCoxMIR[meta.val$ID,] <- predictSurvProb(fit.cox.MIR,
                                                 newdata = valInput,
                                                 times = times.interest)
}
predictRSF[meta.val$ID,]    <- predictSurvProb(fit.rsf,
                                               newdata = valInput.rsf,
                                               times = times.interest)
predictCoxMIRIPI[meta.val$ID,] <- predictSurvProb(fit.cox.MIRIPI,
                                                  newdata = valInput,
                                                  times = times.interest)
predictRSFMIRIPI[meta.val$ID,] <- predictSurvProb(fit.rsf.IPI,
                                                  newdata = valInput.rsf.ipi,
                                                  times = times.interest)

## Linear predictions for coxMIR models
if(!is.null(coef(fit.cox.MIR))){
  scoreCoxMIR[meta.val$ID,1]    <- predict(fit.cox.MIR, newdata = valInput)
}
```

```
        scoreCoxMIRIPI[meta.val$ID,1] <- predict(fit.cox.MIRIPI, newdata = valInput)


      }
      results.cv[[subData]] <- list("CoxAge" = predictCoxAge,
                                    "CoxIPI" = predictCoxIPI,
                                    "CoxMIR" = predictCoxMIR,
                                    "RSFMIR"    = predictRSF,
                                    "CoxMIRIPI" = predictCoxMIRIPI,
                                    "RSFMIRIPI" = predictRSFMIRIPI)
      results.cv.scores[[subData]] <- list("scoreCoxMIR" = scoreCoxMIR,
                                           "scoreCoxMIRIPI" = scoreCoxMIRIPI)
    }
    results.cv.reps[[rep]] <- list("cv" = results.cv, "scores" = results.cv.scores)

  }
  save(results.cv.reps, times.interest, file = results.cv.file, nRepsCV)
}
```

Results of CV on OS

We first evaluate the predictive accuracy of the model on overall survival.

Calculate brier score for OS

```
error.all.OS <- list()
error.abc.OS <- list()
error.gcb.OS <- list()

for(i in 1:nRepsCV){
error.all.OS[[i]] <- pec(results.cv.reps[[i]][["cv"]][["ALL"]],
                         formula = Surv(OStime,OSstatus) ~ 1,
                         data = combined.metadata,
                         times = times.interest,
                         reference = F,
                         verbose = T, exact = FALSE)




error.abc.OS[[i]] <- pec(results.cv.reps[[i]][["cv"]][["ABC"]],
                         formula = Surv(OStime,OSstatus) ~ 1,
                         data = subset(combined.metadata, ABCGCB == "ABC"),
                         times = times.interest,
                         reference = F,
                         verbose = T, exact = FALSE)


error.gcb.OS[[i]] <- pec(results.cv.reps[[i]][["cv"]][["GCB"]],
                         formula = Surv(OStime,OSstatus) ~ 1,
                         data = subset(combined.metadata, ABCGCB == "GCB"),
                         times = times.interest,
                         reference = F,
                         verbose = T, exact = FALSE)
```

```
}
```

```
## No covariates  specified: Kaplan-Meier for censoring times used for weighting.
## No covariates  specified: Kaplan-Meier for censoring times used for weighting.
## No covariates  specified: Kaplan-Meier for censoring times used for weighting.
## No covariates  specified: Kaplan-Meier for censoring times used for weighting.
## No covariates  specified: Kaplan-Meier for censoring times used for weighting.
## No covariates  specified: Kaplan-Meier for censoring times used for weighting.
## No covariates  specified: Kaplan-Meier for censoring times used for weighting.
## No covariates  specified: Kaplan-Meier for censoring times used for weighting.
## No covariates  specified: Kaplan-Meier for censoring times used for weighting.
## No covariates  specified: Kaplan-Meier for censoring times used for weighting.
## No covariates  specified: Kaplan-Meier for censoring times used for weighting.
## No covariates  specified: Kaplan-Meier for censoring times used for weighting.
## No covariates  specified: Kaplan-Meier for censoring times used for weighting.
## No covariates  specified: Kaplan-Meier for censoring times used for weighting.
## No covariates  specified: Kaplan-Meier for censoring times used for weighting.
## No covariates  specified: Kaplan-Meier for censoring times used for weighting.
## No covariates  specified: Kaplan-Meier for censoring times used for weighting.
## No covariates  specified: Kaplan-Meier for censoring times used for weighting.
## No covariates  specified: Kaplan-Meier for censoring times used for weighting.
## No covariates  specified: Kaplan-Meier for censoring times used for weighting.
## No covariates  specified: Kaplan-Meier for censoring times used for weighting.
## No covariates  specified: Kaplan-Meier for censoring times used for weighting.
## No covariates  specified: Kaplan-Meier for censoring times used for weighting.
## No covariates  specified: Kaplan-Meier for censoring times used for weighting.
## No covariates  specified: Kaplan-Meier for censoring times used for weighting.
## No covariates  specified: Kaplan-Meier for censoring times used for weighting.
## No covariates  specified: Kaplan-Meier for censoring times used for weighting.
## No covariates  specified: Kaplan-Meier for censoring times used for weighting.
## No covariates  specified: Kaplan-Meier for censoring times used for weighting.
## No covariates  specified: Kaplan-Meier for censoring times used for weighting.
```

Calucate time varying AUCs

```
tAUC.all.OS <- list()
tAUC.abc.OS <- list()
tAUC.gcb.OS <- list()

tAUC.OS.ALL <- function(x) timeVaryingAUC(x, combined.metadata$OStime,
                                          combined.metadata$OSstatus, times.interest)
tAUC.OS.ABC <- function(x) timeVaryingAUC(x, subset(combined.metadata, ABCGCB == "ABC")$OStime,
                                      subset(combined.metadata, ABCGCB == "ABC")$OSstatus,
                                      times.interest)
tAUC.OS.GCB <- function(x) timeVaryingAUC(x, subset(combined.metadata, ABCGCB == "GCB")$OStime,
                                      subset(combined.metadata, ABCGCB == "GCB")$OSstatus,
                                      times.interest)

for(rep in 1:nRepsCV){
  tAUC.all.OS[[rep]] <- lapply(results.cv.reps[[rep]][["cv"]][["ALL"]], tAUC.OS.ALL)
  tAUC.abc.OS[[rep]] <- lapply(results.cv.reps[[rep]][["cv"]][["ABC"]], tAUC.OS.ABC)
  tAUC.gcb.OS[[rep]] <- lapply(results.cv.reps[[rep]][["cv"]][["GCB"]], tAUC.OS.GCB)
}
```

Plot Results

```r
# Brier Scores
p11 <- plotPecListMean(error.all.OS) + ggtitle("ALL: OS") +
  scale_color_discrete(guide = FALSE) +
  xlab("") + scale_fill_discrete(guide = FALSE) +
  labs(tag = "A")
p12 <- plotPecListMean(error.abc.OS) + ggtitle("ABC: OS") +
  scale_color_discrete(guide = FALSE) +
  xlab("") + scale_fill_discrete(guide = FALSE) +
  ylab("") + labs(tag = "B")
p13 <- plotPecListMean(error.gcb.OS) + ggtitle("GCB: OS") +
  xlab("") + ylab("") + labs(tag = "C")

#tAUC
p21 <- plottAUCListMean(tAUC.all.OS, times.interest)  +
  scale_color_discrete(guide = FALSE) +
  scale_fill_discrete(guide = FALSE) + labs(tag = "D")
p22 <- plottAUCListMean(tAUC.abc.OS, times.interest)  +
  scale_color_discrete(guide = FALSE)  +
  ylab("")  + scale_fill_discrete(guide = FALSE) + labs(tag = "E")
p23 <- plottAUCListMean(tAUC.gcb.OS, times.interest) +
  ylab("") + labs(tag = "F")

grid.arrange(p11,p12,p13,p21,p22,p23, ncol = 3, widths = c(2,2,3.2))
```

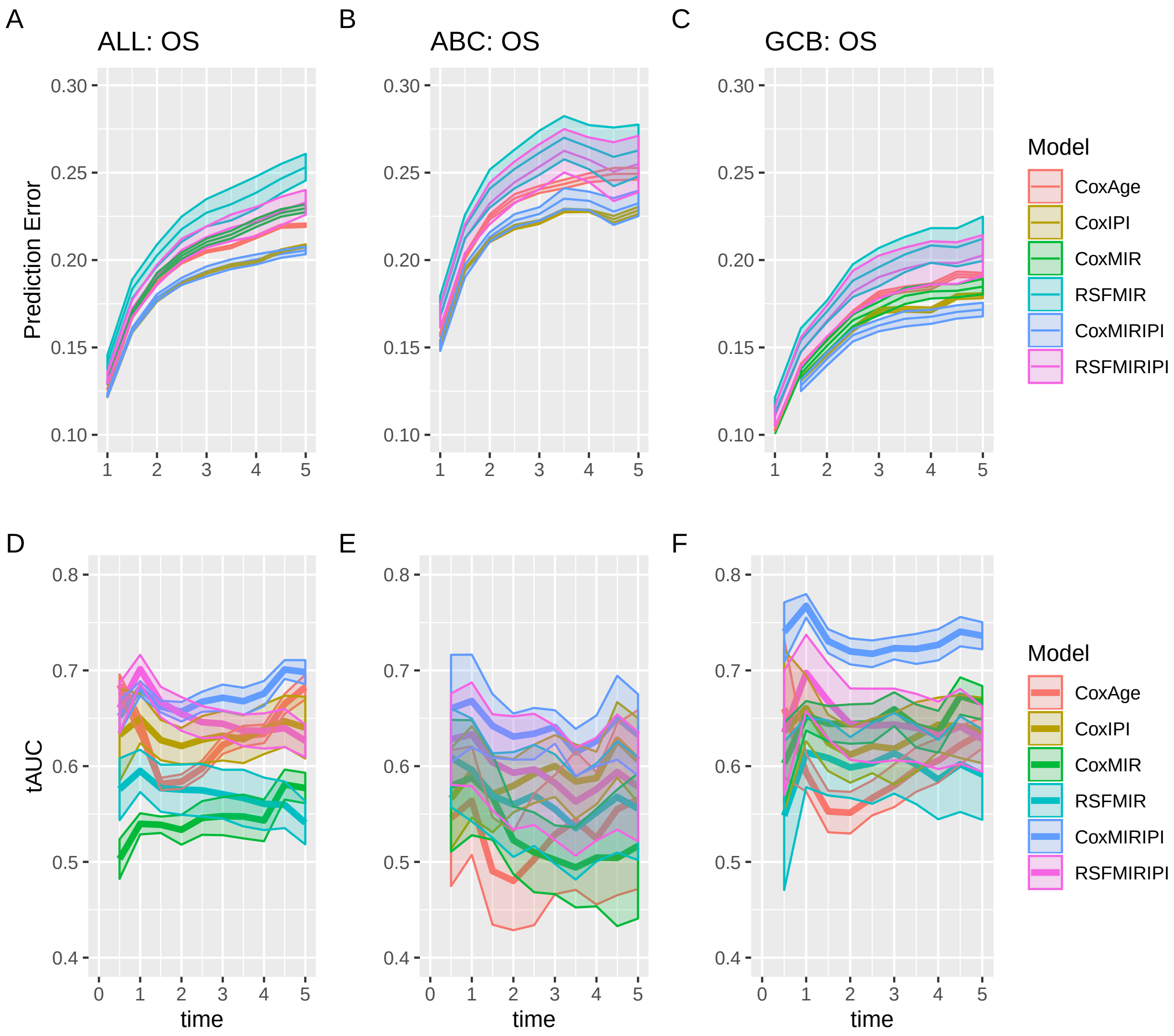

Results of CV on PFS

Next the predictive accuracy for progression free survival is evaluated.

Calculate brier score for PFS

```
error.all.PFS <- list()
error.abc.PFS <- list()
error.gcb.PFS <- list()

for(i in 1:nRepsCV){
error.all.PFS[[i]] <- pec(results.cv.reps[[i]][["cv"]][["ALL"]],
                          formula = Surv(PFStime,PFSstatus) ~ 1,
                          data = combined.metadata,
                          times = times.interest,
                          reference = F,
                          verbose = T, exact = FALSE)
```

```
error.abc.PFS[[i]] <- pec(results.cv.reps[[i]][["cv"]][["ABC"]],
                          formula = Surv(PFStime,PFSstatus) ~ 1,
                          data = subset(combined.metadata, ABCGCB == "ABC"),
                          times = times.interest,
                          reference = F,
                          verbose = T, exact = FALSE)


error.gcb.PFS[[i]] <- pec(results.cv.reps[[i]][["cv"]][["GCB"]],
                          formula = Surv(PFStime,PFSstatus) ~ 1,
                          data = subset(combined.metadata, ABCGCB == "GCB"),
                          times = times.interest,
                          reference = F,
                          verbose = T, exact = FALSE)
}
```

```
## No covariates  specified: Kaplan-Meier for censoring times used for weighting.
## No covariates  specified: Kaplan-Meier for censoring times used for weighting.
## No covariates  specified: Kaplan-Meier for censoring times used for weighting.
## No covariates  specified: Kaplan-Meier for censoring times used for weighting.
## No covariates  specified: Kaplan-Meier for censoring times used for weighting.
## No covariates  specified: Kaplan-Meier for censoring times used for weighting.
## No covariates  specified: Kaplan-Meier for censoring times used for weighting.
## No covariates  specified: Kaplan-Meier for censoring times used for weighting.
## No covariates  specified: Kaplan-Meier for censoring times used for weighting.
## No covariates  specified: Kaplan-Meier for censoring times used for weighting.
## No covariates  specified: Kaplan-Meier for censoring times used for weighting.
## No covariates  specified: Kaplan-Meier for censoring times used for weighting.
## No covariates  specified: Kaplan-Meier for censoring times used for weighting.
## No covariates  specified: Kaplan-Meier for censoring times used for weighting.
## No covariates  specified: Kaplan-Meier for censoring times used for weighting.
## No covariates  specified: Kaplan-Meier for censoring times used for weighting.
## No covariates  specified: Kaplan-Meier for censoring times used for weighting.
## No covariates  specified: Kaplan-Meier for censoring times used for weighting.
## No covariates  specified: Kaplan-Meier for censoring times used for weighting.
## No covariates  specified: Kaplan-Meier for censoring times used for weighting.
## No covariates  specified: Kaplan-Meier for censoring times used for weighting.
## No covariates  specified: Kaplan-Meier for censoring times used for weighting.
## No covariates  specified: Kaplan-Meier for censoring times used for weighting.
## No covariates  specified: Kaplan-Meier for censoring times used for weighting.
## No covariates  specified: Kaplan-Meier for censoring times used for weighting.
## No covariates  specified: Kaplan-Meier for censoring times used for weighting.
## No covariates  specified: Kaplan-Meier for censoring times used for weighting.
## No covariates  specified: Kaplan-Meier for censoring times used for weighting.
## No covariates  specified: Kaplan-Meier for censoring times used for weighting.
## No covariates  specified: Kaplan-Meier for censoring times used for weighting.
```

Calucate time varying AUCs

```
tAUC.all.PFS <- list()
tAUC.abc.PFS <- list()
tAUC.gcb.PFS <- list()

tAUC.PFS.ALL <- function(x) timeVaryingAUC(x, combined.metadata$PFStime,
```

```r
                                          combined.metadata$PFSstatus,
                                          times.interest)
tAUC.PFS.ABC <- function(x) timeVaryingAUC(x, subset(combined.metadata, ABCGCB == "ABC")$PFStime,
                                     subset(combined.metadata, ABCGCB == "ABC")$PFSstatus,
                                     times.interest)
tAUC.PFS.GCB <- function(x) timeVaryingAUC(x, subset(combined.metadata, ABCGCB == "GCB")$PFStime,
                                     subset(combined.metadata, ABCGCB == "GCB")$PFSstatus,
                                     times.interest)

for(rep in 1:nRepsCV){
  tAUC.all.PFS[[rep]] <- lapply(results.cv.reps[[rep]][["cv"]][["ALL"]], tAUC.PFS.ALL)
  tAUC.abc.PFS[[rep]] <- lapply(results.cv.reps[[rep]][["cv"]][["ABC"]], tAUC.PFS.ABC)
  tAUC.gcb.PFS[[rep]] <- lapply(results.cv.reps[[rep]][["cv"]][["GCB"]], tAUC.PFS.GCB)
}
```

Plot Results

```r
# Brier Scores
p11 <- plotPecListMean(error.all.PFS) + ggtitle("ALL: PFS") +
  scale_color_discrete(guide = FALSE) +
  scale_fill_discrete(guide = FALSE) +
  xlab("") + labs(tag = "A")
p12 <- plotPecListMean(error.abc.PFS) + ggtitle("ABC: PFS") +
  scale_color_discrete(guide = FALSE) +
  scale_fill_discrete(guide = FALSE) +
  xlab("") +ylab("") + labs(tag = "B")
p13 <- plotPecListMean(error.gcb.PFS) + ggtitle("GCB: PFS") +
  xlab("") + ylab("") + labs(tag = "C")

#tAUC
p21 <- plottAUCListMean(tAUC.all.PFS, times.interest)  +
  scale_color_discrete(guide = FALSE) +
  scale_fill_discrete(guide = FALSE) + labs(tag = "D")
p22 <- plottAUCListMean(tAUC.abc.PFS, times.interest)  +
  scale_color_discrete(guide = FALSE) +
  scale_fill_discrete(guide = FALSE) + ylab("") + labs(tag = "E")
p23 <- plottAUCListMean(tAUC.gcb.PFS, times.interest) +
  ylab("") + labs(tag = "F")

fig2 <- grid.arrange(p11,p12,p13,p21,p22,p23, ncol = 3, widths = c(2,2,3.2))
```

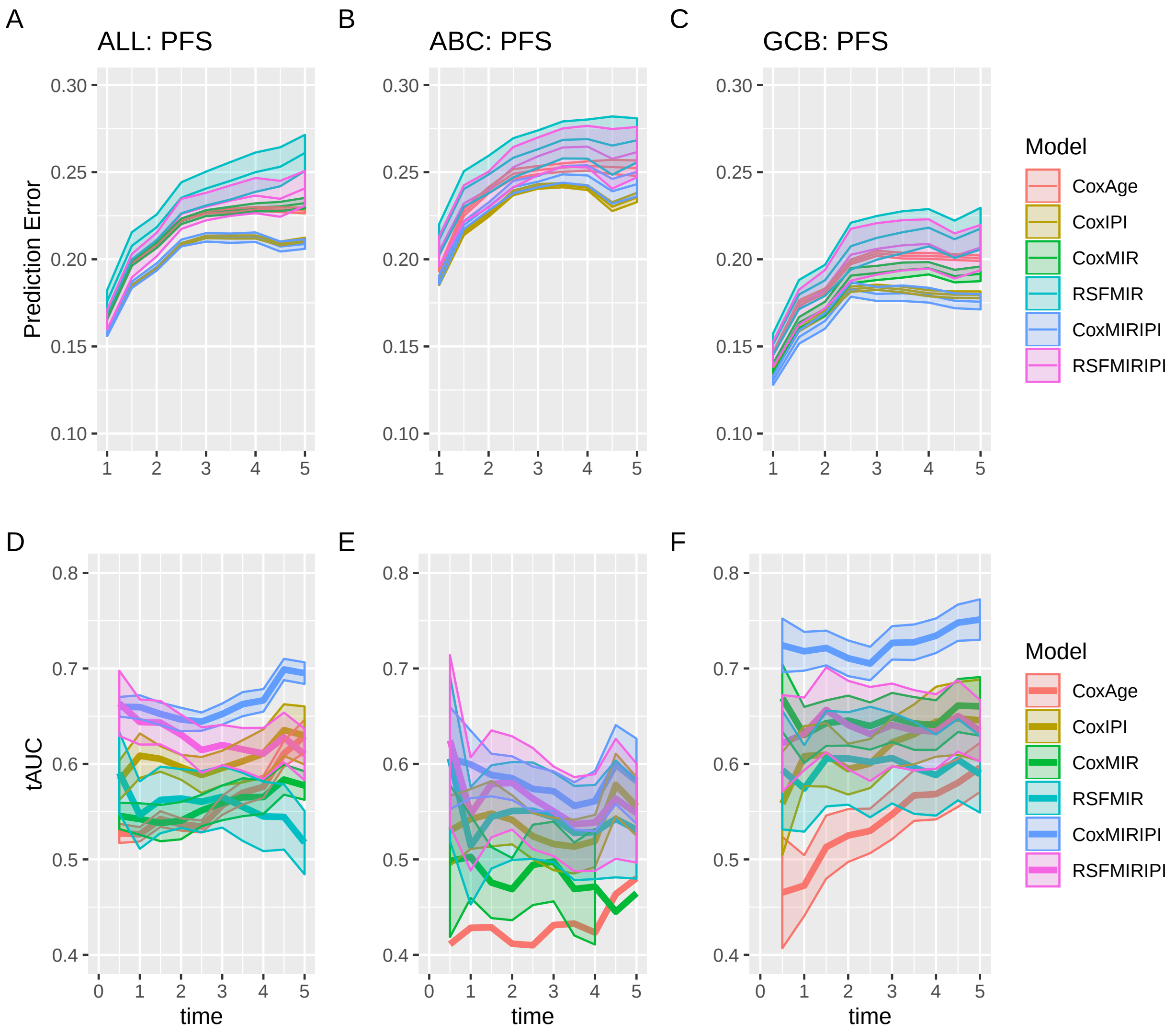
A
ALL: PFS
B
ABC: PFS
C
GCB: PFS
Prediction Error
Model
CoxAge
CoxIPI
CoxMIR
RSFMIR
CoxMIRIPI
RSFMIRIPI
D
E
F
tAUC
time
time
time
Model
CoxAge
CoxIPI
CoxMIR
RSFMIR
CoxMIRIPI
RSFMIRIPI

```
plot(fig2)
```

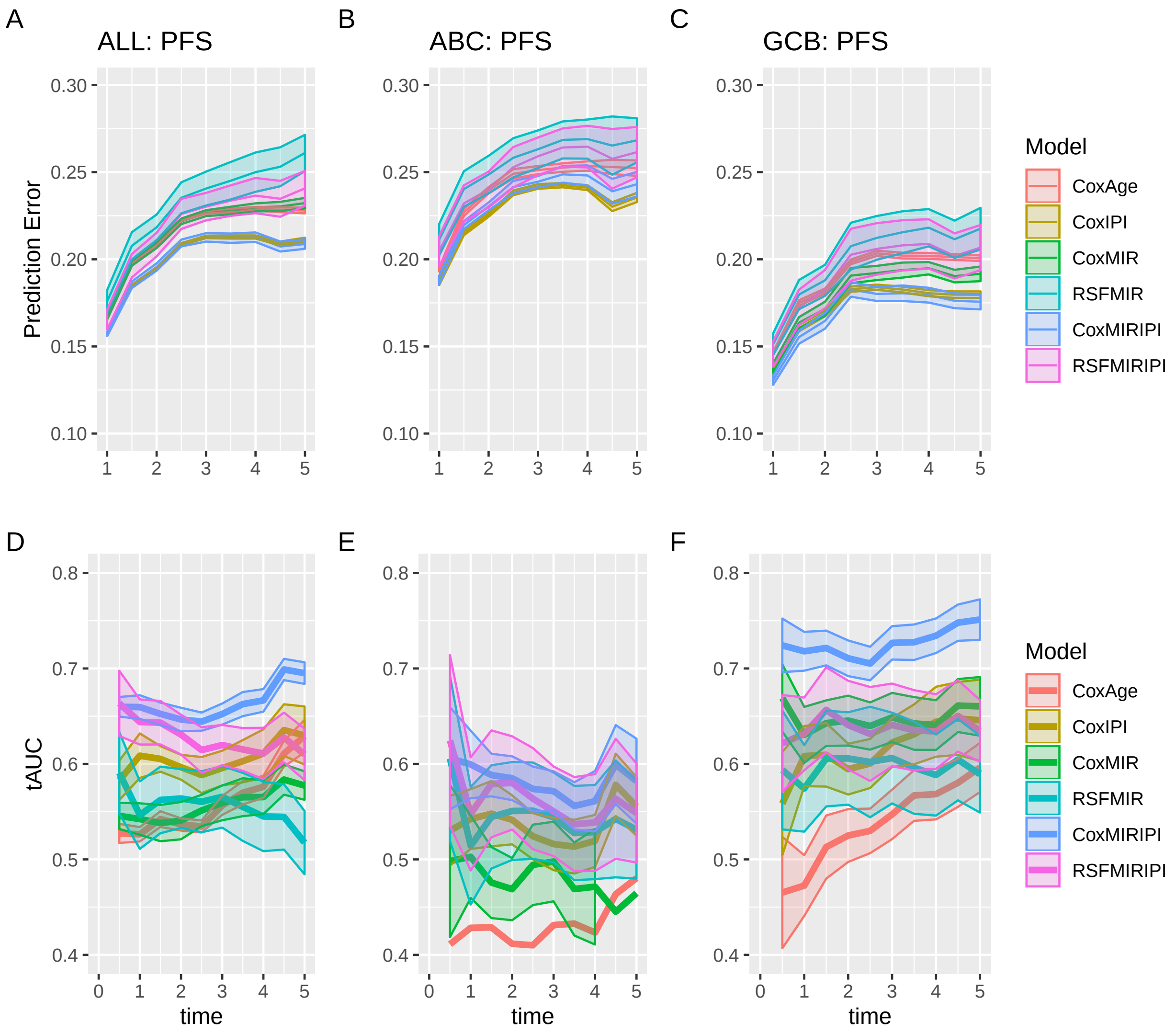


```
scoresMIR <- do.call(cbind, lapply(results.cv.reps,
                                   function(x) x[["scores"]][["GCB"]][["scoreCoxMIR"]]))
scoresMIRIPI <- do.call(cbind, lapply(results.cv.reps,
                                      function(x) x[["scores"]][["GCB"]][["scoreCoxMIRIPI"]]))

scores.gcb <- data.frame("coxMIR" = rowMeans(scoresMIR),
                         "coxMIRIPI" = rowMeans(scoresMIRIPI))
```

Kaplan meier plots

We use the linear predictions from the Cox models in the crossvalidation to get a score for each individual. These are then split into tertiles and kaplan meier plots are made. Start by adding linear predictions to GCB subset and splitting into tertiles.

```
combined.metadata.gcb <- merge(combined.metadata, scores.gcb,by.x = "ID", by.y = 0)
combined.metadata.gcb$coxMIRClass <- cutFun(combined.metadata.gcb$coxMIR)
combined.metadata.gcb$coxMIRIPIClass <- cutFun(combined.metadata.gcb$coxMIRIPI)
combined.metadata.gcb$Response <- factor(combined.metadata.gcb$Response,
```

```
                                         levels =  levels(combined.metadata.gcb$Response)[c(1,3,4,2)])
```

Then do KM plots

```
fitMIROS  <- with(combined.metadata.gcb, survfit(OS5~coxMIRClass))
fitMIRPFS <- with(combined.metadata.gcb, survfit(PFS5~coxMIRClass))

kmMIROS <- ggsurvplot(fitMIROS,
                    risk.table = TRUE,
                    data = combined.metadata.gcb,
                    risk.table.y.text=FALSE,
                    pval = TRUE,
                    tables.height = 0.3,
                    legend.labs = c("Low", "Intermediate", "High")) +
          ggtitle("MIR: OS")

kmMIRPFS <- ggsurvplot(fitMIRPFS,
                    risk.table = TRUE,
                    data = combined.metadata.gcb,
                    risk.table.y.text=FALSE,
                    pval = TRUE,
                    tables.height = 0.3,
                    legend.labs = c("Low", "Intermediate", "High")) +
          ggtitle("MIR: PFS")
```

Try with coxMIRIPI

```
fitMIRIPIOS  <- with(combined.metadata.gcb, survfit(OS5~coxMIRIPIClass))
fitMIRIPIPFS <- with(combined.metadata.gcb, survfit(PFS5~coxMIRIPIClass))

kmMIRIPIOS <- ggsurvplot(fitMIRIPIOS,
                    risk.table = TRUE,
                    data = combined.metadata.gcb,
                    risk.table.y.text=FALSE,
                    pval = TRUE,
                    tables.height = 0.3,
                    legend.labs = c("Low", "Intermediate", "High")) +
                    ggtitle("MIR+IPI: OS")

kmMIRIPIPFS <- ggsurvplot(fitMIRIPIPFS,
                    data = combined.metadata.gcb,
                    risk.table = TRUE,
                    risk.table.y.text=FALSE,
                    pval = TRUE,
                    tables.height = 0.3,
                    legend.labs = c("Low", "Intermediate", "High")) +
                    ggtitle("MIR+IPI: PFS")
```

Create side-by-side plots

```
kmMIROS <- kmMIROS + labs(tag = "A")
kmMIRIPIOS <- kmMIRIPIOS + labs(tag = "B")
kmMIRPFS <- kmMIRPFS + labs(tag = "A")
kmMIRIPIPFS <- kmMIRIPIPFS + labs(tag = "B")
```

```r
arrange_ggsurvplots(list(kmMIROS, kmMIRIPIOS))
```

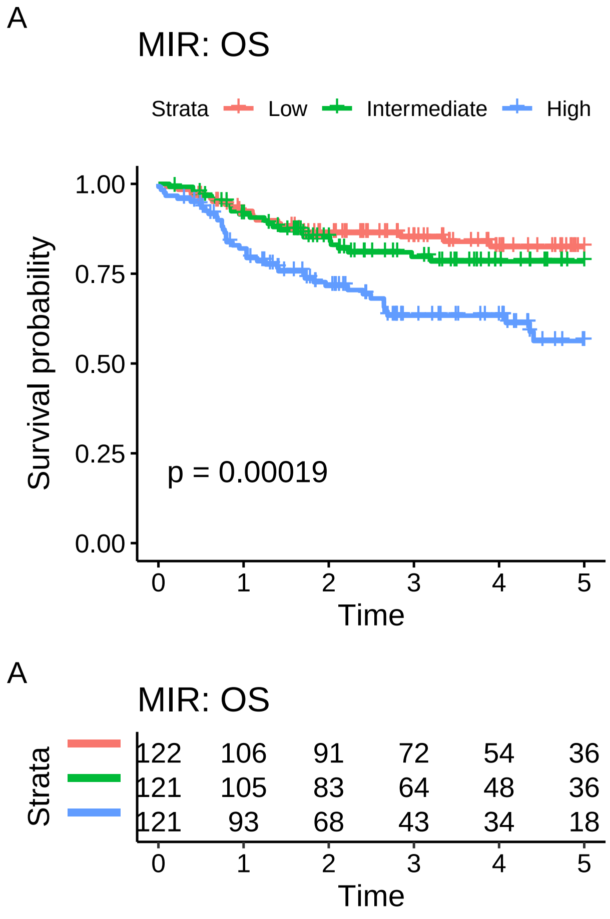


```r
fig3 <- arrange_ggsurvplots(list(kmMIRPFS, kmMIRIPIPFS))
```

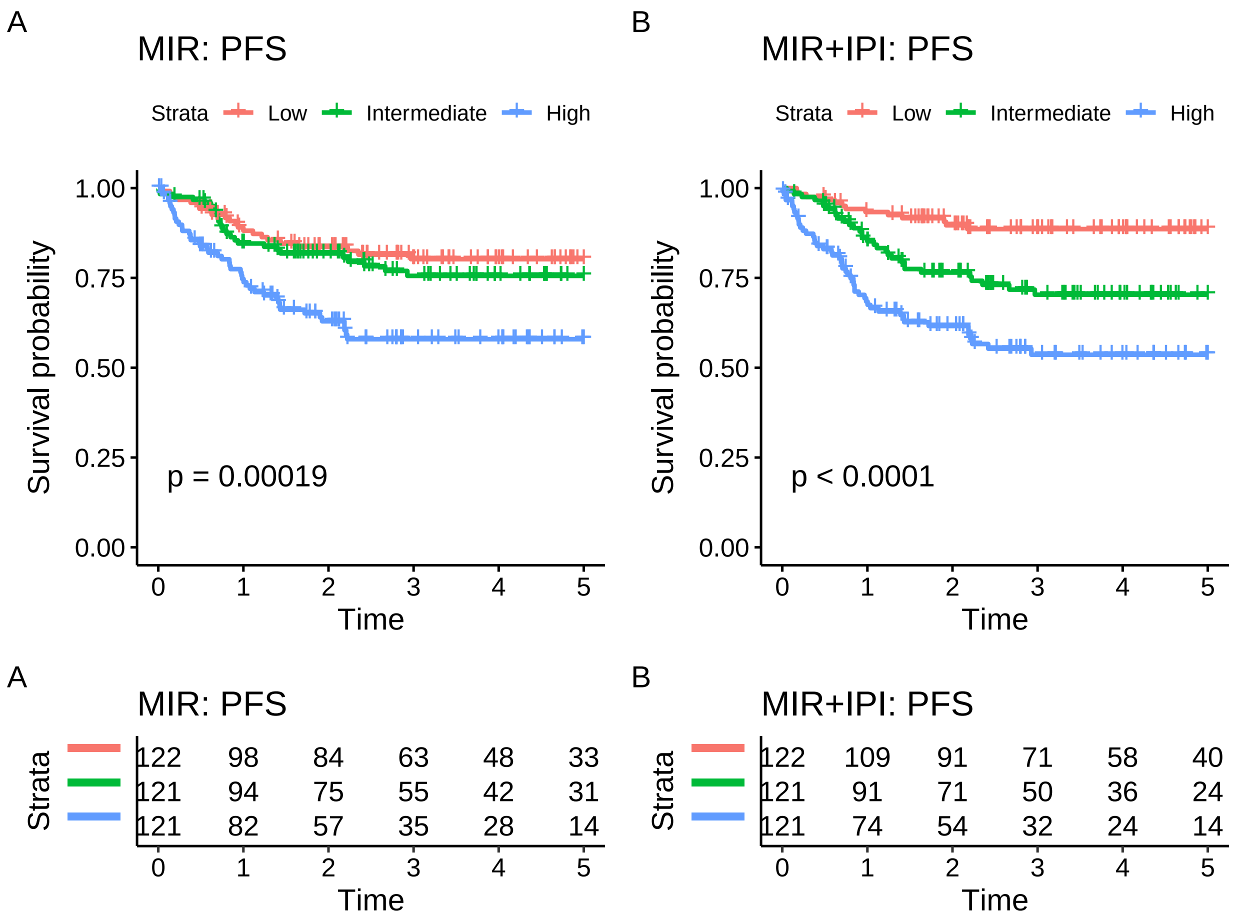

Stratify survival plots by study

```
kmMIROS_study <- ggsurvplot_facet(fitMIROS,
                                   data = combined.metadata.gcb,
                                   pval = TRUE,
                                   facet.by = "study") + ggtitle("MIR: OS")

kmMIRPFS_study <- ggsurvplot_facet(fitMIRPFS,
                                   data = combined.metadata.gcb,
                                   pval = TRUE,
                                   facet.by = "study")  + ggtitle("MIR: PFS")
kmMIRIPIOS_study <- ggsurvplot_facet(fitMIRIPIOS,
                                   data = combined.metadata.gcb,
                                   pval = TRUE,
                                   facet.by = "study")  + ggtitle("MIR+IPI: OS")

kmMIRIPIPFS_study <- ggsurvplot_facet(fitMIRIPIPFS,
                                   data = combined.metadata.gcb,
                                   pval = TRUE,
                                   facet.by = "study") + ggtitle("MIR+IPI: PFS")

kmMIROS_study
```

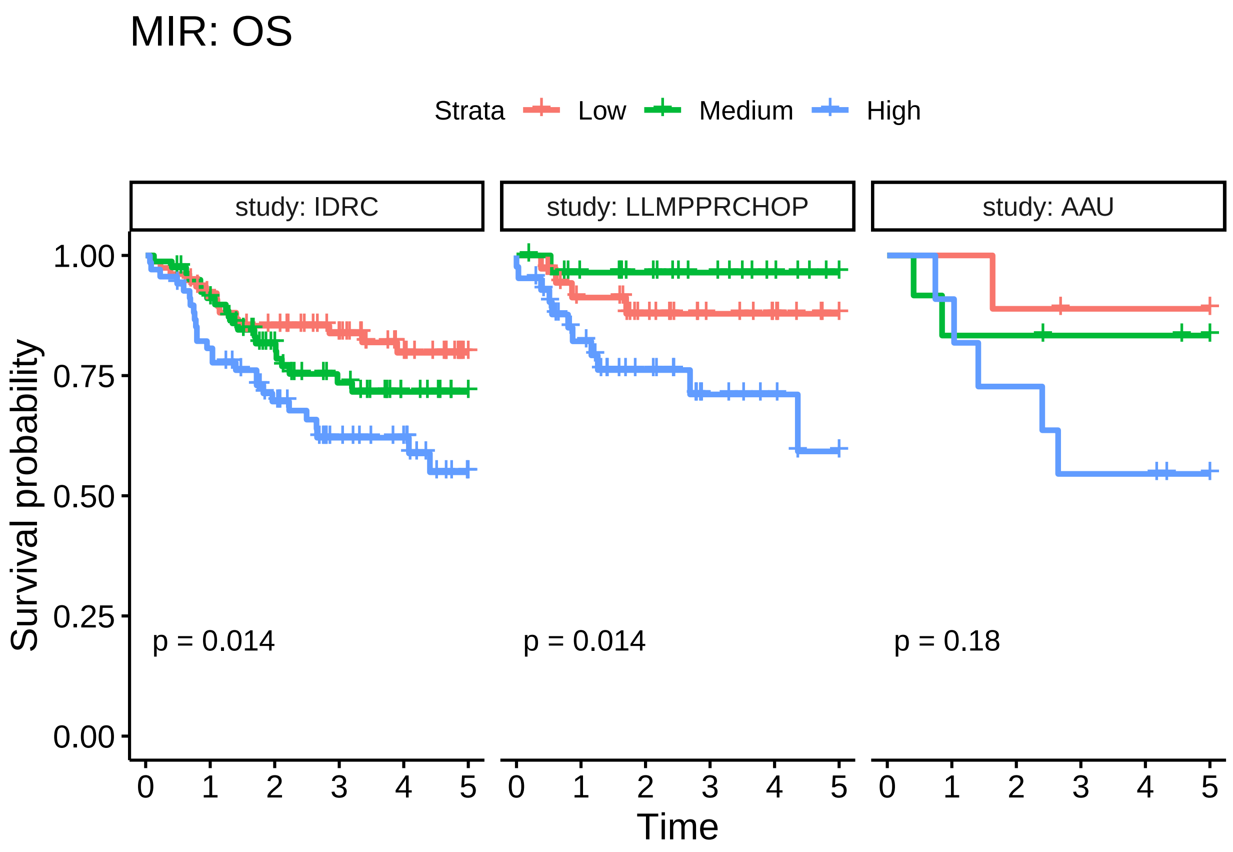


```
kmMIRPFS_study
```

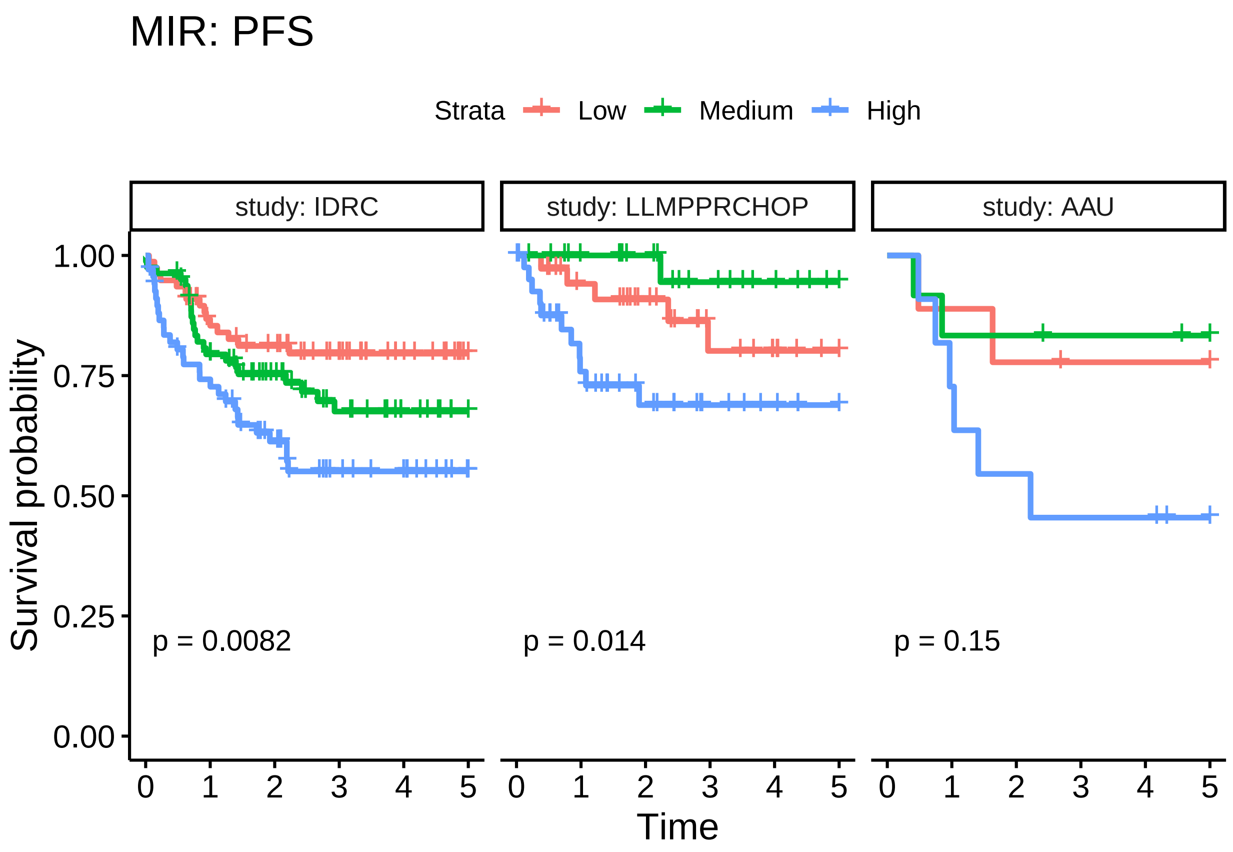


```
kmMIRIPIOS_study
```

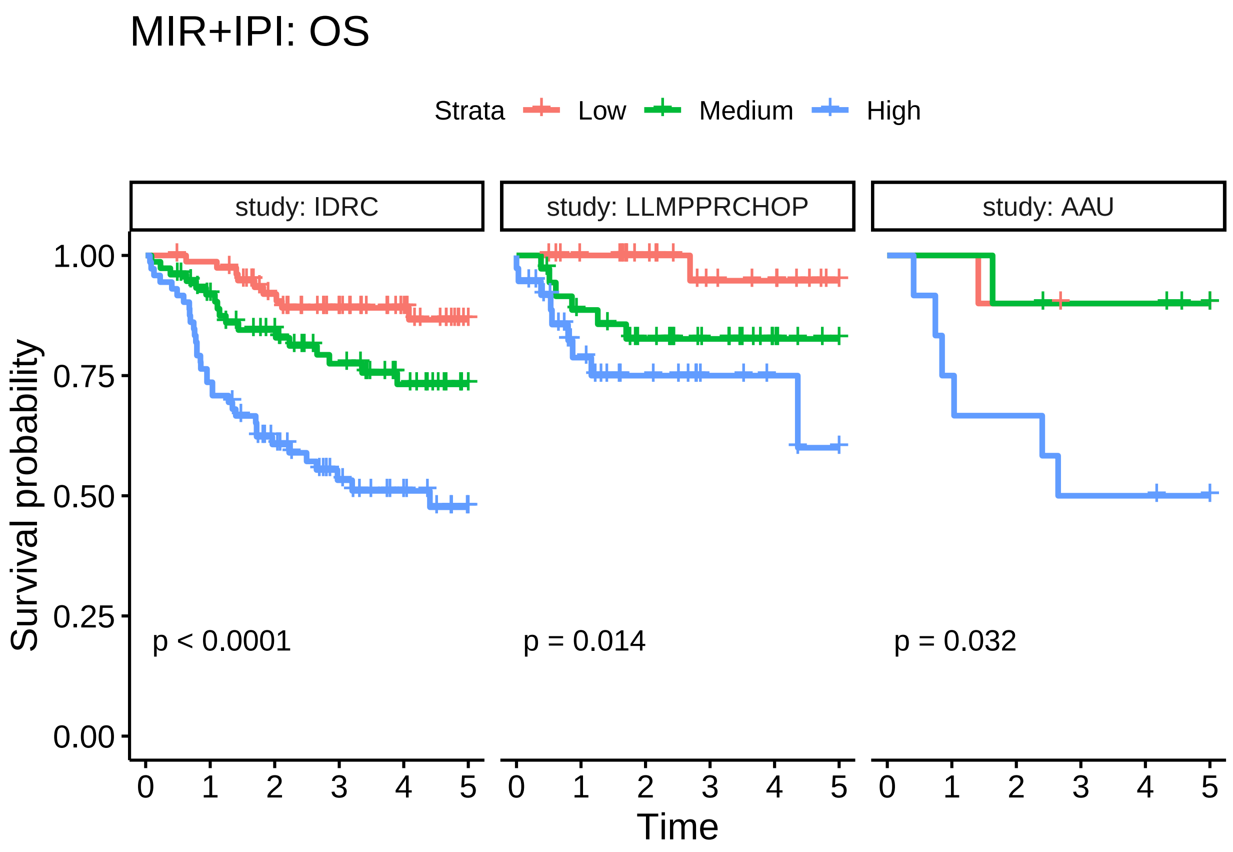


```
kmMIRIPIPFS_study
```

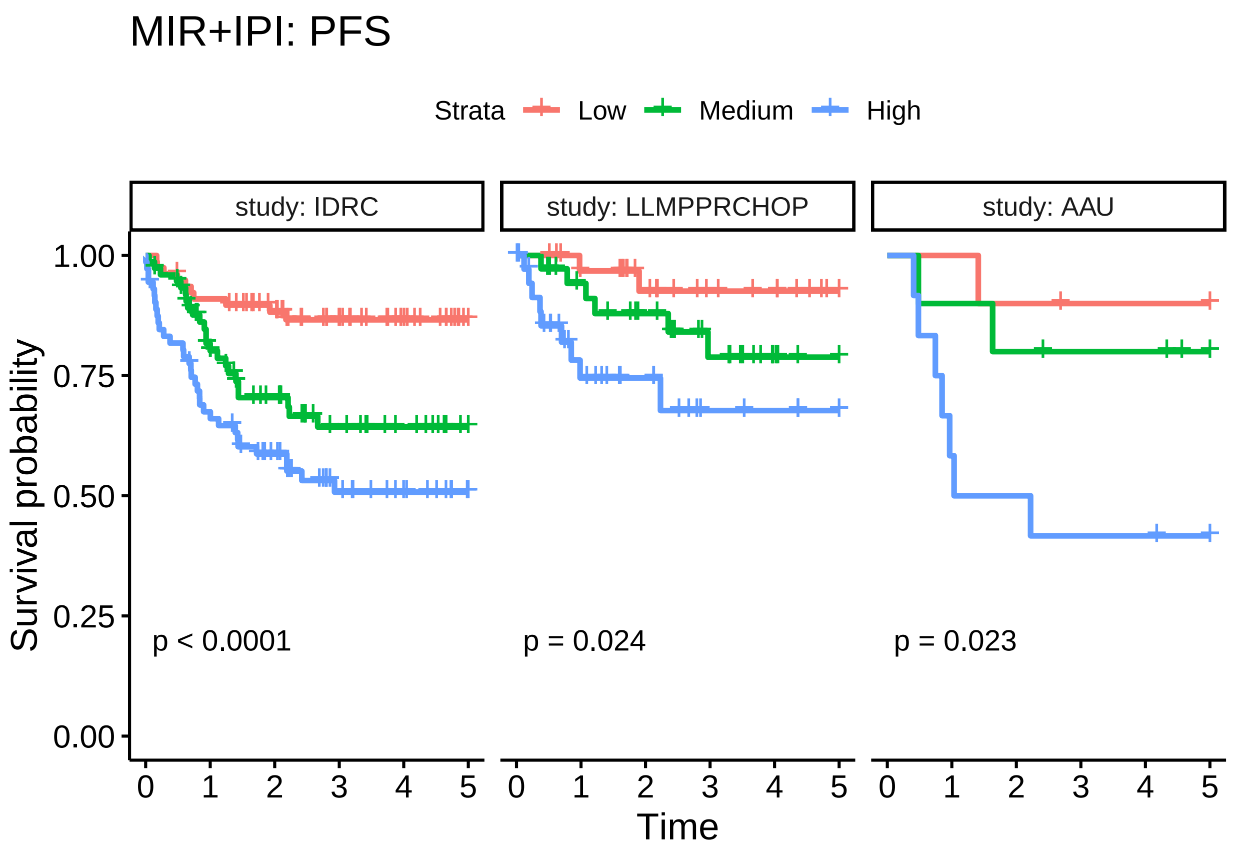

Compare with clinical response

Test for significant difference in prognostic score across clinical response groups and store p-values.

```
lmMIR    <- lm(coxMIR~Response, data = combined.metadata.gcb)
lmMIRIPI <- lm(coxMIRIPI~Response, data = combined.metadata.gcb)
summary(lmMIR)
```

```
##
## Call:
## lm(formula = coxMIR ~ Response, data = combined.metadata.gcb)
##
## Residuals:
##      Min       1Q   Median       3Q      Max
## -1.28027 -0.36027 -0.03468  0.32147  1.95475
##
## Coefficients:
##             Estimate Std. Error t value Pr(>|t|)
## (Intercept) -0.07897    0.03293  -2.398  0.01703 *
## ResponsePR   0.25209    0.08176   3.083  0.00223 **
## ResponseSD   0.17395    0.15326   1.135  0.25723
## ResponsePD   0.49665    0.12999   3.821  0.00016 ***
## ---
## Signif. codes:  0 '***' 0.001 '**' 0.01 '*' 0.05 '.' 0.1 ' ' 1
##
## Residual standard error: 0.5185 on 321 degrees of freedom
```

```
##   (39 observations deleted due to missingness)
## Multiple R-squared:  0.06532,    Adjusted R-squared:  0.05658
## F-statistic: 7.477 on 3 and 321 DF,  p-value: 7.472e-05
```

```
summary(lmMIRIPI)
```

```
##
## Call:
## lm(formula = coxMIRIPI ~ Response, data = combined.metadata.gcb)
##
## Residuals:
##      Min       1Q   Median       3Q      Max
## -1.67992 -0.65568  0.06101  0.57496  2.22702
##
## Coefficients:
##             Estimate Std. Error t value Pr(>|t|)
## (Intercept)  -0.1719     0.0482  -3.567 0.000417 ***
## ResponsePR    0.5955     0.1197   4.975 1.06e-06 ***
## ResponseSD    0.5421     0.2244   2.416 0.016240 *
## ResponsePD    0.7858     0.1903   4.129 4.65e-05 ***
## ---
## Signif. codes:  0 '***' 0.001 '**' 0.01 '*' 0.05 '.' 0.1 ' ' 1
##
## Residual standard error: 0.7591 on 321 degrees of freedom
##   (39 observations deleted due to missingness)
## Multiple R-squared:  0.1141, Adjusted R-squared:  0.1058
## F-statistic: 13.78 on 3 and 321 DF,  p-value: 1.775e-08
```

```
## Get p-values
pMIR <- ifelse(anova(lmMIR)$`Pr(>F)`[1] < 0.001,
               "p < 0.001",
               paste("p =", round(anova(lmMIR)$`Pr(>F)`[1]),3))


pMIRIPI <- ifelse(anova(lmMIRIPI)$`Pr(>F)`[1] < 0.001,
               "p < 0.001",
               paste("p =", round(anova(lmMIRIPI)$`Pr(>F)`[1]),3))
```

Create boxplots of clinical response vs prognostic score.

```
## Create Labels for x-axis
labls <-  c("Complete Response", "Partial Response", "Stable Disease", "Progressive Disease")
labls <-  paste0(labls, "(N =", table(combined.metadata.gcb$Response), ")")


crMIR <- ggplot(subset(combined.metadata.gcb, is.na(Response) == FALSE),
             aes(x = Response, y = coxMIR, fill = Response)) +
             geom_boxplot() +
             ggtitle("MIR: Clinical Response") +
             scale_fill_discrete(labels = labls) +
             annotate("text", label = pMIR, x = 3, y = 1.9)

crMIRIPI <- ggplot(subset(combined.metadata.gcb, is.na(Response) == FALSE),
               aes(x = Response, y = coxMIRIPI, fill = Response)) +
               geom_boxplot() +
               ggtitle("MIR+IPI: Clinical Response") +
```

```
            scale_fill_discrete(labels = labls)  +
            annotate("text", label = pMIRIPI, x = 3, y = 1.9)
crMIR
```

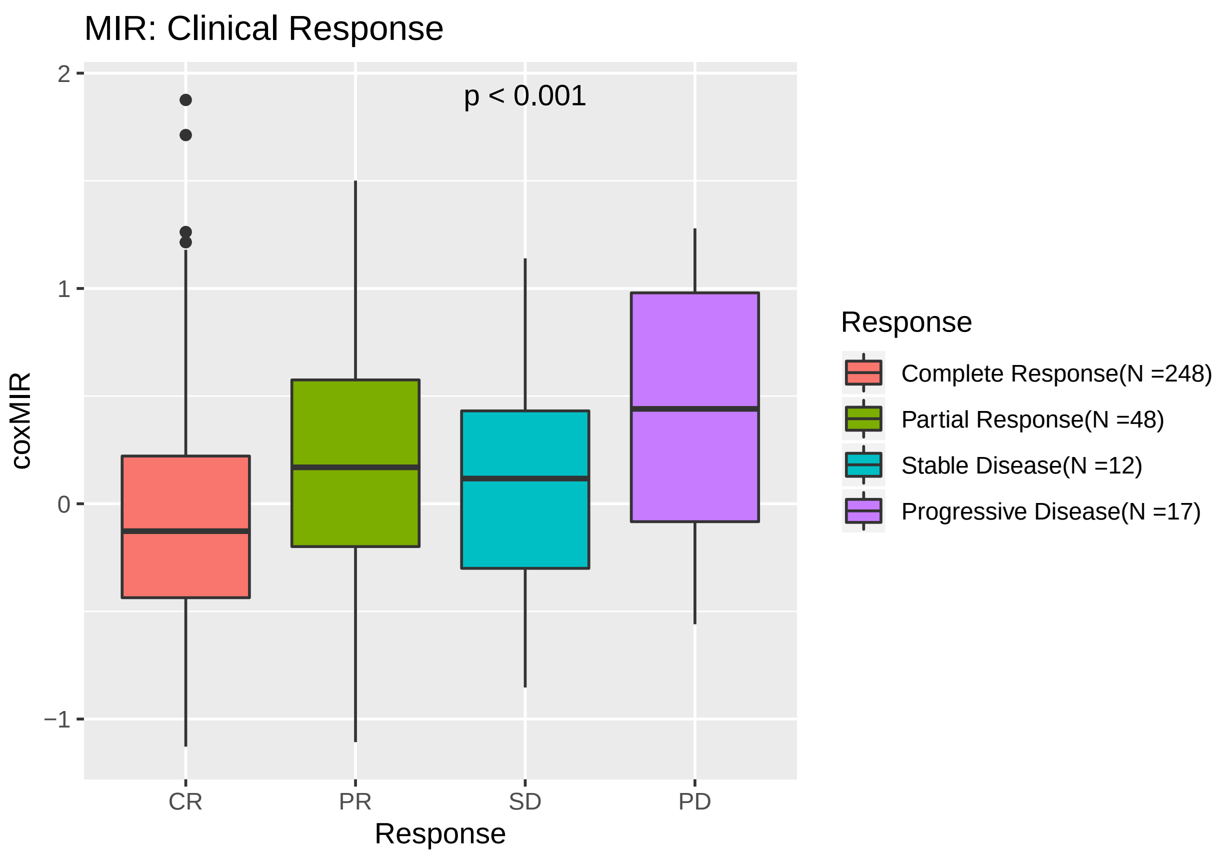


```
crMIRIPI
```

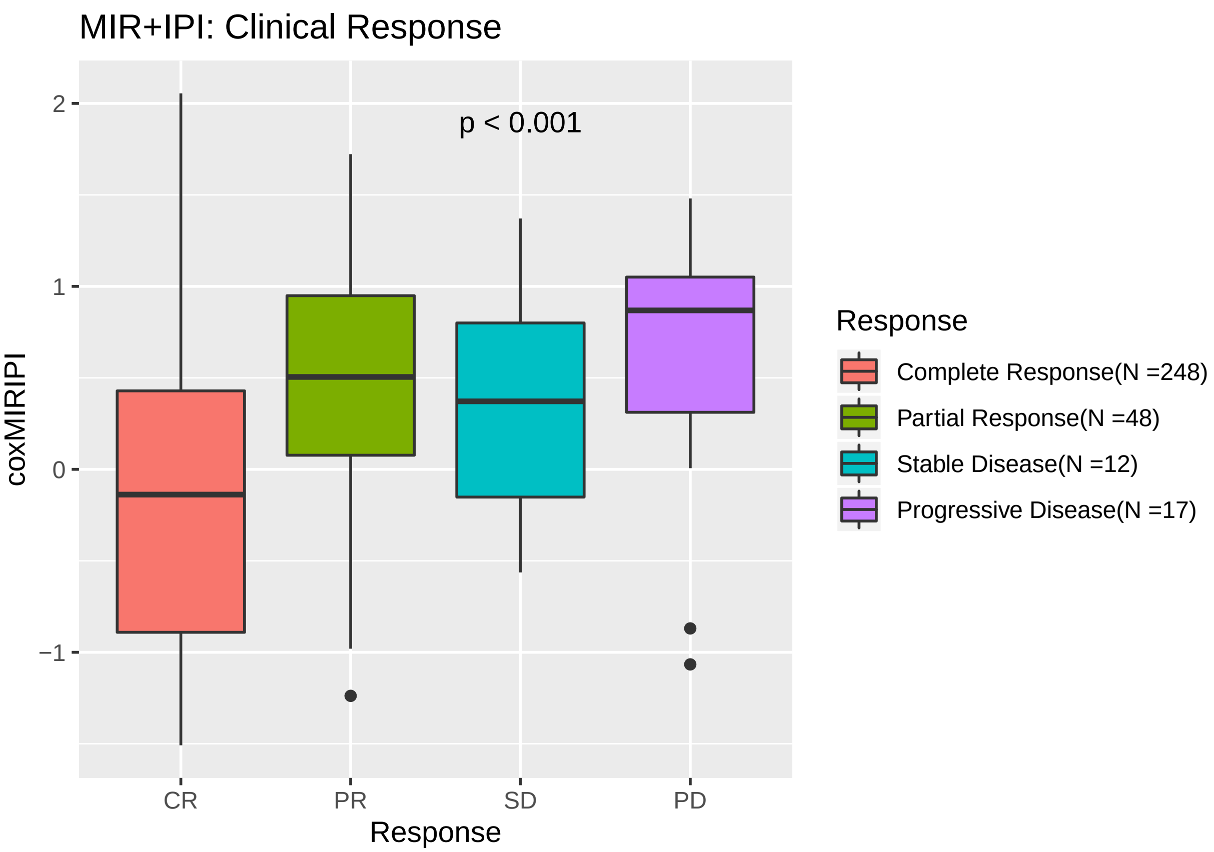

Combined side-by-side plot

```
crMIR <- crMIR + scale_fill_discrete(guide = FALSE) + ylim(c(-1.5, 2.2)) + labs(tag = "A")

## Scale for 'fill' is already present. Adding another scale for 'fill',
## which will replace the existing scale.

crMIRIPI <- crMIRIPI + ylim(c(-1.5, 2.2)) + labs(tag = "B")
fig4 <- grid.arrange(crMIR, crMIRIPI, ncol = 2, widths = c(1,1.8))

## Warning: Removed 1 rows containing non-finite values (stat_boxplot).
```

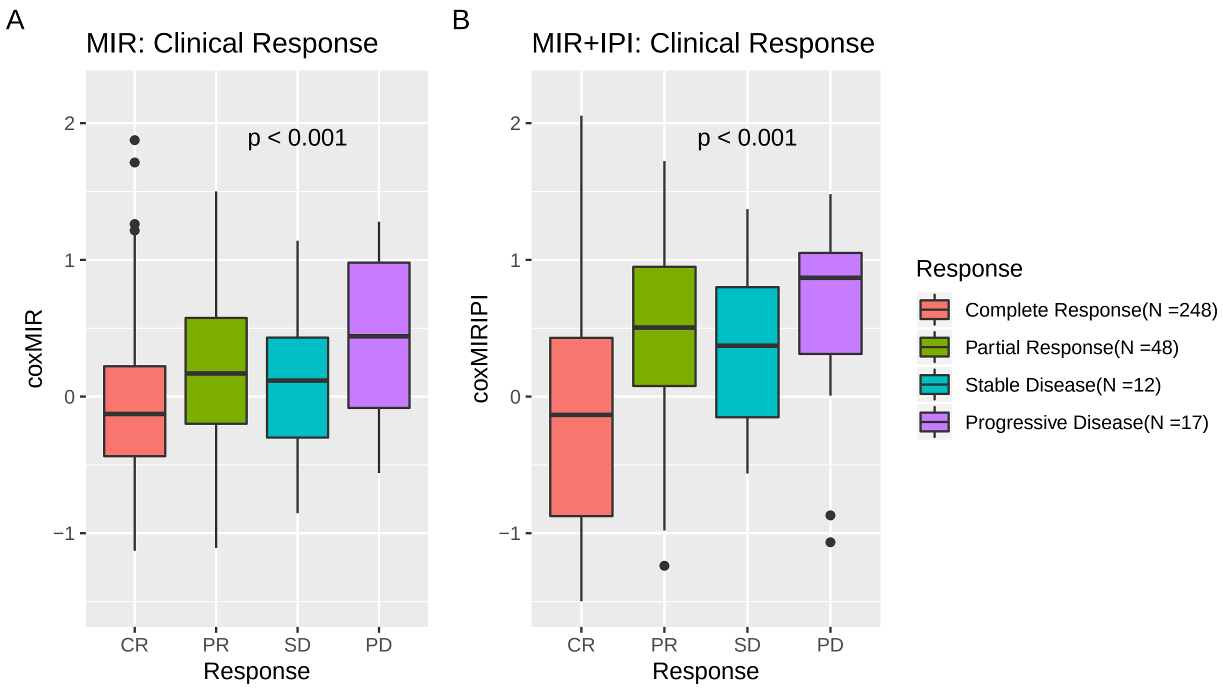


```r
plot(fig4)
```

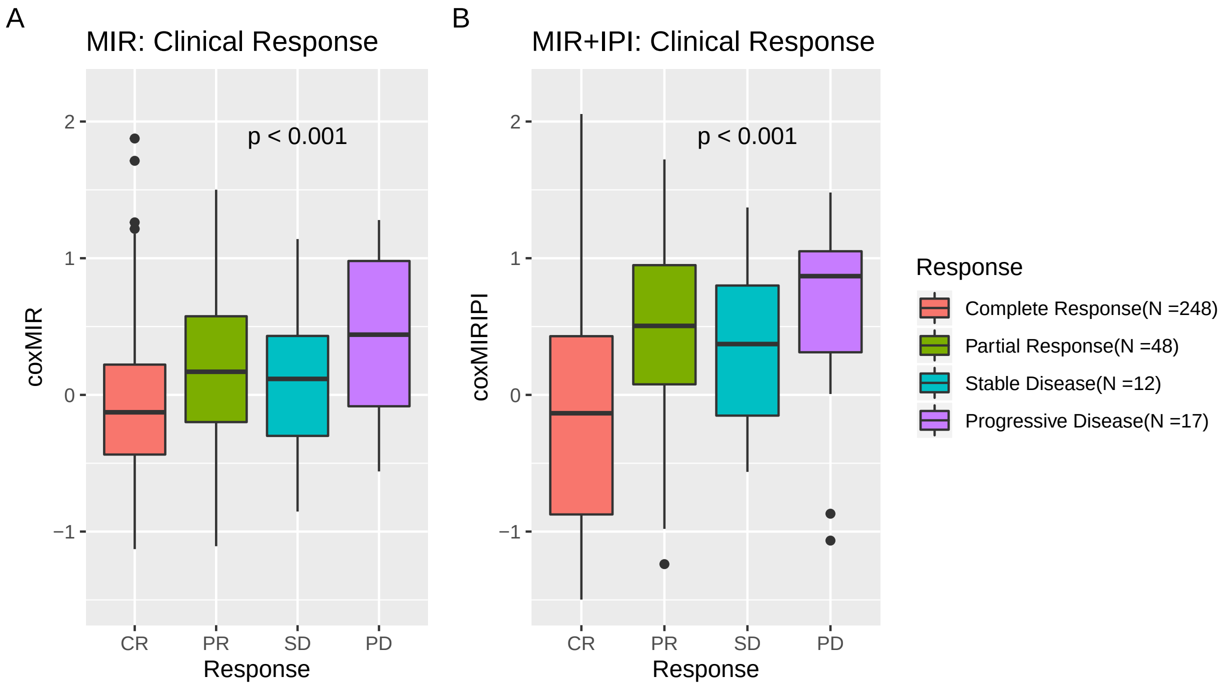

Train final classifer

Based on results above we train a classifier with MIR probes and IPI in the full GCB subset of data Build dataset

```r
cox.data <- data.frame(t(combined.data.combat[probes, ]))
names(cox.data) <- probes
cox.data <- cbind(combined.metadata, cox.data)
cox.data.gcb <- subset(cox.data, ABCGCB == "GCB")
```

Do the univariate cox regression for each probe

```r
cox.gcb.PFS <- uni.multi.cox(cox.data.gcb, probes, outcome = "PFS")
kable(cox.gcb.PFS$combined.results)
```

	MIR	uni.HR	uni.conf	uni.p	multi.HR	multi.conf	multi.p
238225_at	MIR146A	0.92	(0.49;1.71)	0.792	(0.61;2.14)	(0.61;2.14)	0.669
232504_at	MIR146A	0.69	(0.58;0.81)	0.000	(0.65;1)	(0.65;1)	0.047
229437_at	MIR155	0.75	(0.66;0.85)	0.000	(0.73;1.04)	(0.73;1.04)	0.120
220990_s_at	MIR21	0.74	(0.62;0.88)	0.001	(0.79;1.39)	(0.79;1.39)	0.760
229417_at	MIR21	0.90	(0.55;1.46)	0.666	(0.54;1.42)	(0.54;1.42)	0.591
235317_at	MIR23A	0.98	(0.64;1.5)	0.932	(0.8;2.29)	(0.8;2.29)	0.257
1555847_a_at	MIR23A	0.76	(0.59;0.97)	0.025	(0.61;1.23)	(0.61;1.23)	0.418
235571_at	MIR34A	0.54	(0.38;0.77)	0.001	(0.44;1.02)	(0.44;1.02)	0.064
1557342_a_at	hsa-let-7b	1.02	(0.57;1.84)	0.945	(0.46;1.51)	(0.46;1.51)	0.549
241464_s_at	hsa-let-7b	0.73	(0.33;1.58)	0.422	(0.35;1.62)	(0.35;1.62)	0.464
227488_at	MIR503	1.11	(0.62;1.98)	0.726	(0.57;1.93)	(0.57;1.93)	0.875

```r
## Probes that were significant in univariate cox for PFS
sig.probes <- row.names(cox.gcb.PFS$combined.results[cox.gcb.PFS$combined.results$uni.p<0.05,])

## Pick out necessary columns from GCB subset of data, and recode IPI
cox.final.data <- cox.data.gcb[, c("PFS", "IPI", sig.probes)]
cox.final.data$IPI <- factor(ifelse(cox.final.data$IPI %in% c("0", "1"), "0-1", "2-5"))

## Fit model and print results
cox.final <- coxph(PFS~., data = cox.final.data)
kable(summary(cox.final)$conf.int)
```

	exp(coef)	exp(-coef)	lower .95	upper .95
IPI2-5	3.3345365	0.2998918	1.9559374	5.684810
232504_at	0.8568170	1.1671104	0.6916290	1.061458
229437_at	0.8676394	1.1525525	0.7308004	1.030101
220990_s_at	0.9952559	1.0047667	0.7652018	1.294475
1555847_a_at	0.9578975	1.0439531	0.7233349	1.268524
235571_at	0.7759503	1.2887424	0.5220012	1.153443

Prepare figures for publication

```r
## Single column 3.125" @ 600 dpi
## Double column 7"     @ 600 dpi

tiff("../Output/figures/fig1.tiff", width = 1875, height = 1875, pointsize = 50, compression = "lzw")
  grid.draw(v1)
dev.off()
```

```
## pdf
```

```
##   2
```

```
ggsave("../Output/figures/fig2.tiff", plot = fig2, dpi = 600, width = 7.5, height = 7, compression = "lz
ggsave("../Output/figures/fig3.tiff", plot = fig3, dpi = 600, width = 7.5, height = 7, compression = "lz
ggsave("../Output/figures/fig4.tiff", plot = fig4, dpi = 600, width = 7.5, height = 3.5, compression =
```

Validate MIR expression with ddPCR

Read ddPCR data

```
ddPCR <- read_xlsx("../ExternalData/Cell lines/MiR_exp_ddPCR_correlation_analysis.xlsx")
```

```
## New names:
## * `` -> ...7
```

```
### Extract actual data and probeset to mir names
ddPCRdata <- ddPCR[,1:6]
ddPCRnames <- ddPCR[1:5,8:9]
```

Correlate U133+2 data to ddPCR data

```
Cors <- c()
for(i in 1:nrow(ddPCRnames)){
  Cors[i] <- cor(ddPCRdata[,ddPCRnames$miRNA[i]],
                 bCellu133[ddPCRnames$Probeset[i],])
}
Cors <- data.frame("miR" = ddPCRnames$miRNA, "Correlation" = Cors)
kable(Cors, caption = "Correlation of ddPCR vs U133+2 expression")
```

Table 5: Correlation of ddPCR vs U133+2 expression

miR	Correlation
miR-146a	0.8969691
miR-155	0.9429651
miR-21	0.7759889
miR-27a	0.6526918
miR-34a	0.7322472

Create figure

```
p <- list()
ddPCRdata <- as.data.frame(ddPCRdata)

for(i in 1:nrow(ddPCRnames)){
  mirName <- as.character(ddPCRnames$miRNA[i])
  u133Name <- as.character(ddPCRnames$Probeset[i])
  regression  <- lm(bCellu133[u133Name,] ~ ddPCRdata[,mirName])

  tempPlot <- data.frame("mir" = ddPCRdata[,ddPCRnames$miRNA[i]],
                         "u133" = bCellu133[ddPCRnames$Probeset[i],])

  p[[i]] <- ggplot(tempPlot, aes(x=mir, y=u133)) +
    geom_point(shape=1) +
    geom_smooth(method=lm) +
```

```
    xlab(mirName) +
    ylab(u133Name) +
    ggtitle(mirName) +
    annotate("text", x = -Inf, y = -Inf, hjust = -1, vjust = -1,
             label = paste("R^2 = ", round(summary(regression)$r.squared, 2)))

}

grid.arrange(grobs = p, ncol = 3)
```

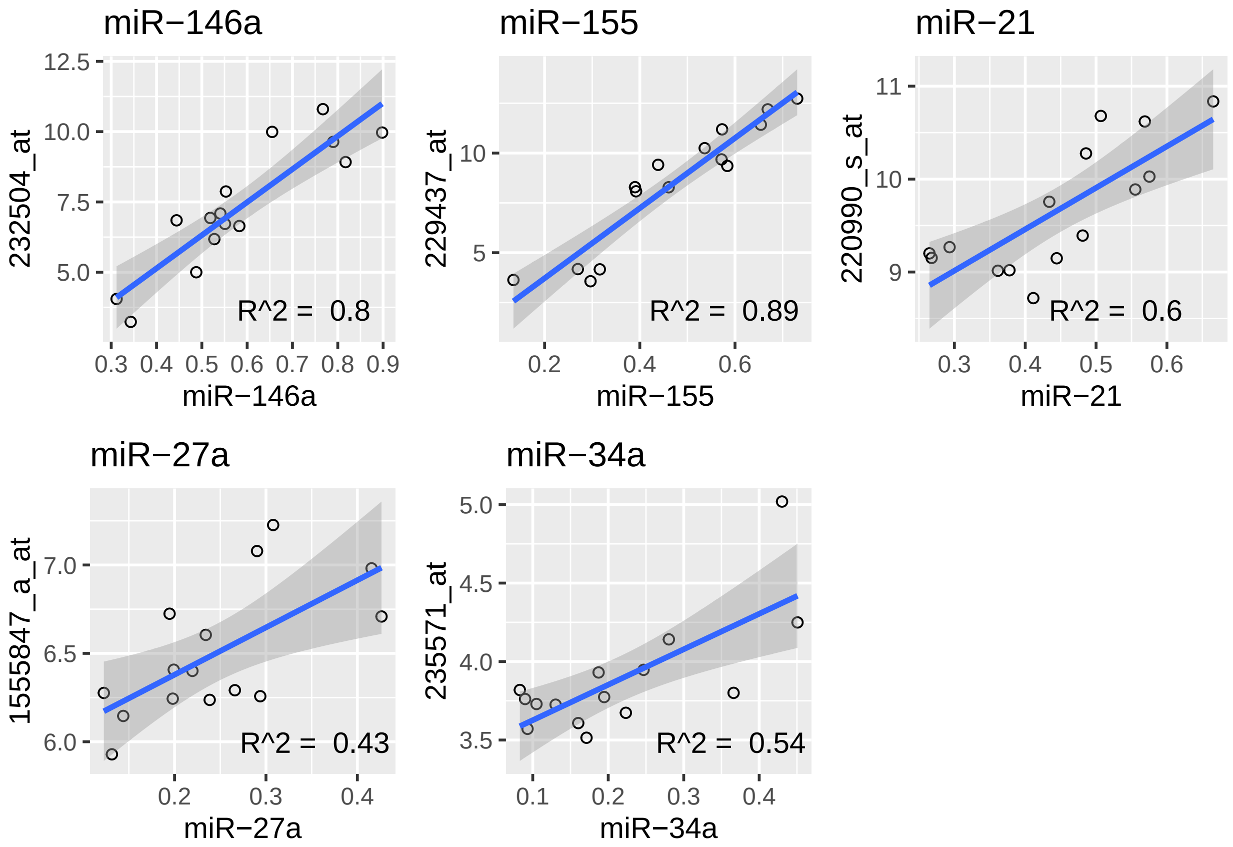

Supplement: Supplementary file 2 — Additional file 2. MicroRNA associated to single drug components of R-CHOP identifies diffuse large B-cell lymphoma patients with poor outcome and adds prognostic value to the international prognostic index. [file 12885_2020_6643_MOESM2_ESM.pdf]
